# The evolution of tenascins

**DOI:** 10.1186/s12862-024-02306-2

**Published:** 2024-09-14

**Authors:** Josephine C. Adams, Richard P. Tucker

**Affiliations:** 1 School of Biochemistry, University of Bristol, Bristol, BS8 1TD UK; 2 Department of Cell Biology and Human Anatomy, University of California, Davis, CA 95616 USA

**Keywords:** Evolution, Extracellular matrix, Tenascin, Phylogeny

## Abstract

**Background:**

The evolution of extracellular matrix is tightly linked to the evolution of organogenesis in metazoans. Tenascins are extracellular matrix glycoproteins of chordates that participate in integrin-signaling and morphogenetic events. Single tenascins are encoded by invertebrate chordates, and multiple tenascin paralogs are found in vertebrates (designated tenascin-C, tenascin-R, tenascin-W and tenascin-X) yet, overall, the evolution of this family has remained unclear.

**Results:**

This study examines the genomes of hemichordates, cephalochordates, tunicates, agnathans, cartilaginous fishes, lobe-finned fishes, ray-finned fishes and representative tetrapods to identify predicted tenascin proteins. We comprehensively assess their evolutionary relationships by sequence conservation, molecular phylogeny and examination of conservation of synteny of the encoding genes. The resulting new evolutionary model posits the origin of tenascin in an ancestral chordate, with tenascin-C-like and tenascin-R-like paralogs emerging after a whole genome duplication event in an ancestral vertebrate. Tenascin-X appeared following a second round of whole genome duplication in an ancestral gnathostome, most likely from duplication of the gene encoding the tenascin-R homolog. The fourth gene, encoding tenascin-W (also known as tenascin-N), apparently arose from a local duplication of *tenascin-R*.

**Conclusions:**

The diversity of tenascin paralogs observed in agnathans and gnathostomes has evolved through selective retention of novel genes that arose from a combination of whole genome and local duplication events. The evolutionary appearance of specific tenascin paralogs coincides with the appearance of vertebrate-specific cell and tissue types where the paralogs are abundantly expressed, such as the endocranium and facial skeleton (tenascin-C), an expanded central nervous system (tenascin-R), and bone (tenascin-W).

**Supplementary Information:**

The online version contains supplementary material available at 10.1186/s12862-024-02306-2.

## Background

The extracellular matrix (ECM) is a defining feature of the complex, multicellular tissues and organs of metazoans. While some of the families of ECM molecules that are best known from chordate models are present in modern sponges (Porifera; e.g., fibrillar and basement membrane collagens, laminins and thrombospondins), and thus have Precambrian origins, others are thought to have appeared during or just before the Cambrian radiation (estimated to have taken place approximately 538 million years ago) and are found only in chordates [1–6]. ECM molecules considered specific to Phylum Chordata include fibronectin and tenascins [7], which act as integrin ligands and participate in the regulation of morphogenetic events such as the migration of neural crest cells, the differentiation of dense connective tissue, the development of general immunity, and the maintenance of stem cell niches [8–11].

**Fig. 1:**
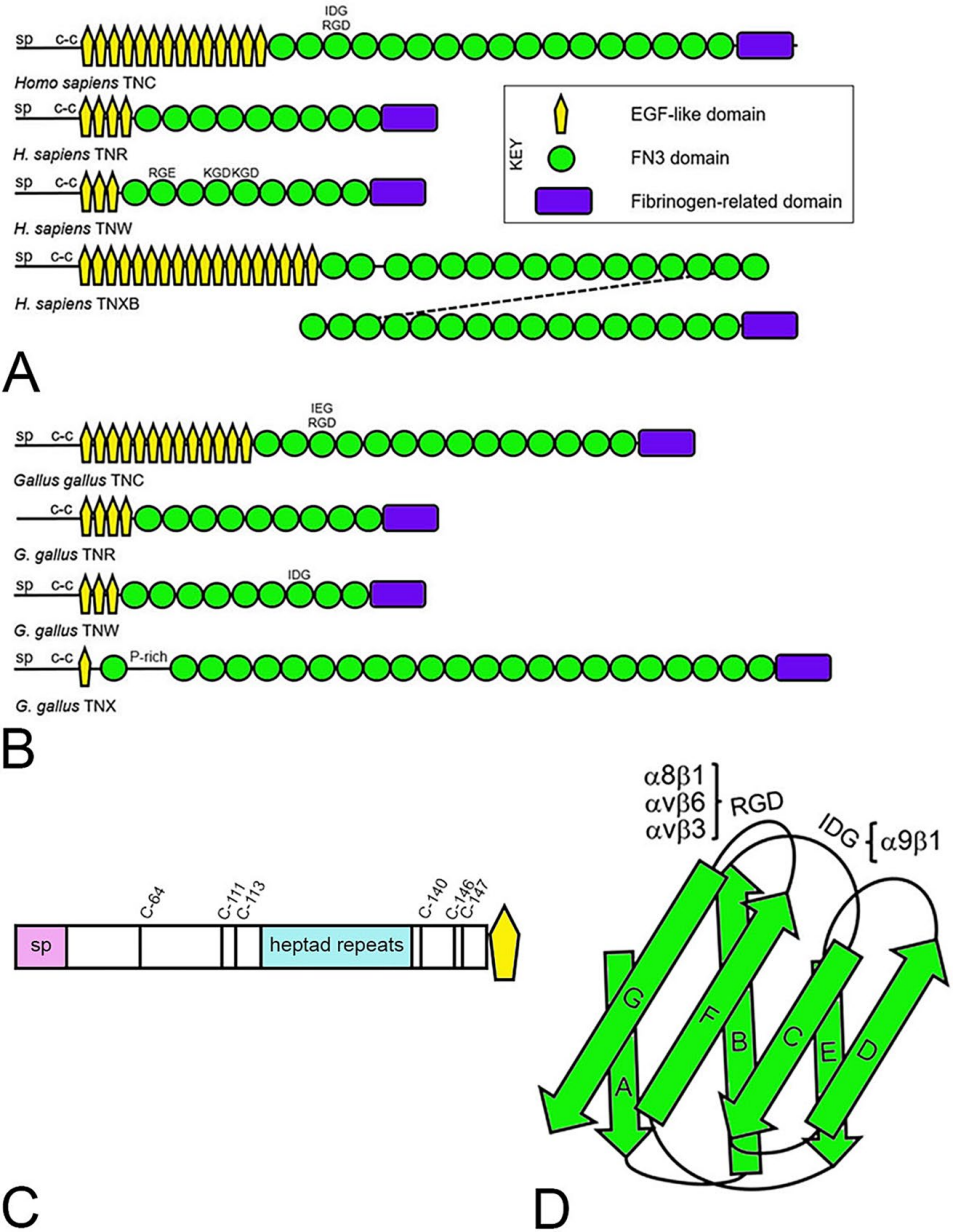
**A**. Schematic depiction of tenascin domain architecture from *Homo sapiens*. The four tenascin paralogs, tenascin-C (TNC), tenascin-R (TNR), tenascin-W (TNW) and tenascin-XB (TNXB), each has a signal peptide (sp) and heptad repeats that form a coiled-coil (c-c), followed by tenascin-type epidermal growth factor (EGF)-like domains, fibronectin type 3 (FN3) domains, and a C-terminal fibrinogen-related domain. Potential integrin-binding tripeptide amino acid motifs in the FN3 domains are indicated. **B**. The domain organization of the four tenascin paralogs found in the chicken *Gallus gallus*. P-rich = proline-rich. **C**. Schematic representation of the N-terminal region of *H. sapiens* TNC, showing signal peptide (sp), tenascin assembly domain with location of cysteine residues implicated in forming or stabilizing multimers and heptad repeats, and first EGF-like domain. **D**. The third FN3 domain of *H. sapiens* TNC is formed by seven β strands lettered A through G. (adapted from [116]). The integrin-binding motif arginine-glycine-aspartic acid (RGD) is present in an exposed loop between β strands F and G, and the integrin-binding motif isoleucine-aspartic acid-glycine (IDG) is present in an exposed loop between β strands **B** and **C**. The integrin receptors that recognize these motifs are indicated

Mammalian and avian genomes encode four tenascin paralogs: tenascin-C (TNC), tenascin-R (TNR), tenascin-W (TNW; also known as tenascin-N or TNN) and tenascin-X (TNX), all of which have a common domain architecture (Fig. 1A, B) [12]. An amino-terminal signal peptide directs each protein to the secretory pathway, and this is followed by a tenascin assembly domain comprised, in TNC, of a region that includes conserved cysteine residues followed by a short heptad-repeat region and additional conserved cysteine residues (Fig. 1C). TNC is secreted as hexabrachions that require inter-subunit disulfide bonds for assembly [13] and form rapidly [14]. By expression of assembly domain recombinant proteins in *Escherichia coli*, TNC oligomerization was shown to be a two-step process, involving trimerization by the heptad-repeat region with stabilization by cysteines C-111 and C-113 [14] followed by cross-linking of two trimers into the hexamer, in which C-64 has a critical role [15, 16]. TNW also assembles into six-armed hexabrachions [17, 18], whereas TNR appears as trimers [19, 20]. Purified chicken TNX is a monomer when viewed in the electron microscope [21], although recombinant expression of TNX in HEK293 cells resulted in detection of trimers [22]. The tenascin assembly region is followed by one or more epidermal growth factor (EGF)-like domains and tandem repeated fibronectin-type 3 (FN3) domains. A single fibrinogen-related domain (FReD) is found at the carboxy terminus. The EGF-like domains of tenascins have distinctive spacing between conserved cysteine and glycine residues (xC[x]_3_C[x]_5_C[x]_4-6_CxC[x]_5_G[x]_2_Cx, where x is any amino acid) that set them apart from other members of this domain family. FN3 domains are often encoded on a single exon, which appears to contribute to a broad range in their number in orthologous tenascins across species belonging to the same phylogenetic family [12], as well as the potential for functional diversity through alternative splicing [8, 23].

Like fibronectin, some tenascins are ligands that interact with ECM integrin receptors via short amino acid motifs found in exposed loops between β strands in their FN3 domains (Fig. 1D). The best studied of these is the arginine-glycine-aspartic acid (RGD) motif found in the loop between β strands F and G of the third FN3 of TNC, which can interact with α8β1, αvβ6 and αvβ3 integrins [24]. An isoleucine-aspartic acid-glycine (IDG) motif in the loop between β strands B and C, also in the third FN3 of TNC, binds to α9β1 integrin (Fig. 1D) [25]. Similar exposed motifs are found in FN3 domains of TNW, and there is some experimental evidence that these also participate in integrin recognition [18]. Function-blocking antibodies to β1 integrin inhibit the proliferative effect of recombinant FN3 domains of TNR on cultured neural stem cells [26], but the specific region in TNR that interacts with the integrin has yet to be mapped. The amino acid sequence of the FReD is well conserved across the four paralogs [27]. There is experimental evidence that the FReD can also bind to integrins [28, 29], and that it acts as a ligand for Toll-like receptor 4 [30].

TNC was the first member of the tenascin gene family to be discovered and is also the best studied [8, 31, 32]. TNC plays roles in branching morphogenesis and the differentiation and migration of many cell types, especially in the nervous system. It is more widely distributed during embryonic development than in the adult, but it persists in some dense connective tissues and stem cell niches [9] and reappears during chronic inflammation and tumorigenesis [33]. *TNR* expression is limited to the nervous system and suprarenal glands, and studies with *TNR* knockout mice point to an important role for this tenascin in neurogenesis and the maintenance of perineuronal nets [34]. Immunohistochemistry reveals TNW in a subset of the ECM where TNC is also found, but it is most abundant in bone [35]. In vitro, TNW can promote the development of bone [36], and the *tnn* knockout mouse (the official gene names for *TNW* in humans and mouse are *Tnn* and *tnn*, respectively) has defects in alveolar bone and incisor remodeling [37]. Finally, TNX is found primarily in skeletal muscle and loose connective tissue [38] where it can bind to type I collagen and influence collagen fibril formation [39]. Mutations in *TNX* are linked to Ehlers-Danlos Syndrome [40].

Our previous work addressing the evolution of tenascins showed that a tenascin is present in the tunicate *Ciona intestinalis* [12] and in a cephalochordate, *Branchiostoma floridae* [41]. Whereas these species each have a single tenascin-encoding gene, two tenascin genes are present in the cartilaginous ghost shark *Callorhincus milii*, and the encoded proteins are most closely related to to tetrapod TNC and TNR [7]. At the time these studies were carried out, no tenascin could be identified in the echinoderm, *Strongylocentrotus purpuratus*, suggestive of a possible chordate-specific origin of tenascins.

With the benefit of a greater number of sequenced genomes and transcriptomes from non-chordate deuterostomes and early diverging chordate species, the goal of the current study was to examine the origin of tenascins and make an in-depth assessment of the molecular evolution of tenascins. To achieve this, genomes of echinoderms, hemichordates and a xenacoelomorph were examined for encoding of possible tenascin-like proteins, and predicted amino acid sequences of tenascins were identified from diverse members of Phylum Chordata, with emphasis on the recently sequenced and assembled genomes of agnathans, sharks and rays, along with species of lobe-finned and ray-finned fishes. Many of these have undergone multiple rounds of whole genome duplication (WGD) that occurred separately from the two rounds of WGD associated with the base of the vertebrate lineage [42]. In our study, tenascins were first identified by protein sequence characteristics and domain organization, and the assembly domain and potential integrin recognition motifs were analyzed in detail. Phylogenetic tree analysis of the FReDs was used to further examine relationships of the tenascin family members and to inform models of tenascin evolution. The identification and evolutionary relationships of tenascin family members were also examined by criteria of synteny. Identification of conservation of synteny (i.e., conservation of linkage of orthologous gene loci in different species, notwithstanding the exact gene order on a chromosome or assembled genomic region, or inclusion of non-conserved intervening genes) is an effective independent method to examine evolutionary relationships in multi-gene families [43] and also informed the evolutionary model presented herein.

## Methods

### Assembled genomes

The assembled genomes of the following species were analyzed for tenascins and syntenic relationships with tenascin genes (see Results for additional citations): *Acipenser ruthenus* Sterlet, NCBI Assembly ASM1064508v2 [44]; *Amblyraja radiata* Thorny skate, NCBI Assembly sAmbRad1.1.pri; *Anolis carolinensis* Green anole, NCBI Assembly AnoCar2.0 [45]; *Branchiostoma belcheri* Belcher’s lancelet, NCBI Assembly Haploidv18h27 [46]; *Branchiostoma floridae* Florida lancelet, NCBI Assembly Bfl_VNyyK [47]; *Branchiostoma lanceolatum* Amphioxus, NCBI Assembly BraLan3 [48]; *Callorhinchus milii* Elephant (ghost) shark, NCBI Assembly IMCB_Cmil_1.0 [49]; *Carcharodon carcharias* Great white shark, NCBI Assembly sCarCar2.pri; *Ciona intestinalis* Vase tunicate, NCBI Assembly KH [50]; *Danio rerio* Zebrafish, NCBI Assembly GRCz11 [51]; *Eptatretus burgeri* Inshore hagfish, eEnsembl Assembly Eburgeri_3.2 (submitted by the Riken Center For Development Biology); *Gallus gallus* Chicken, NCBI Assembly bGalGal1.mat.broiler.GRCg7b [52]; *Homo sapiens* Human, NCBI Assembly GRCh37.p13 [53]; *Latimeria chalumnae* Coelacanth, NCBI Assembly fLatCha1.pri, Annotation Release GCF_037176945.1-RS_2024_04; *Lytechinus pictus* Painted urchin, NCBI Assembly UCSD_Lpic_2.1; *Lytechinus variegatus* Green sea urchin, NCBI Assembly Lvar_3.0; *Mus musculus* House mouse, NCBI Assembly GRCm39 [54]; *Oikopleura dioica* Pelagic tunicate sp., NCBI Assembly OKI2018_I68_1.0 [55]; *Oncorhynchus tshawytscha* Chinook salmon, NCBI Assembly Otsh_v2.0 [56]; *Petromyzon marinus* Sea lamprey, NCBI Assembly kPetMar1.pri [57]; *Pleurodeles waltl* Iberian ribbed newt, NCBI Assembly ASM2665232v1 [58]; *Polyodon spathula* Mississippi (American) paddlefish, NCBI Assembly ASM1765450v1 [59]; *Polypterus senegalus* Gray (Senegal) bichir, NCBI Assembly ASM1683550v1 [60]; *Protopterus annectens* West African lungfish, NCBI Assembly PAN1.0; *Rhincodon typus* Whale shark, NCBI Assembly sRhiTyp1.1; *Saccoglossus kowalevskii* Acorn worm sp., NCBI Assembly Skow_1.1 [61]; *Scyliorhinus canicula* Small-spotted catshark, NCBI Assembly sScyCan1.1; *Stegostoma fasciatum/tigrinum* Zebra shark, NCBI Assembly sSteTig4.hap1; *Strongylocentrotus purpuratus* Purple sea urchin, NCBI Assembly Spur_5.0; *Thunnus maccoyii* Southern bluefin tuna, NCBI Assembly fThuMac1.1 [62]; *Xenopus laevis* African clawed frog, NCBI Assembly Xenopus_laevis_v10.1 [63]; *Xenopus tropicalis* Tropical clawed frog, NCBI Assembly UCB_Xtro_10.0 [64].

### Programs, search tools and phylogenetic trees

The following tasks were performed with these programs. Genome data browsers: NCBI genome data viewer (https://www.ncbi.nlm.nih.gov/genome/gdv/) [65], eEnsembl genome browser (https://www.ensembl.org) [66]; phylogenetic tree makers and viewers: EMBL Toolkit (http://etetoolkit.org/treeview/) [67], Centre National de la Recherche Scientifique (http://www.phylogeny.fr/advanced.cgi) (https://ngphylogeny.fr/) [68], T-REX (http://www.trex.uqam.ca/index.php) [69]; protein domain identification: SMART (http://smart.embl-heidelberg.de/) [70]; InterPro 98.0 (https://www.ebi.ac.uk/interpro/search/sequence/) [71]; Sequence LOGOs were generated from MUSCLE sequence alignments in WebLogo 3 (https://weblogo.threeplusone.com) [72]; coiled-coil prediction: Marcoil, Multicoil2, Ncoils, Parcoil2 (https://waggawagga.motorprotein.de/) [73]; secondary structure prediction: J Pred 4 (https://www.compbio.dundee.ac.uk/jpred/) [74]; protein blast: NCBI blastp (https://blast.ncbi.nlm.nih.gov/Blast.cgi) [75]; multiple sequence alignment by MUSCLE version 3.8.31, with ClustalW output format [76]: EMBL EBI (https://www.ebi.ac.uk/jdispatcher/msa/muscle) [77]; Protein expression tool: (https://www.xenbase.org/xenbase/) [78].

To construct phylogenetic trees at NGPhylogeny.fr (see above) files were uploaded in FASTA format and aligned with MAFFT v7 (gap penalty 0.123; gap opening penalty default 1.53; no matrix selection), alignment curated by BMGE (estimated matrix BLOSUM 62; default sliding window size 3; maximum entropy threshold 0.5; gap rate cutoff 0.5; minimum block size 5), analyzed with PhyML v3 (evolutionary model LG; equilibrium frequencies ML/model; estimated proportion of invariant sites; 4 categories for discreet gamma model; subtree pruning and regraphing; branch support with aBayes), and resulting trees were rendered with Newick.

### Identification of tenascins, synteny and domains

Putative tenascin sequences were identified by blastp (see above) using previously identified sequences from a related species while limiting the search to the target species or taxa. Predicted amino acid sequences with tenascin domain architectures were selected for additional analysis. If annotated genomes are available, they were either searched with amino acid sequences of tenascins from related taxa or with the term ‘tenascin’ using the search field. These searches were also used to identify neighboring genes. For example, to identify tenascins in the small-spotted catshark using the *Scyliorhinus canicula* genome data viewer (https://www.ncbi.nlm.nih.gov/genome/gdv/browser/genome/?id=GCF_902713615.1), the assembly is searched using the term ‘tenascin’. This reveals *TNC* on chromosome 21, *TNR* on chromosome 4, and *TNX* on chromosome 13. An additional hit could be discounted (it is a gene encoding a long string of FN3 domains that is actually a FACIT collagen). The Gene ID feature then allows access to the predicted amino acid sequence in FASTA format for domain analysis, and mapping of neighboring genes. FReD sequences that were aligned with MUSCLE and used for phylogenetic tree construction were identified using SMART (see above). Phylogenetic trees were constructed as described previously [7] following alignment with MUSCLE (see above) and branch support calculated with transfer bootstrap expectation [79].

## Results

### Tenascin precursors in non-chordates?

Using canonical tenascins from several species as BlastP search tools, the genome-predicted proteomes of the echinoderms *Lytechinus variegatus* [80] and *S. purpuratus* [81] were examined for proteins with tenascin-type EGF-like domains combined with FN3 domains and/or FReDs. The predicted proteomes were also searched with the keyword “tenascin”, which can reveal the presence of tenascin-type EGF-like domains, using BlastP with sequences from cephalochordates and tunicates, and with the InterPro domain architecture feature [82]. A BlastP search of “Echinodermata” was also made in NCBI. No candidate sequences encoding tenascin-like proteins were found in these echinoderms, but many proteins containing FReD domains are apparent and also several sequences that contain tenascin-like FN3 domains (e.g., XP_041474432). Similar searches were conducted based on the genome of the xenacoelomorph *Symsagittifera roscoffensis* [83]. As in the echinoderms, predicted proteins were found with FReD domains or FN3 domains, but not both in the same polypeptide. The only proteins with tenascin-type EGF-like domains were predicted teneurins (e.g., XP_063714939.1).

**Fig. 2:**
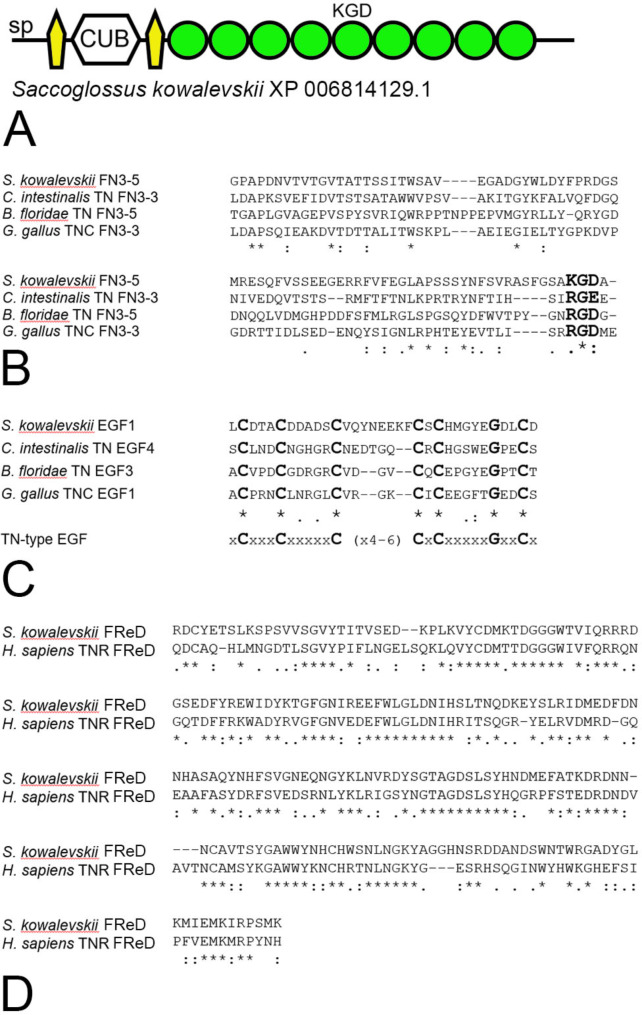
**A.** The tenascin-like predicted protein from the hemichordate *Saccoglossus kowalevskii* (XP_006814129.1). **B**. Multiple sequence alignment (in MUSCLE 3.8) shows alignment of the RGD-like motif lysine-glycine-aspartic acid (KGD) present in the fifth FN3 domain of the tenascin-like protein with the arginine-glycine-glutamic acid (RGE) motif from vase tunicate *Ciona intestinalis* tenascin and the RGD motifs of the Florida lancelet *Branchiostoma floridae* tenascin and chicken *Gallus gallus* TNC. Asterisks indicate identical residues. Conserved and semi-conserved residues are indicated with colons and periods, respectively. **C**. Multiple sequence alignment shows the first EGF-like domain of *S. kowalevskii* tenascin-like protein is a tenascin-type EGF-like domain. **D**. Conservation of the FReD of *S. kowalevskii* XP_002731388 with the FReD of *H. sapiens* TNR

In contrast, the genome of the hemichordate *Saccoglossus kowalevskii* encodes a predicted protein (XP_006814129) with features common to tenascins. This protein has a signal peptide and two EGF-like domains that flank a single CUB domain, followed by nine FN3 domains (Fig. 2A), of which the fifth FN3 domain contains a lysine-arginine-glutamic acid (KGD) motif that aligns with the RGD motif in the fifth FN3 repeat of *B. floridae* tenascin, the RGE motif in the third FN3 domain of *Ciona intestinalis* tenascin, and the RGD motif in the third FN3 domain of TNC from *G. gallus* (Fig. 2B). The first EGF-like domain of the *S. kowalevskii* protein is more similar to tenascin-type EGF-like domains than to other types of EGF-like domains (Fig. 2C), differing from the tenascin-type EGF-like domain consensus sequence only by having eight amino acids between the third and fourth cysteine residues instead of four, five or six, as seen in tenascins from vertebrates. This predicted *S. kowalevskii* protein with tenascin-like features does not include a carboxy-terminal FReD. However, a candidate for a predicted protein with a tenascin-like FReD was identified by BlastP. This short (239 amino acids) predicted protein (XP_002731388) comprises a signal peptide and a FReD that is 45% identical to the FReD of *C. intestinalis* tenascin and is most similar (50% identical) to the FReD of TNR in *H. sapiens* by BlastP analysis (Fig. 2D). For comparison, the human TNR FReD is 60% identical to the human TNC FReD, and 55% identical to the FReD from human TNXB.

### Tenascins of invertebrate chordates

**Fig. 3:**
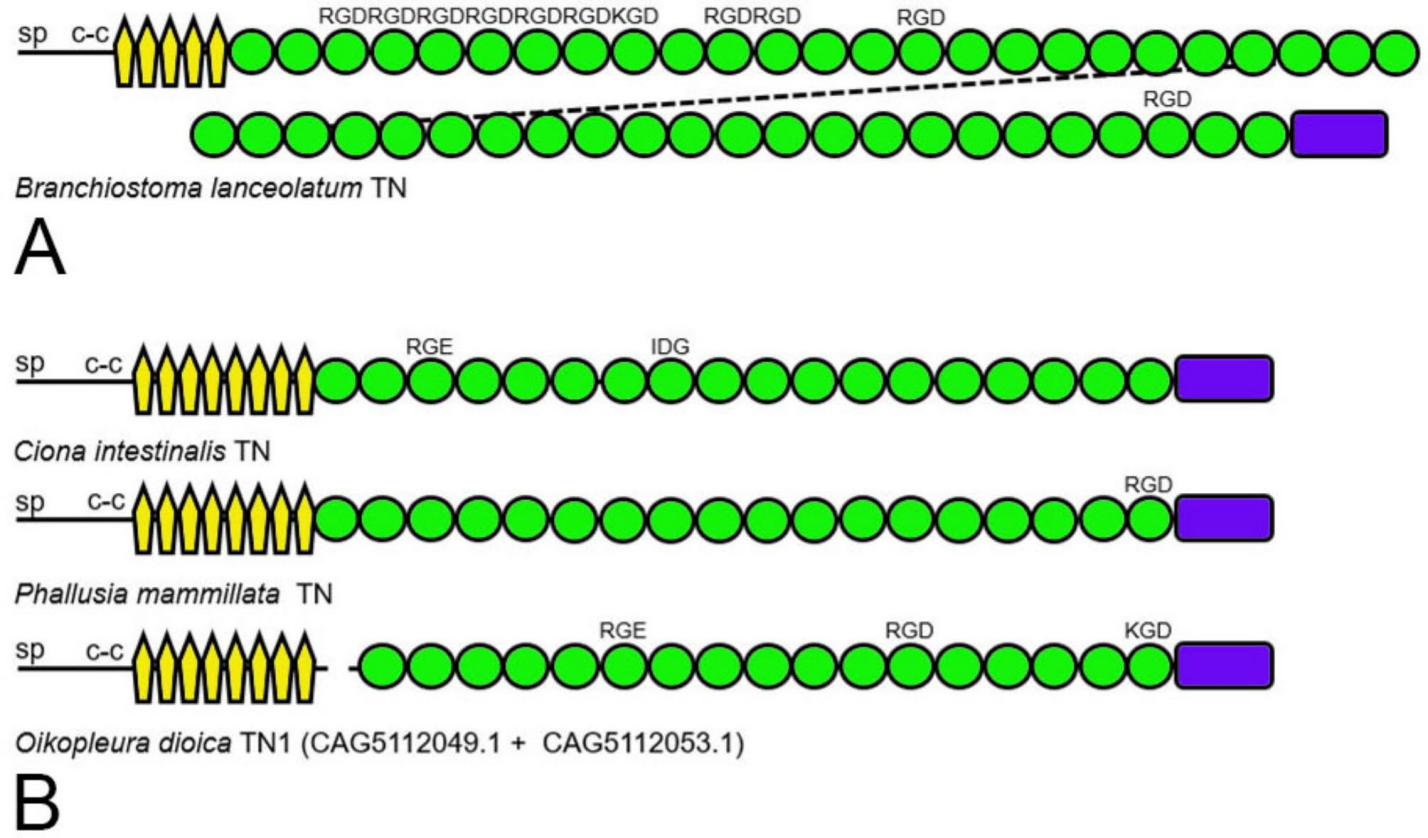
Representative tenascins from invertebrate chordates. **A**. The domain architecture and putative integrin-binding motifs of tenascin (CAH1272042) from the European lancelet *Branchiostoma lanceolatum*. **B**. Tenascins from three tunicates: the vase tunicate *Ciona intestinalis* (XP_018668975), the white warty sea squirt *Phallusia mammillata* (CAB3267154) and the pelagic tunicate *Oikopleura dioica* (CAG5112053 and CAG5112049)

A predicted tenascin was previously described from the genome of the cephalochordate *B. floridae* (XP_035684441) [41]. This large (3947 amino acid) predicted protein clearly has a tenascin domain architecture: the signal peptide is followed by a coiled-coil region, five tenascin-type EGF-like domains, 35 FN3 domains, and a carboxy-terminal FReD. The second, fifth and 33rd FN3 domains have RGD motifs in exposed loops between β strands F and G. In our current study, additional predicted tenascins were identified in the genomes of related lancelets. The European lancelet *B. lanceolatum* has a similar predicted tenascin (CAH1272042) albeit with even more FN3 domains that contain RGD and KGD motifs (Fig. 3A). A predicted tenascin from *B. belcheri* (XP_019643298) has 41 FN3 repeats, ten of which have RGD motifs in the exposed loop between β strands F and G (not shown).

The tunicate *C. intestinalis* has a single tenascin (XP_018668975; Fig. 3B) [12] with an RGD-like arginine-glycine-glutamic acid (RGE) motif in the appropriate exposed loop in its third FN3 domain, and an IDG motif in the exposed loop between β strands B and C of its eighth FN3 domain. With the benefit of additional sequenced urochordate genomes, a tenascin with the same numbers of EGF-like domains and FN3 domains and a single RGD motif was identified in the knobby tunicate *Phallusia mammillata* (CAB3267154; Fig. 3B). The planktonic tunicate *Oikopleura dioica* also appears to have a very similar tenascin, albeit this is currently predicted from two adjacent predicted proteins (CAG5112053 and CAG5112049; Fig. 3B). Though only one tenascin gene is present in *C. intestinalis* and *P. mammillata*, *O. dioica* may also encode an additional potential tenascin (CBY43776). However, at present, this prediction is a partial protein sequence that lacks a signal peptide and encodes only four FN3 repeats and a FReD.

### Tenascins of agnathans

**Fig. 4:**
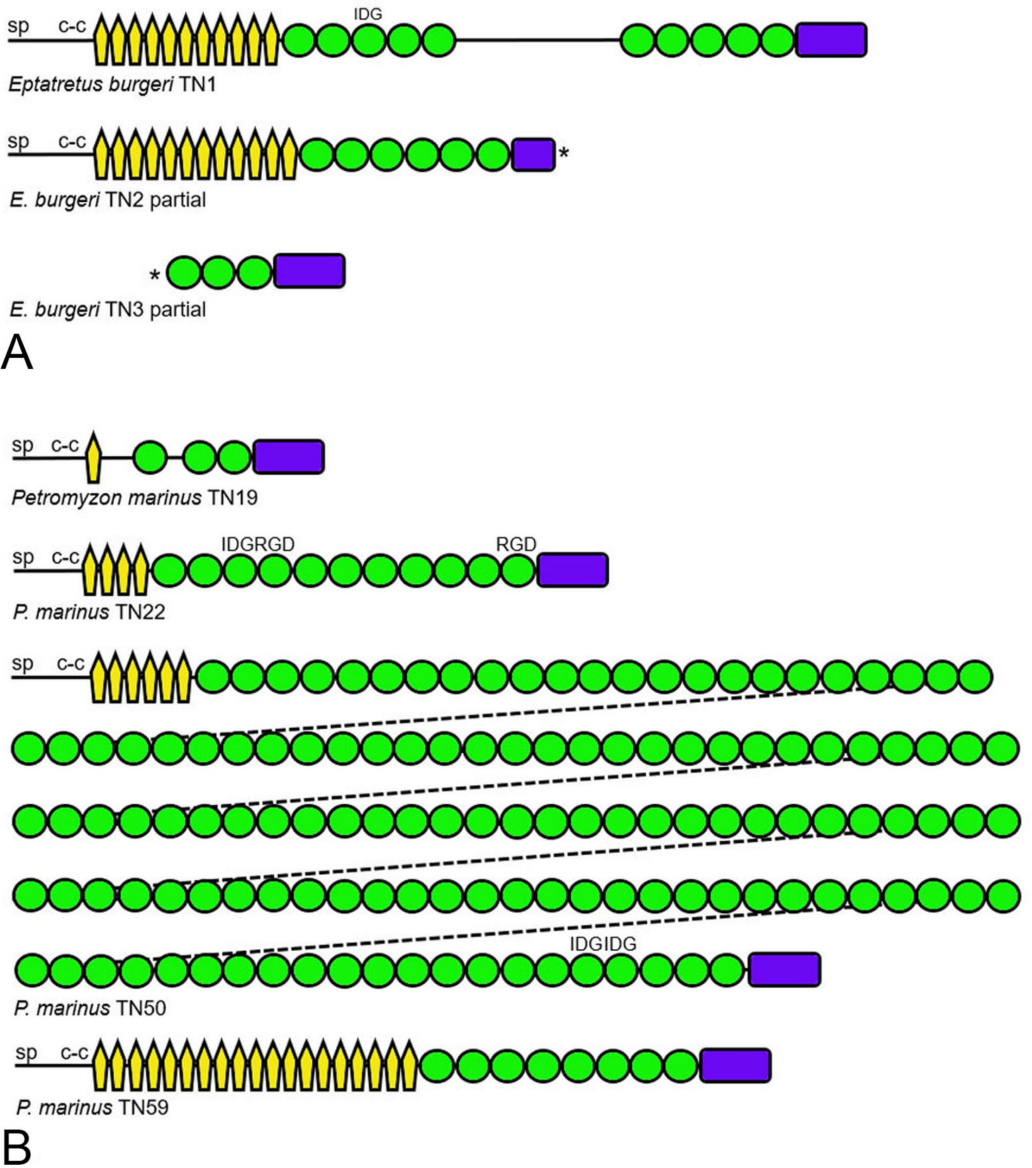
Tenascins from jawless vertebrates. **A**. The domain architecture of one complete and two partial tenascins from the inshore hagfish *Eptatretus burgeri*. Asterisks indicate incomplete sequence (TN1, ENSEBUT00000017267.1; TN2, ENSEBUT00000025135.1; TN3, ENSEBUT00000017267.1). **B**. The four tenascins from the sea lamprey *Petromyzon marinus*. Tripeptide motifs predicted to be exposed to integrin binding are indicated (TN19, XP_032836628; TN22, XP_032814597; TN50, XP_032829135; TN59, XP_032832304)

The genome-predicted proteomes of two jawless vertebrates, the inshore hagfish *Eptatretus burgeri* and the sea lamprey *Petromyzon marinus*, were examined for tenascins. Three tenascins were identified in the hagfish (Fig. 4A). The first (designated TN1) appears to be a complete predicted protein sequence (ENSEBUT00000017267.1) located on Contig FYBX02010759.1 (3,054,167–3,231,455). This tenascin includes a signal peptide and a coiled-coil region, 11 tenascin-type EGF-like domains, 10 FN3 domains (two groups of five separated by disorder), and a carboxy-terminal FReD. An IDG motif is found in the loop between β strands B and C of the third FN3 domain. The second tenascin (TN2; ENSEBUT00000025135.1) is found on Contig FYBX02009657.1 (2,625,311–2,796,592). In addition to a signal peptide and coiled-coil region, it has 12 EGF-like domains, six FN3 domains and a FReD. It lacks RGD, IDG or related motifs in exposed loops, and the predicted FReD sequence appears to be incomplete at this time. The third putative tenascin (TN3; ENSEBUT00000017267.1; also located on Contig FYBX02010759.1 at 2,022,784–2,547,224) is a partial sequence encoding three FN3 domains and a FReD.

Four tenascins were identified in the genome of the sea lamprey *P. marinus* (Fig. 4B). A small (1067 amino acids) tenascin is encoded by a gene on chromosome 19, and therefore is designated TN19 (XP_032836628). This tenascin is predicted to have a single EGF-like domain, three FN3 domains and a FReD. The second, named TN22 (XP_032814597) for the gene location on chromosome 22, has a more traditional tenascin domain organization as well as an IDG motif in the third FN3 domain and RGD motifs in the fourth and 11th FN3 domains. The third predicted sea lamprey tenascin, TN50 (the encoding gene is on chromosome 50; XP_032829135), is predicted as an extremely long polypeptide, with over 18,000 residues and a predicted molecular mass of 1.96 million. TN50 corresponds to the previously reported partial tenascin sequence ENSPMAT00000005710 [7]. The complete sequence includes six EGF-like domains and 131 FN3 domains, many of which have identical amino acid sequences, and a FReD. It lacks RGD motifs, but there are IDG motifs potentially available for integrin binding in the 126th and 127th FN3 domains. A motif of unknown significance (glutamic acid-leucine-valine-isoleucine-serine or ELVIS) is represented in the β strand A of over 100 of the FN3 domains found in TN50. The fourth *P. marinus* tenascin, TN59 (on chromosome 59; XP_032832304), has 19 EGF-like domains, eight FN3 domains that lack known integrin recognition motifs, and a carboxy-terminal FReD.

### Tenascins of cartilaginous fishes

The tenascins of the ghost shark *Callorhinchus milii* (Class Chondrichthyes, Subclass Holocephali) were previously identified by criteria of BLASTP searches, domain analyses and FReD phylogenetic trees as most similar to TNC and TNR [7] (Supplementary Table S1, Tenascins from representatives of the Class Chondrichthyes). Recently, the genome of a second holocephalan, the small-eyed rabbitfish *Hydrolagus affinis*, was sequenced [84]. We were able to identify sequences that are likely to encode the FReDs and adjacent FN3 domains most similar to TNC (Sequence ID JAAILG010061815; ranges 884–1033, 1286–1576, 2095–2283) or TNR (Sequence ID JAAILG010020454; ranges 3070–3138, 4419–4589, 5138–5419, 6022–6141, 6468–6749, 7346–7471) in this chimaera genome. However, until a fully annotated genome is available it is premature to rule out the presence of other tenascin genes in *H. affinis.*

**Fig. 5:**
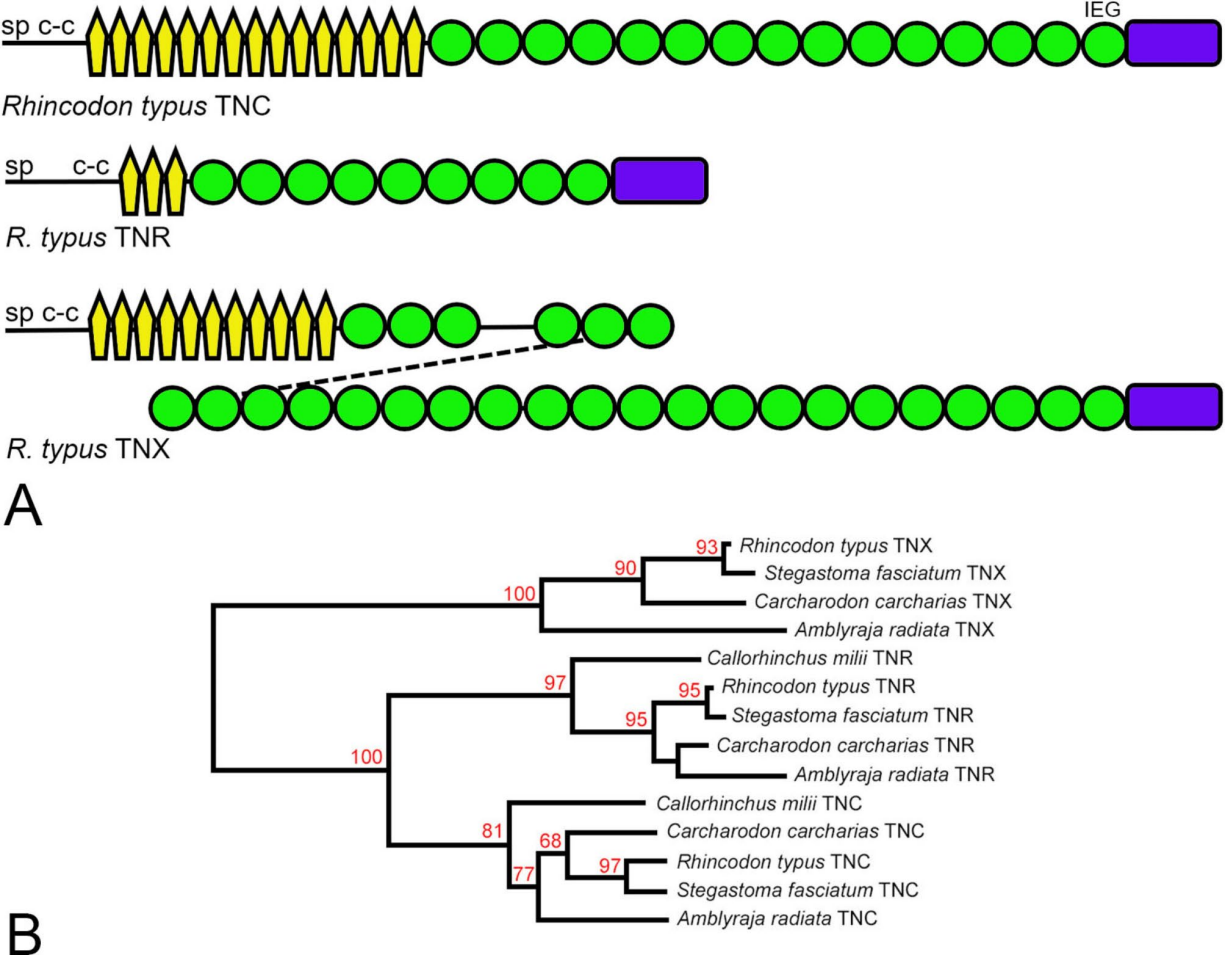
**A.** Schematic models of the three tenascin paralogs of the whale shark *Rhincodon typus*, that are typical of the sharks and rays (Subclass Elasmobranchii; TNC, XP_048469310; TNR, XP_048458101; TNX, XP_048472635 and XP_020378480). **B**. A phylogenetic tree based on the amino acid sequences of the FReDs from representative elasmobranchs (whale shark *Rhincodon typus*, zebra shark *Stegastoma fasciatum*, great white shark *Carcharodon carcharias*, thorny skate *Amblyraja radiata*) and the holocephalan ghost shark *Callorhinchus milii*. Branch support determined with transfer bootstrap expectation [79] is indicated. While elasmobranchs have three tenascins (TNC, TNR and TNX), the ghost shark has only two (TNC and TNR)

Since the description of the ghost shark tenascins, the genomes of several sharks and rays (Subclass Elasmobranchii) have been sequenced and assembled. These include, amongst several others, the whale shark *Rhincodon typus* [85, 86], the great white shark *Carcharodon carcharias* [87], the thorny skate *Amblyraja radiata* [88], the small-spotted catshark *Scyliorhinus canicula* [89], and the zebra shark *Stegostoma fasciatum* [90]. The present study identified tenascins in each of these species. The tenascins of the whale shark are typical of tenascins found in this subclass of cartilaginous fishes and are described as an exemplar here. A gene on *R. typus* chromosome 28 encodes a protein identified by BLASTP and domain analysis as TNC-like (XP_048469310). This TNC is predicted to have 15 EGF-like domains, 16 FN3 domains and a carboxy-terminal FReD; an IEG motif is found in the carboxy-terminal-most FN3 domain (Fig. 5A). Whale shark TNR (XP_048458101), identified by the same criteria, is predicted to have three EGF-like domains and nine FN3 domains, none of which have recognizable integrin-binding motifs. Finally, the whale shark, unlike the ghost shark, has a TNX (XP_048472635, XP_020378480) which is predicted to have 11 EGF-like domains, 27 FN3 domains and a carboxy-terminal FReD (Fig. 5A). The tenascins found in the other cartilaginous fishes listed above, as well as their accession numbers and genetic neighbors (see below), are summarized in Supplementary TableS1 (Tenascins from representatives of the Class Chondrichthyes). Unlike the ghost shark, the elasmobranch species each have three tenascins corresponding (by protein-based criteria) to TNC, TNR and TNX. These identifications were supported by phylogenetic tree construction based on the FReD amino acid sequences (Fig. 5B).

### Tenascins of lobe-finned fishes

Lobe-finned fishes share a common bony lobe-finned ancestor with tetrapods [91]. The four tenascins of the West Indian Ocean coelacanth *Latimeria chalumnae* were described previously from partial predicted sequences [7]. By criteria of BLASTP and protein domain analysis, this lobed-finned fish encodes TNC (XP_064420300), TNR (XP_064415793), TNW (XP_064416217) and TNX (XP_064408017). These tenascins are summarized in Supplementary Table S2 (Tenascins from representatives of the Class Sarcopterygii).

The genome of the West African lungfish *Protopterus annectens* is also now assembled [92]. Its TNC (XP_043914544) has 18 EGF-like domains, 15 FN3 domains (with IDG and RGD motifs found in the third FN3 domain), and a carboxy-terminal FReD. The TNR (XP_043941895) of the lungfish is typical in having four EGF-like domains and nine FN3 domains. No TNW was found from the *P. annectens* genome, but this lungfish does encode a TNX (XP_043939105) (Supplementary Table S2, Tenascins from representatives of the Class Sarcopterygii).

### Tenascins of non-teleost actinopterygian fishes

The non-teleost clades of ray-finned bony fishes include the bichirs (Order Polypteriformes), paddlefish and sturgeons (Order Acipenseriformes). These ray-finned fishes diverged before the additional or lineage-specific WGDs that occurred in teleosts, though the paddlefish and sturgeons have undergone independent WGDs [93]. In common with the coelacanth (see above), the Senegal (or gray) bichir (*Polypterus senegalus*) encodes four tenascins, corresponding to TNC, TNR, TNW and TNX. Accession numbers and features of these tenascins are summarized in Supplementary Table S3 (Tenascins from non-teleost species of the Class Actinopterygii). These tenascins share the basic domain organization of the orthologous tenascins of tetrapods, though the TNC of the Senegal bichir does not contain an identifiable integrin binding motif.

The American paddlefish *Polyodon spathula* has undergone a single WGD independently from the WGD of teleosts [93]. It has seven tenascins that are summarized in Supplementary Table S3 (Tenascins from non-teleost species of the Class Actinopterygii): a single TNC and two each of TNR, TNW and TNX. The single TNC has 17 EGF-like domains, 14 FN3 domains and a carboxy-terminal FReD. Like the TNC from the Senegal bichir, the paddlefish TNC lacks identifiable integrin-binding motifs. One TNW is encoded on chromosome 14 (TNW14) and one is encoded on chromosome 18 (TNW18). TNW14 has 18 FN3 domains, with an IDG motif in the exposed loop between β strands F and G in 11 of those domains. TNW18 is similar, with IDG motifs in 14 of its 19 FN3 domains. The FReD amino acid sequences of TNW14 and TNW18 are identical. Both predicted TNX proteins have six FN3 domains with an IDG motif in the first FN3 domain.

Studies of the sterlet sturgeon *Acipenser ruthenus* genome have revealed a WGD in the sturgeon lineage during the Jurassic period followed by rediploidization that involved a loss of whole chromosomes [94]. The sterlet, like the paddlefish, has seven tenascins, but while TNC, TNR and TNW are duplicated, there is only a single TNX. These tenascins are summarized in Supplementary Table S3(Tenascins from non-teleost species of the Class Actinopterygii). The TNC encoded on chromosome 30 (TNC30) and the TNC encoded on chromosome 16 (TNC16) are proteins nearly identical in domain organization and integrin-binding motifs. Similarly, the two predicted TNR proteins (TNR10 and TNR11) are also nearly identical. Whereas one TNW (TNW10) has numerous IDG motifs in its 11 FN3 domains (like the paddlefish TNWs above), TNW11 has only five FN3 domains and a single IDG motif.

### The tenascins of the zebrafish *Danio rerio*

**Fig. 6:**
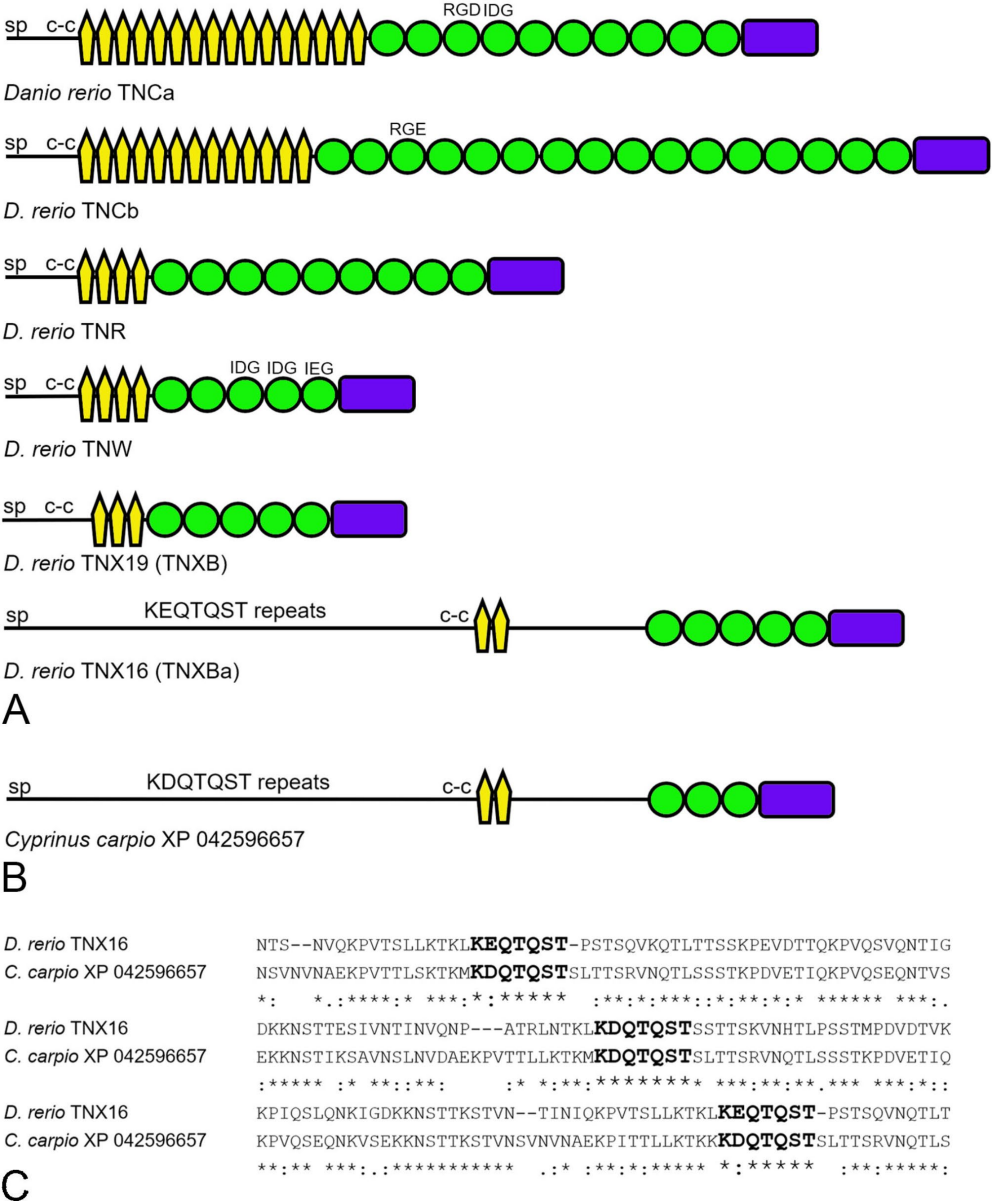
**A**. Schematic models of the six tenascin paralogs of the zebrafish *Danio rerio* (TNCa, XP_021332057; TNCb, NP_001299845; TNR, NP_919364; TNW, CAA04755; TNX19, XP_021323869; TNX16, XP_021322558). The N-terminal region of TNX16 includes many copies of an approximately 60-amino acid long repeat (with the sequence KEQTQST most highly conserved) between the signal peptide and the coiled-coil-forming heptad repeats. **B**. The domain architecture of a TNX from the common carp *Cyprinus carpio*. **C**. Sequence alignment of 180 amino acids from the repeat region of the *C. carpio* TNX with the same region in *D*. *rerio* TNX16. The highly conserved amino acids selected to represent the repeat are shown in bold

Previous studies identified four tenascins in the zebrafish *Danio rerio*: TNC, TNR, TNW and TNX [12], but the same study revealed two TNCs (TNCa and TNCb) in the pufferfishes *Tetraodon negroviridis* and *Takifugu rubripes*. Reanalysis of the latest reference assembly of the zebrafish genome now reveals six tenascins: two TNCs (TNCa and TNCb), TNR, TNW, and two TNXs. The names TNCa and TNCb are already adopted by the databases (e.g., GeneID). In keeping with the nomenclature used in this paper to describe duplicated paralogs, the TNX encoded by a gene on chromosome 16 will be referred to as TNX16, and the one encoded by a gene on chromosome 19 as TNX19. These tenascins are summarized in Supplementary Table S4(Tenascins from representatives of the Infraclass Teleostei) and their domain organizations are shown schematically in Fig. 6A. TNCa and TNCb contain RGD and RGE motifs in the third FN3 domain, respectively, and TNCa has an IDG motif in its fourth FN3 domain. The TNR and TNW have the expected domain architectures. Whereas TNX19 (referred to as TNXB in GeneID) is a “classic” TNX with three EGF-like domains, five FN3 domains and a FReD, TNX16 (a.k.a. TNXBa) is unusual. Between the signal peptide and the coiled-coil region is a large stretch that includes 14 copies of an approximately 70 amino-acid long repeat that includes a motif K(E/D)QTQST. Carboxy-terminal to the coiled-coil region are two EGF-like domains, a stretch of consensus disorder, five FN3 domains and a FReD. The tenascins of other teleosts were examined for similar glutamine-rich repeats. The repeats are present in one TNX from all cyprinids (carps and minnows of the family Cyprinidae, to which *D. rerio* belongs) examined, including the common carp *Cyprinus carpio* (XP_042596657; identified in NCBI as “mucin-3A-like isoform”; Fig. 6B), also the Prussian carp *Carassius gibelio* (XP_052435238; identified as “mucin-3A isoform”), the cavefish *Sinocyclocheilus rhinocerous* (XP_016411794; “tenascin-like”), the tiger barb *Puntigrus tetrazona* (XP_043117471; “tenascin”), and the fathead minnow *Pimephales promelas* (XP_039530853; “tenascin”). Similar repeats were found in the TNX of only one non-cyprinid species, the razorback sucker *Xyrauchen texanus* (XP_051999443; identified as “mucin-4”), which belongs to the family Catostomidae. Other proteins with these repeats were not identified with BlastP. An alignment between three of these repeats from *D. rerio* and *Cyprinus carpio* shows conservation of the K(E/D)QTQST motifs (Fig. 6C).

### Tenascins of other teleosts

To include an example of tenascins from a non-cyprinid teleost in this study, tenascins were identified in the southern bluefin tuna *Thunnus maccoyii*. Similar to *D. rerio*, this tuna encodes six tenascins: two TNCs, TNR, TNW, and two TNXs (Supplementary Table S4, Tenascins from representatives of the Infraclass Teleostei). TNC9, but not TNC19, has a tripeptide integrin-binding motif in an exposed loop between β strands F and G of the third FN3 domain. The TNR and TNW from tuna are remarkably similar to these tenascins from other ray-finned fishes, including the presence of multiple copies of potential integrin-binding motifs in TNW. TNX10 is predicted as a small protein, with a single EGF-like domain and four FN3 domains, while TNX15 is considerably larger, with 10 EGF-like domains and five FN3 domains. The TNXs from the bluefin tuna do not contain the repeated motifs found in the cyprinid teleosts.

In addition to the shared teleost WGD, salmonids underwent an independent WGD event approximately 80 million years ago [95]. This event was so recent in evolutionary terms that rediploidization in salmon and trout is still in progress. Not surprisingly, therefore, the chinook salmon *Oncorhynchus tshawytscha* has 11 tenascins (Supplementary Table S4, Tenascins from representatives of the Infraclass Teleostei). Four are TNCs that are named here for the linkage group in which the encoding genes are located: TNC4, TNC12, TNC20 and TNC33. TNC4 and TNC12 have nearly identical domain organization, and both have potential integrin-binding motifs in their third FN3 domain. There are also two copies each of genes encoding TNR and TNW, and three encoding TNX. TNR10 and TNR28 have identical domain organization (four EGF-like domains and nine FN3 domains). TNW10 and TNW28 are similar, but TNW10 contains eight FN3 domains (six with IDG motifs) and TNW28 has 11 FN3 domains (seven with IDG motifs). Two TNXs are named TNX13 and TNX31, and a third found on an unplaced scaffold is named here TNXb. TNX13 has 11 EGF-like domains and six FN3 domains, with a large segment of consensus disorder between the second and third FN3 domain. TNX31 has a more typical tenascin domain architecture, and TNXb has the same domain architecture as TNX10 from the southern bluefin tuna.

### Tenascins of *Xenopus laevis* and additional tetrapods

The tenascin paralogs in mammals, birds and the anuran western clawed frog *Xenopus tropicalis* were described previously, though the predicted sequences from *X. tropicalis* were incomplete [12]. Just as the salmonid genome provides insight into recent rediploidization, the genome of the tetraploid African clawed frog *Xenopus laevis* can be compared with that of the congeneric (but diploid) *X. tropicalis* to learn about retention of duplicated genes. The WGD that resulted in tetraploidy in *X. laevis* is estimated to have occurred as recently as 17–18 million years ago [63]. The homologous chromosomes that resulted from this duplication are numbered and followed by L for long or S for short, as one of each chromosome pair has lost approximately 17% of its sequence [63]. As introduced above, *X. tropicalis* is now confirmed to encode four tenascins: TNC, TNR, TNW and TNX (Supplementary Table S5, Tenascins from *Xenopus tropicalis* and *X. laevis*). In contrast, *X. laevis* encodes two copies of genes encoding TNC, TNR and TNX and a single gene encoding TNW (Supplementary Table S5, Tenascins from *Xenopus tropicalis* and *X. laevis*).

TNC from *X. tropicalis* and TNC8L and TNC8S from *X. laevis* have the same domain architecture: 14 EGF-like domains, 11 FN3 domains and a FReD. The third FN3 domain of each of these TNCs has RGD/RGE and IDG/IEG motifs in exposed loops between β strands. At the amino acid level, *X. tropicalis* TNC and *X. laevis* TNC8L are 93.1% identical, and TNC8L and TNC8S are 94.6% identical. *X. tropicalis* TNR has four EGF-like domains and nine FN3 domains, whereas *X. laevis* TNR4L and TNR4S both have five EGF-like domains and nine FN3 domains. TNR4L is unusual for having an RGD motif in the exposed loop between β strands F and G. There is also one additional EGF-like domain found in TNW4L compared with *X. tropicalis* TNW, but both have five FN3 domains that contain IDG and RGD motifs. The TNX from *X. tropicalis* and both TNXs from *X. laevis* all have 24 EGF-like domains, but the numbers of FN3 domains are more variable: 12 in *X. tropicalis* TNX, 15 in *X. laevis* TNX8L, and 11 in *X. laevis* TNX8S.

To gain a better understanding of potential diversity among the tenascins of tetrapods the predicted tenascins of the green anole *Anolis carolinensis* and the Iberian ribbed newt *Pleurodeles waltl* were identified. As in other amniotes, the green anole encodes four tenascins. The TNC (XP_016852949), TNR (XP_008107154) and TNW (XP_008107169) are remarkably similar to their orthologs in birds, including potential integrin-binding motifs in both TNC and TNW. However, green anole TNX (as assembled from three adjacent predicted proteins: XP_016847338, XP_016846340 and XP_016847354) has a 131 amino acids-long proline-rich region between the second and third FN3 domains that is not observed in TNX from birds and mammals. This proline-rich region was identified as a series of “proline-rich extensin signatures” by PRINTS (PRO1217) [96]. Extensins are carbohydrate-binding components of the plant cell wall [97]. Though polypeptide regions are commonly present between the second and third FN3 domains of TNX in other species, these are typically identified as “consensus disorder” and do not share homology with the extensins.

The genome of the newt *P. waltl* was identified to encode four predicted tenascins: TNC (KAJ1145804.1), TNR (KAJ1173138.1), TNW (KAJ1173132.1) and TNX (KAJ1134636.1). All four predicted tenascins include signal peptides, coiled-coil regions, EGF-like domains, FN3 domains, and a carboxy-terminal FReD. The TNC is predicted to have 13 EGF-like domains and eight FN3 domains, the latter with an IDG motif in the third FN3 domain in an exposed loop. The TNR, like TNR from other tetrapods, has four EGF-like domains and nine FN3 domains, none of which have recognizable integrin-binding motifs. The predicted TNW of *P. waltl* is a small protein, with only four EGF-like domains and four FN3 domains. There is an RGE motif in an exposed loop in the second FN3 domain, and an IDG motif in an exposed loop in the fourth FN3 domain. The predicted newt TNX is larger, with 22 EGF-like domains and 22 FN3 domains. There is an 887 amino acid-long sequence rich in prolines and lysine residues but without recognizable features between the second and third FN3 domains, and none of the TNX FN3 domains have predicted integrin-recognition motifs.

The numbers of each tenascin paralog found in the species of jawed vertebrates examined above are summarized in Supplementary Table S6 (Gnathostome tenascins: Summary).

### The tenascin assembly domain

**Fig. 7:**
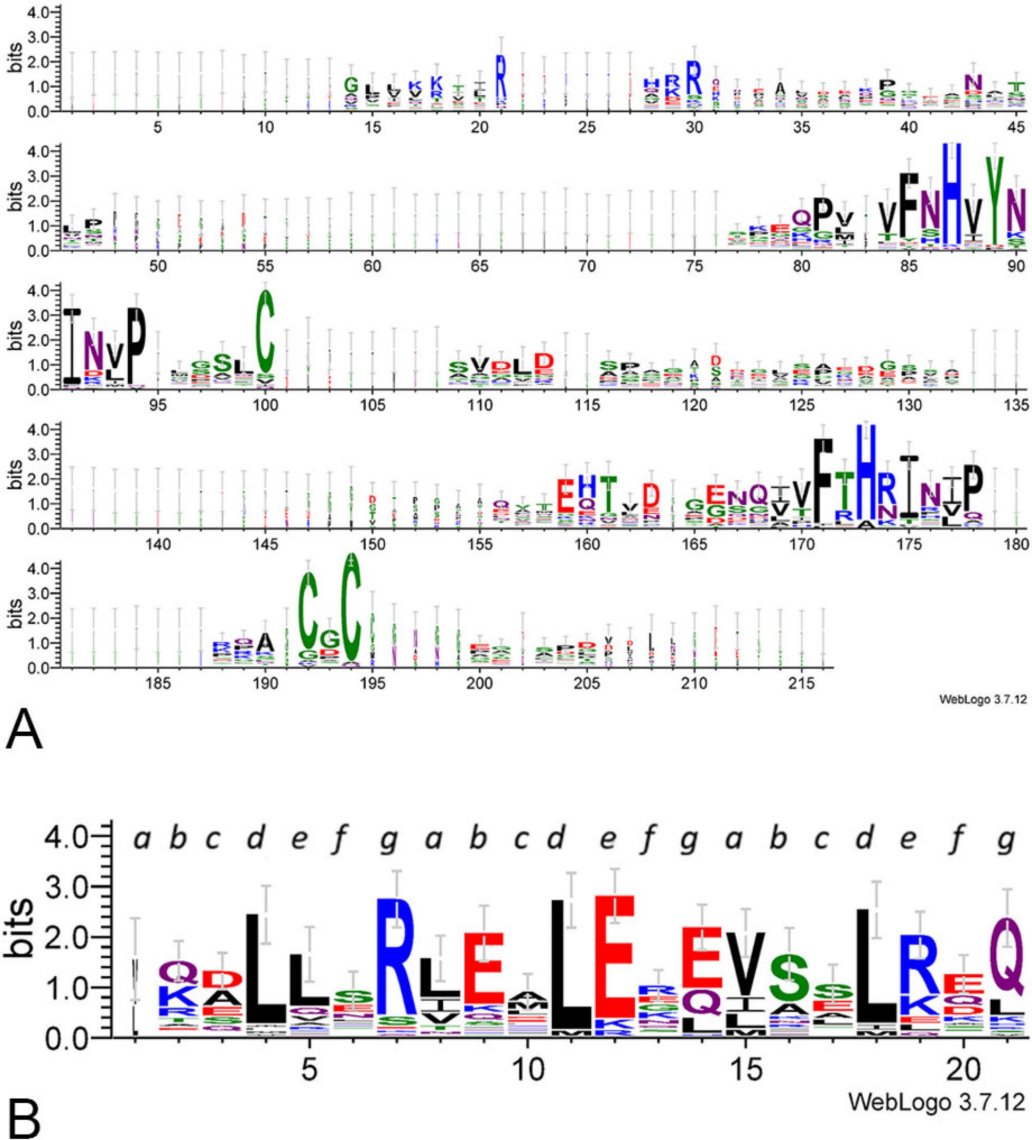
Identification of conserved residues within the tenascin assembly region. **A**. WebLogo for the complete region between the signal peptide and the heptad repeats. **B**. WebLogo for the heptad repeat region. In each case, datasets of 39 representative tenascin sequences corresponding to the above regions were aligned in MUSCLE 3.8.31 and sequence Logos prepared from the MUSCLE outputs in WebLogo 3. Amino acids are colored according to chemistry: polar, green; neutral, purple; basic, blue; acidic, red; hydrophobic, black. Lettering in **B** refers to positions *a* to *g* in the heptad repeats. The dataset is available in Supplementary File S1

As described in the Introduction, both the short heptad-repeat regions and cysteine residues within the assembly domain of TNC (Fig. 1C) participate in assembly of TNC hexamers through a two-step process. Cysteine residues corresponding to C-64, C-111, C-113, C-140, C-146, and C-147 of human TNC were previously reported to be conserved in human, mouse, and chicken TNC [14, 15]. We examined sequence conservation of the assembly region of all tenascins from representative taxa from tunicates to humans by the Sequence Logo method, that provides a more in-depth view of amino acid conservation than a conventional multiple sequence alignment, and also by multiple sequence alignments of individual tenascin family members to capture paralog-related distinctions (Supplementary File S1). The Logo showed that the assembly regions are for the most part poorly conserved and variable in length. However, three cysteines corresponding to C-64, C-111 and C-113 of TNC are highly, although not universally, conserved (Fig. 7A). The Logo also revealed two previously unappreciated well-conserved, related short motifs: vFnHvYnINvP and vFtHrIniP (where capitals indicate highly conserved amino acids and lower case indicates the most frequent amino acid for that position) within the assembly region (Fig. 7A).

The multiple sequence alignments of individual tenascin family members confirmed high conservation of the three cysteines in TNC orthologs (Supplementary Fig. S1A) and in TNR orthologs (Supplementary Fig. S1B), although the assembly region of TNRs shows less overall conservation than those of the TNCs (Supplementary Fig. S1C). The assembly domains of TNW orthologs include no conserved cysteine residues (not shown). However, three cysteines in the tenascin assembly domain of coelacanth TNW align with C-64, C-111 and C-113 of chicken and human TNC (Supplementary Fig. S1D). The putative tenascin assembly domains of TNX align very poorly due to variable lengths and very little sequence conservation (Supplementary Fig. S1E). Conserved cysteines are present in TNX of bony fishes, sharks, and rays, but only one cysteine is present in the short TNX assembly domains of tetrapods *G. gallus* and *X. tropicalis*. The predicted tenascins of *Ciona intestinalis* and *Phalusia mammallata* also include cysteines in their tenascin assembly regions that align with cysteines C-64, C-111 and C-113 in the assembly regions of chicken and human TNC (Supplementary Fig. S1F).

The heptad-repeat region of TNC is typically around 24 amino acids long [14]. A sequence analysis of the heptad-repeat regions of TNC, TNR, TNW, and TNX from tetrapods, as well as *C. intestinalis* tenascin and *B. floridae* tenascin, identified a mean length of 27 amino acids with a well-conserved central motif of RLxxLE. The majority of these were predicted to assemble as trimers; however, *C. intestinalis* and *B. floridae* tenascins were predicted to assemble as dimers [98]. With benefit of a more comprehensive dataset of representative tenascin sequences (39 sequences from 15 species), we identified additional examples of the tenascin heptad-repeats and examined the conservation of the heptad-repeat regions by Sequence Logo. This analysis demonstrated conservation of hydrophobic residues in line with the heptad *a* and *d* positions and clarifies the central conserved motif as RhExLE (where h is a hydrophobic residue) (Fig. 7B).

### Tenascin phylogeny

Phylogenetic trees were assembled based on the amino acid sequences of the FReDs of representative tenascins (Supplementary File S2, S3). The amino acids that comprise these domains are readily identifiable and can be confidently aligned. In addition, these domains each have the same or nearly the same number of amino acids across a broad range of tenascins. The FReD region was used previously to construct tenascin phylogenetic trees [7, 12, 41].

**Fig. 8:**
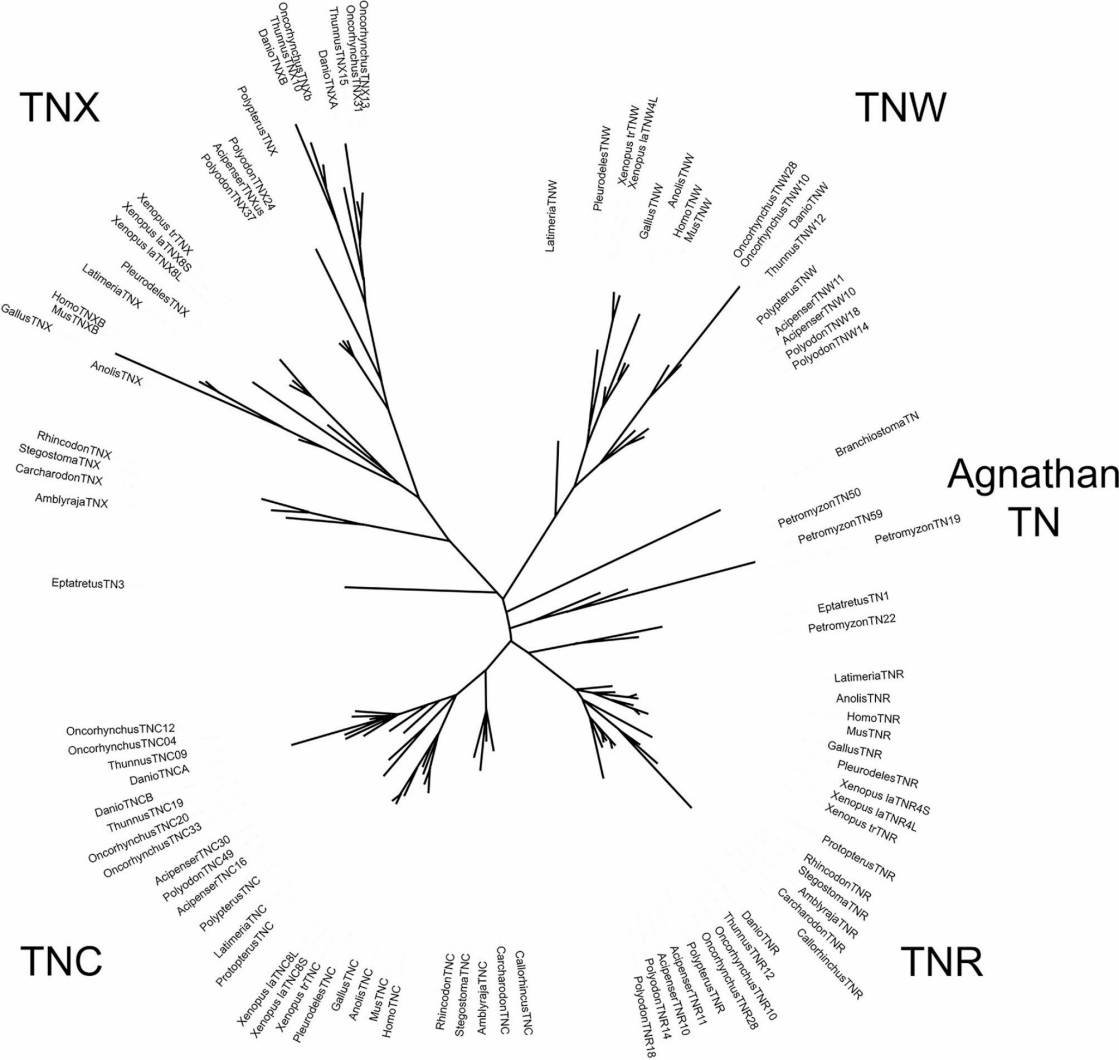
Unrooted phylogenetic tree based on the FReD amino acid sequence from 99 tenascins from 22 chordates. Branch support can be found in Supplementary Fig. S3 and the dataset is available in Supplementary File S2

The phylogenetic tree including tunicate sequences yielded six clades, corresponding to tunicate tenascins, agnathan tenascins, TNX, TNR, TNC and TNW (Supplementary Fig. S2), with the tunicate tenascins rooted near the center of the tree. A phylogenetic tree constructed without the tunicate sequences, which displayed very long branch lengths, is similar (Fig. 8; a version of this phylogenetic tree with branch support values is found in Supplementary Fig. S3). Interestingly, the tenascin of the lancelet *B. floridae* segregated to the agnathan clade together with most of the tenascins from hagfish and sea lampreys. However, the inshore hagfish’s TN1 and the sea lamprey’s TN22, (which share genetic neighbors with TNR in jawed vertebrates, see below), segregate with the TNR clade.

To examine the relationships between the different tenascins that arose through WGDs within the gnathostome lineage, separate phylogenetic trees were also constructed for each of the TNC, TNR, TNW and TNX clades (Supplementary Fig. S4). For example, the chinook salmon’s TNC04 and TNC12 appear to have duplicated recently from a TNC similar to *D. rerio* TNCa, whereas the chinook salmon’s TNC20 and TNC33 appear to have arisen from the duplication of a TNC similar to *D. rerio* TNCb. For the most part, the branchpoints in these trees are consistent with what is generally accepted as the evolutionary history of jawed vertebrates (e.g., note the coelacanth TNW clusters with the TNW of tetrapods, and the separate clades formed by the TNWs of teleosts and ancestral bony fishes). The scales of the trees are also informative: the amino acid sequences of FReDs from TNRs are more conserved (shorter branch lengths) than those of the other tenascins, whereas the TNX FReD sequences have diverged the most (note the scale bars indicating substitution per site). This is also evident in Fig. 8, where the same scale is applied to each clade.

### Conservation of synteny: analysis of tenascin gene neighbors

**Fig. 9:**
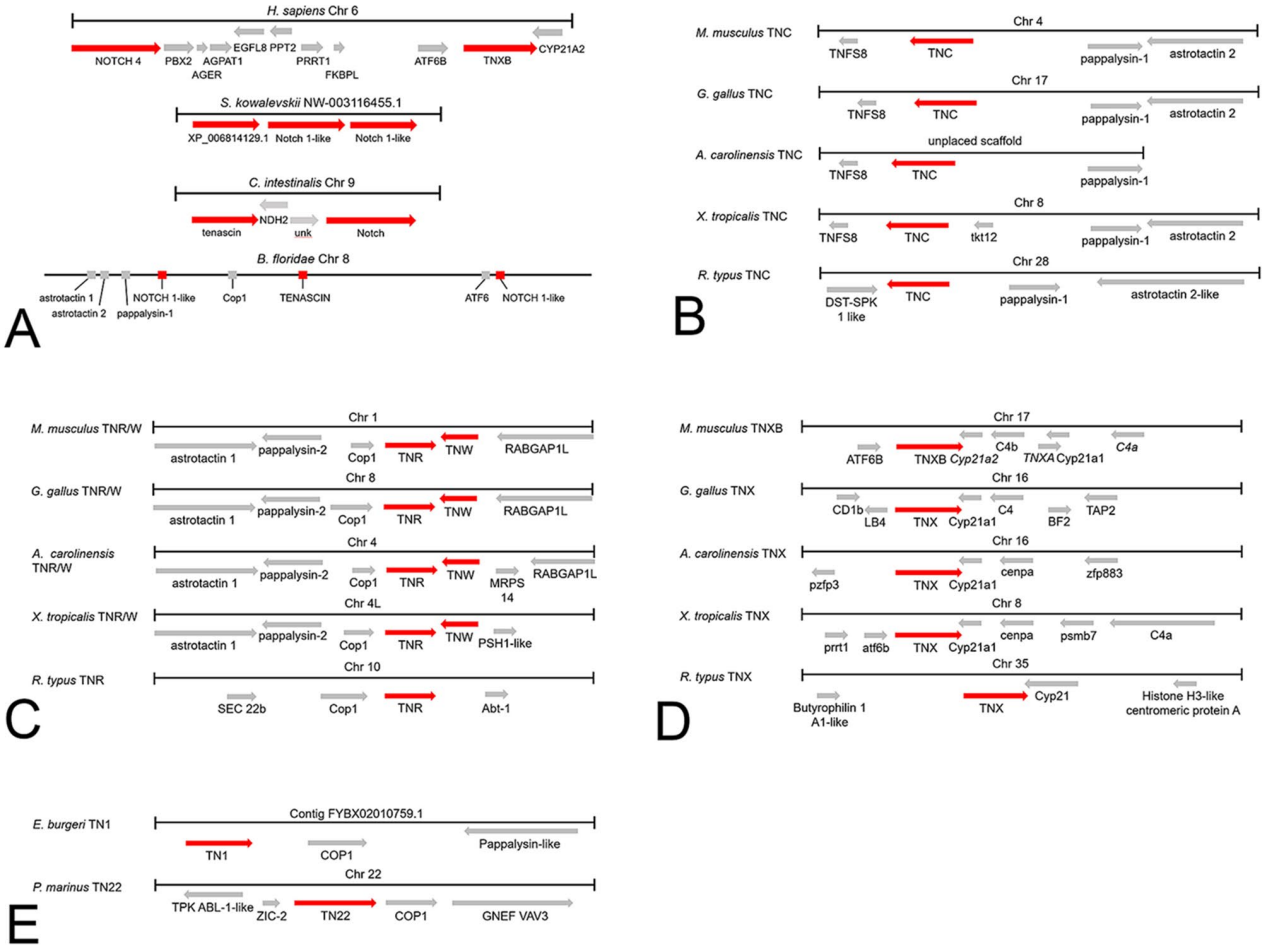
Conservation of synteny for *tenascin* genes. **A**. Schematic representation of the arrangement of genes around *TNXB* and *Notch 4* in *Homo sapiens* (chromosome 6); the gene encoding tenascin-like XP_006814129.1 and *Notch 1-like* in the hemichordate *Saccoglossus kowalevskii* (scaffold NW-003116455.1); *tenascin* and *Notch* in the vase tunicate *Ciona intestinalis* (chromosome 9), and around *tenascin* and *Notch* on chromosome 8 of the Florida lancelet, *Branchiostoma floridae*. **B**. *TNC* and its neighbors in the house mouse *Mus musculus* (chromosome 4), the chicken *Gallus gallus* (chromosome 17), the green anole *Anolis carolinensis* (unplaced scaffold), the western clawed frog *Xenopus tropicalis* (chromosome 8) and the whale shark *R. typus* (chromosome 28). **C**. *TNR* and *TNW* (also known as *TNN*) are found adjacent to each other in *M. musculus* (chromosome 1), *G. gallus* (chromosome 8), *A. carolinensis* (chromosome 4), *X. tropicalis* (chromosome 4). The whale shark R. typus only has *TNR* (chromosome 10). **D**. *TNX* (named *TNXB* in species with the pseudogene *TNXA*) is adjacent to *Cyp21*-related genes or pseudogenes (pseudogenes are indicated with italics in the figure) in *M. musculus* (chromosome 17), *G. gallus* (chromosome 16), *A. carolinensis* (chromosome 16), *X. tropicalis* (chromosome 8) and *R. typus* (chromosome 35). **E**. *TN1* from the inshore hagfish *Eptatretus burgeri* (contig FYBX02010759.1) and *TN22* from the sea lamprey *Petromyzon marinus* (chromosome 22) are adjacent to *Cop1*. The gene names of the neighboring genes are those given in the respective genome projects for each species

As a separate route to analyze the relationships of tenascin family members and any lineage-specific duplications, we examined the conservation of genes that are linked to the loci of tenascin-encoding genes (i.e., seeking evidence for conservation of synteny) in representative species from hemichordates to tetrapods. A prior analysis of the chromosomal loci of human tenascin genes showed that *TNR* and *TNW* are adjacent on chromosome 1, and that *TNC*,*TNR*,* TNW* and *TNN/TNX* are all encoded from within large regions of paralogy within the human genome, that are known as paralogous MHC loci and which also include paralogous *Notch* genes [12]. Specifically, *Notch1* (XP_011517019) is found on human chromosome 9 near *TNC*,*Notch2* (CAH70182) is on human chromosome 1 as are *TNR* and *TNW*, and *Notch4* (AQY77346) is found on human chromosome 6, close to *TNX* (Fig. 9A). We found that the synteny with *Notch* is relevant to the origin of *tenascin* genes, since the *S. kowalevskii* gene encoding tenascin-like XP_006814129 (*LOC102802137*) is on the same genomic scaffold as, and adjacent to, two genes encoding predicted Notch-like proteins (Fig. 9A). Similarly, the encoding genes of the *C. intestinalis* and *B. floridae* tenascins (as is the tenascin gene of *B. lanceolatum*) are also syntenic with *Notch* loci (Fig. 9A). In view of this conservation, we conducted a deeper analysis to identify additional conserved syntenic genes in representative species that encode tenascins. The results are described first for each tenascin family member of gnathostomes.

### TNC

In the genomes of mouse *Mus musculus*, chicken *G. gallus* and green anole *A. carolinensis*, each *TNC* is flanked on one side by a gene that encodes tumor necrosis factor superfamily protein 8 (*TNSF8*), and on the other side by genes encoding pappalysin-1 (*PAPPA1*) and astrotactin 2 (*ASTN2*) (Fig. 9B). In *X. tropicalis* (the *P. waltl* genome is still under assembly) an additional gene is found between *TNC* and *PAPPA1.*

In the *D. rerio* genome (reference assembly GRCz11), unusually, two TNC paralogs are located on the same chromosome, chromosome 5: *TNCa* (at bp 5,707,991–5,830,978) and *TNCb* (at bp 28,561,006–28,690,286). *TNCa* is located between the gene encoding TNF receptor-associated factor 2 (*TRAF2A*) (LOC100005446) and a gene encoding an uncharacterized protein (XP_009299436), and *TNCb* is located between a second *TRAF2A* gene and a gene encoding beta-1,3-galactosyltransferase 8 gene (*b3galt8*) (Supplementary Table S4, Tenascins from representatives of the Infraclass Teleostei). *Notch-1b* (GeneID 794892; chromosome 5 at bp 65,676,304 − 65,782,882) is syntenic with *TNCa* and *TNCb*. The Southern bluefin tuna, examined as an additional teleost fish, also encodes two *TNC* paralogs, but these are found on different chromosomes and hence are designated *TNC9* and *TNC19*. *TNC9*, like *D. rerio TNCb*, is located between *b3galt8* and *TRAF2A*, whereas *TNC19* is located between the phosphoinositide-3-kinase regulatory subunit 1 gene (*Pik3R1*) and *TRAF2A* (Supplementary Table S4, Tenascins from representatives of the Infraclass Teleostei). In contrast, the chinook salmon genome encodes four *TNC* paralogs on different chromosomes, of which three (*TNC4*, *TNC20* and *TNC33)* share chromosomal neighbors. All three genes are adjacent to *TRAF2A*; *TNC20* and *TNC33* are also adjacent to *Pik3R1*, whereas *TNC4*, like *TNC9* of tuna and zebrafish *TNCb*, is adjacent to *b3galt8*. The fourth *TNC* paralog, TNC12, has distinct gene neighbors: dual specificity testis-specific protein kinase 1 (*TESK1*) and *Rab-6* (Supplementary Table S4, Tenascins from representatives of the Infraclass Teleostei).

Considering non-teleost ray-finned fish, in the Senegalese bichir *Polypterus senegalus*, *TNC* is located on chromosome 13 with some distinct genetic neighbors: kelch-like protein 9 (*LOC120542393*) and dipeptidyl-peptidase 7 (*dpp7*) to one side, and *TRAF2A* and NADPH dependent diflavin oxidoreductase 1 (*ndor1*) to the other (Supplementary Table S3, Tenascins from non-teleost species of the Class Actinopterygii). Genes encoding pappalysin and astrotactin homologs are not found on chromosome 13, but instead are located adjacent to each other on chromosomes 9 and 14. On chromosome 9, two genes encoding zinc finger-containing proteins (*LOC120535130* and *znf618*) are adjacent to *PAPPA1* and *ASTN2* instead of *TNC*. In the American paddlefish genome, gene neighbors of *TNC* resemble those of the bichir rather than those of other jawed vertebrates, as *TNC* is located on micro chromosome 49 adjacent to the gene encoding Kelch-like protein 9 (*LOC121306676*) (Supplementary Table S3, Tenascins from non-teleost species of the Class Actinopterygii). The sterlet sturgeon genome contains two *TNC* paralogs, located on chromosome 30 (*TNC30*) and on chromosome 16 (*TNC16*); both genes are flanked by *TESK* and *TRAF2A* paralogs (Supplementary Table S3, Tenascins from non-teleost species of the Class Actinopterygii).

In the *L. chalumnae* (coelacanth) genome, *TNC* (Gene ID: 102366554) is located on chromosome 10, with *PAPP1*, *ASTN2* and *TRIM32* adjacent to one side. In the West African lungfish, *TNC* is located on chromosome 16 and is flanked on one side by *ASTN2* and *PAPPA1*, and on the other side by *TNSF8* and *TNSF15* (Supplementary Table S2, Tenascins from representatives of the Class Sarcopterygii). This mirrors the conservation of synteny seen for *TNC* in most tetrapods. The lungfish *NOTCH1* (Gene ID: 122790804) is also found on chromosome 16.

In the ghost shark, *C. milli*, *TNC* is adjacent to the genes *PAPPA1* (ENSCMIT00000019498) and *ASTN2* (ENSCMIT00000019419) on one side, and the gene encoding *TESK2* (ENSCMIT00000020409) on the other. In the whale shark, *R. typus*, *TNC* on chromosome 28 is also flanked by equivalent genes (Fig. 9B). A *Notch-1-like* gene is also syntenic at LOC109923636.

### TNR and TNW

In each of the tetrapod genomes examined, *TNR* and *TNW* are located immediately adjacent to each other on opposite DNA strands (Fig. 9C). Adjacent to *TNR* is the gene encoding Cop1 E3 ubiquitin ligase (*Cop1*), which in turn is adjacent to *PAPPA2*, a paralog of *PAPPA1* that has conserved synteny with *TNC* (Fig. 9B). This arrangement also includes nearby genes encoding astrotactin (*ASTN*) paralogs. Gene neighbors of the *TNR* and *TNW* paralogs of *X. tropicalis* (Fig. 9C) and *X. laevis* are also well conserved. In *X. laevis*, *TNR4S*, (which lacks a neighboring *TNW* paralog), is flanked by the same genes that flank *TNR* in *X. tropicalis* and *TNW* is simply missing from the region between *MRPS14* and KIAA0040.

Considering teleost fish, in the *D. rerio* genome, *TNR* and TNW are located adjacent to each other on chromosome 2 and are also flanked by *Cop1* and *MRPS14*, respectively, with *PAPPA2* and *ASTN1* conserved adjacent to *Cop1* (Supplementary Table S4,Tenascins from representatives of the Infraclass Teleostei). In tuna, *TNR* and *TNW* are adjacent to each other on chromosome 12, with *MRPS12* adjacent to *TNW* (Supplementary Table S4,Tenascins from representatives of the Infraclass Teleostei). *Cop1*,* PAPPA2* and *ASTN1*, are adjacent to one another and also located on chromosome 12, but nearly ten million base pairs distant, suggesting a lineage or species-specific DNA insertion event. Unlike *D. rerio* or *T. maccoyii*, the chinook salmon encodes two TNRs and two TNWs. *TNR10* and *TNW10* are adjacent as are *TNR28* and *TNW28* (Supplementary Table S4,Tenascins from representatives of the Infraclass Teleostei). The two tenascin genes are flanked by paralogous *Cop1* and *MRPS14* genes.

In the Senegalese bichir, *TNR* and *TNW* are located together on chromosome 14 and are flanked on one side by *ASTN1*,* PAPPA2*, and *Cop1* and by *MRPS14* on the other. In the American paddlefish genome adjacent *TNR*,* TNW* pairs are located on chromosomes 14 and 18, respectively, in each case between *Cop1* and *MRPS14*. *Notch* genes are syntenic with both pairs of genes (*LOC121326829*, Chr14: 16,185 − 76,351; *LOC121331096*, Chr18: 36,244,947 − 36,292,336). In the sterlet sturgeon genome, *TNR* and *TNW* are again adjacent to each other and flanked by *Cop1* and *MRPS14*. These results are summarized in Supplementary Table S3 (Tenascins from non-teleost species of the Class Actinopterygii).

In the coelacanth *L. chalumnae*, *TNR* (Gene ID: 102366423), is at 27,867,547 to 27,970,357 on chromosome 6, adjacent to *Cop1* (Gene ID: 102363850) and with *PAPP2* and *ASTN1* adjacent on the other side (Supplementary Fig. S5). *TNR* is adjacent to *Cop1* (ENSLACT00000012739) on Scaffold JH126898. *TNW* of *L. chalumnae*, unlike in tetrapod genomes, is not adjacent to *TNR*, yet is also found on chromosome 6 (Gene ID: 102351342; range 101643103 to 101684025) adjacent to the gene encoding *MRPS14* (Gene ID: 102347992). Further examination of chromosome 6 showed that *ATF6* (Gene ID: 102350356) and a *Notch-like 1* (Gene ID: 102352538) are also encoded on this chromosome (Supplementary Fig. S5). *MRPS14* is also adjacent to *TNW* in the ray-finned fishes, green anole and *X. tropicalis* (e.g., see Fig. 9C*)*. In the West African lungfish genome, *TNR* is on chromosome 10, with coiled-coil and C2 domain containing 1B (*CC2D1B*), *Cop1*,* PAPPA2* and *ASTN1* located to one side. As described above, *TNW* is not present. These results are summarized in Supplementary Table S2 (Tenascins from representatives of the Class Sarcopterygii).

In the ghost shark *Callorhincus milli TNR* is located on chromosome 10, where it is adjacent to *Cop1* (XP_048458163) and a gene encoding RAB GTPase activating protein 1 (*RABGAP1*). *Cop1* also is found adjacent to TNR in the great white shark, whale shark, thorny skate, small-spotted cat shark and zebra shark (Supplementary Table S1, Tenascins from representatives of the Class Chondrichthyes) (Fig. 9C). A gene encoding a Notch-like protein is syntenic (*LOC109914551*) in *R. typus*.

### TNX

In the mouse, chicken, green anole and *X. tropicalis*,* TNX* is located adjacent to the gene encoding steroid 21-hydroxylase (*Cyp21*) (Fig. 9D). In an ancestral mammal, *Cyp21* underwent a tandem duplication together with a fragment of *TNX* (Miller, 2021). In humans and rodents, the partially duplicated *TNX* pseudogene is named *TNXA*, and the protein-encoding gene is *TNXB* [40]. As this duplication is unique to a subset of mammals, and since genes arising through WGDs in other animals are often named A or B to distinguish between paralogs, the term *TNXB* is used here only to describe human and murine *TNX*. In *D. rerio*, the single *TNX* (*TNX16*) is also located adjacent to *Cyp21*. In tuna, *TNX* is encoded on chromosome 10 (*TNX10*), between *Cyp21* (LOC121905446) and complement *C4b*. A second *TNX* paralog, *TNX15*, is located on chromosome 15, flanked by *MHC Class I-related* (*LOC121913711*) and *trnap-ugg*, encoding a tRNA. The Chinook salmon genome encodes four TNX homologs, two of which share chromosomal neighbors. Both *TNX13* and *TNX31* are located adjacent to the gene encoding activating transcription factor 6 beta (*ATF6b*) and proteasome 20 S subunit beta 10 (*psmb10*) homologs; *psmb10* adjacent to *TNX13* is a pseudogene. *TNXb*, like *TNX10* from tuna and *TNX16* from zebrafish, is located between *Cyp21* and complement *C4b*. These results are summarized in Supplementary Table S4 (Tenascins from representatives of the Infraclass Teleostei).

Considering non-teleost ray-finned fish, in the Senegal bichir, *TNX* is located on chromosome 11 adjacent to *C4b*; no *Cyp21* homologue was identified. The American paddlefish genome contains two *TNX* homologs, one being located on chromosome 24 (*TNX24*) and the other on chromosome 37 (*TNX37*). *TNX24* is located between complement *C4b* and the gene encoding proteasome subunit beta type-7-like protein (*LOC121299059*). *Cyp21* (*LOC121299051*) is located on chromosome 24 more remotely (between base pairs 22,562,648 − 22,577,034; ~65,000 base pairs away). *TNX37* is located adjacent to the gene encoding proteasome subunit beta type-7-like (*LOC121304324*) and is flanked on the other side by the gene encoding 1-acyl-sn-glycerol-3-phosphate acyltransferase alpha-like (*LOC121304322*). No *Cyp21* paralog was identified on chromosome 37. The sturgeon genome encodes a single *TNX* (LOC117434005); the unplaced scaffold sequence also includes complement *C4b* (LOC117968147). These results are summarized in Supplementary Table S3 (Tenascins from non-teleost species of the Class Actinopterygii).

*TNX* of *L. chalumnae* is on chromosome 26 (Gene ID 102345859), adjacent to *ATF6b* (ENSLACT00000008721). *Cyp21* (ENSLACT00000015610) is also on chromosome 26. *TNX* of West African lungfish is located on chromosome 9 near *ATF6b* (*LOC122811369*) and complement *C4B*. *Notch4* is located more distantly on chromosome 9 (Supplementary Table S2, Tenascins from representatives of the Class Sarcopterygii).

In the whale shark, *R. typus*, *TNX* (*LOC125486978*) is partially overlapped by *Cyp21* (*LOC109925585*) on chromosome 35 (Fig. 9D), and a Notch-1-like homolog is encoded syntenically at *LOC125486947* on chromosome 35. *Cyp21* is found adjacent to *TNX* in each of the Elasmobranch genomes examined (Supplementary Table S1, Tenascins from representatives of the Class Chondrichthyes).

### Tenascin gene loci in agnathans and early-diverging chordates

Having identified conserved gene neighbors of tenascin-encoding genes in gnathostomes, we next examined the loci of tenascin genes in available agnathan and early-diverging chordate genomes; most of these species encode smaller numbers of tenascins than tetrapods (see above).

The syntenic relationships documented for *TNR* and *TNW* were also evident for *TN* loci of agnathans. The *E. burgeri* (inshore hagfish) *TN1* gene lies adjacent to *Cop1* (ENSEBUT00000006005) and *PAPPA2* (ENSEBUT00000026049). The *E. burgeri* TN2 is adjacent to a midasin-like gene (ENSEBUT00000004546) and a gene encoding solute carrier family 25 member 23a (ENSEBUT00000007822). The *E. burgeri* TN3 is a partial sequence and its genetic neighbors are unknown. In the sea lamprey *P. marinus* genome, *TN22* is encoded on chromosome 22 adjacent to *Cop1* (XP_032814602; Fig. 9F) and near one of two *PAPPA1-like* (*LOC116944890*) and *ASTN2-like* loci (*LOC116944888*). *Cyp21* is also encoded on chromosome 22 (*LOC116945069*). On chromosome 50, *TN50* and the other *ASTN2-like* (*LOC116953184*) homolog flank *PAPPA1-like* (*LOC116953181*). Two genes encoding Notch homologs have been identified in *P. marinus*: one is syntenic with *TN22* (*LOC116945057*) and the other is syntenic with *TN59* (*LOC116955417*).

Our further analysis of *B. floridae* chromosome 8, around the region of the single tenascin gene (*LOC118421312*), identified, in addition to the two genes encoding Notch1-like proteins (*LOC118420675*; 7,427,128–7,450,515) and (*LOC118421069*; 21,410,209 − 21,446,369) (Fig. 9A), that *LOC118421312* is overlapped almost entirely by the gene encoding zinc finger CCCH-type with G patch domain-containing protein (*LOC118421319).* It is flanked by genes encoding protein C10-like (*LOC118421351*) and sialidase-1-like (*LOC118421336*). The *B. floridae Cop1* gene (*LOC118422087*) is also found in chromosome 8 (11,553,417 − 11,582,228), as are *PAPPA1* (*LOC118421143*; 5,344,330–5,381,218) and the genes encoding astrotactin-1 (*LOC118421619*; 4,368,973–4,379,924) and astrotactin-2 (*LOC118421140*; 4,368,973–4,379,924) (Fig. 9A). A homolog of Cyp21 was not identified in *B. floridae*, which is consistent with the observations of others [99]. The genes encoding Notch-1-like proteins, Cop1, astrotactin-1, and pappalysin-2 are all found on chromosome 9 in *B. lanceolatum*, the same chromosome on which the tenascin (XP_066294307.1) gene of this related cephalochordate is found. In the tunicate, *C. intestinalis*, although a Notch-encoding gene (NCBI gene ID 723806; protein NP_001037825) and the single tenascin-encoding gene (*LOC100178031*) are both located on chromosome 9 and are separated by only two intervening genes (Fig. 9A), the single *Atf6* homolog of *Ciona* (referred to as *ATFD*) is encoded on chromosome 13 and homologs of *PAPPA1*, *Cop1* and *ASTN1* were not found in the *C. intestinalis* genome.

In tetrapods, *ATF6b* is frequently near or adjacent to *TNX* (e.g., Fig. 9D). It is also found near or adjacent to *TNX* in elasmobranchs. For example, in *Carcharodon carcharias* (great white shark) *ATF6b* (encoding predicted protein XP_041068174.1) is found on chromosome 19 (104,267,401 − 104,376,450), as is *TNX* (104,694,487 − 104,919,411). In *B. floridae* a gene named “*ATF6b*” is found on chromosome 8 (*LOC118420980*; 21,283,498 − 21,296,967), the same chromosome with the gene encoding tenascin (Fig. 9A). However, in each of the gnathostome genomes examined here, a gene encoding an ATF6 paralog, *ATF6a*, shares synteny with *TNR*. For example, *ATF6a* and *TNR* are both found on chromosome 16 in *C. carcharias*, and *ATF6a* and *TNR/TNW* are found on chromosome 4 in *X. tropicalis*. Phylogenetic analysis of ATF6a and ATF6b amino acid sequences together with representative ATF6 sequences from invertebrates places the “ATF6b” sequence from *B. floridae* in the invertebrate ATF6 clade, and not with ATF6a or ATF6b (Supplementary Fig. S5). This is consistent with the finding that *B. floridae* has a single *ATF6* gene.

## Discussion

**Fig. 10:**
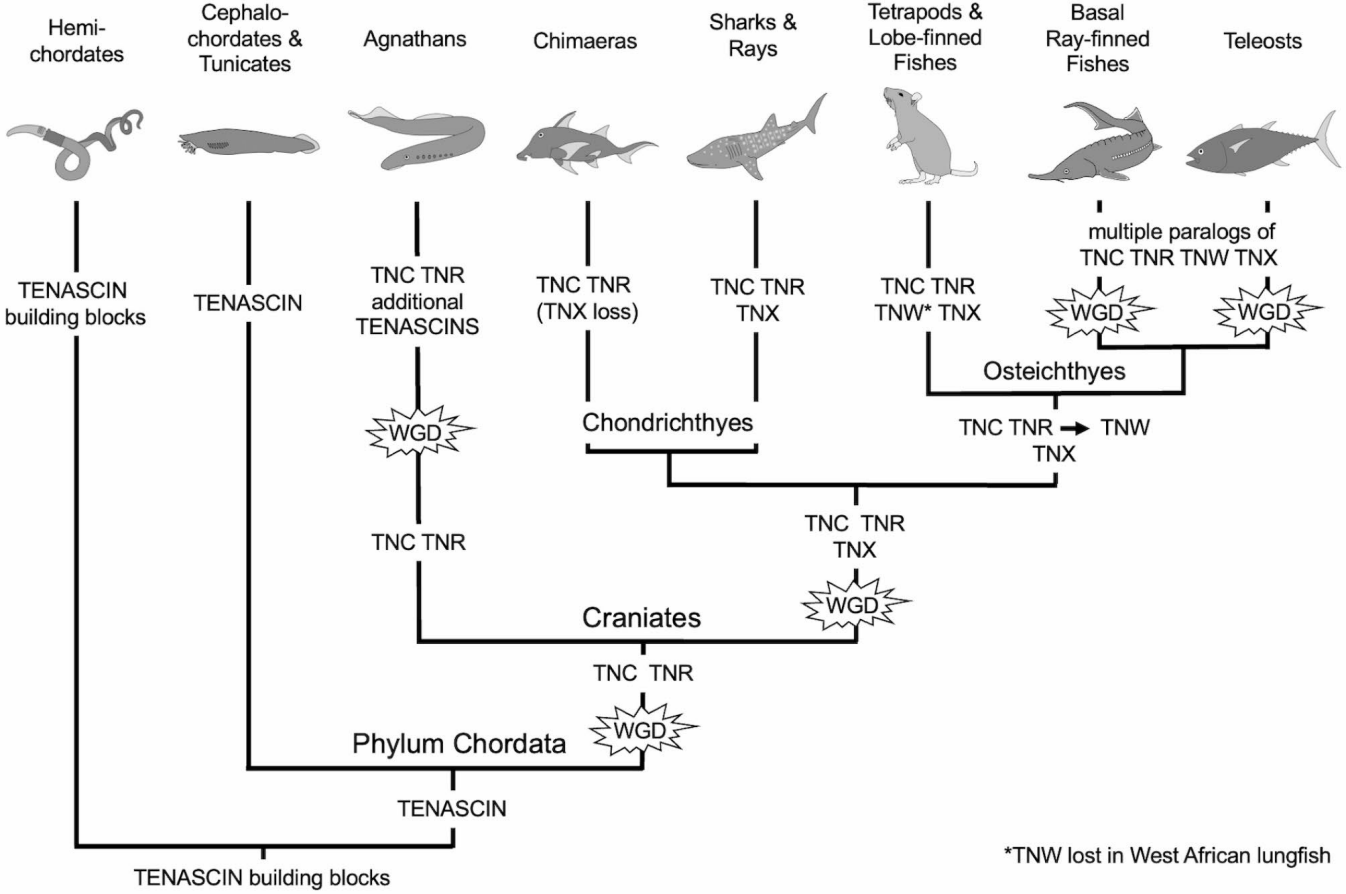
A model of tenascin evolution. A single tenascin gene is proposed to have appeared early in the chordate lineage, perhaps from building blocks found in the genome of a common ancestor with hemichordates, whereas *TNC-like* and *TNR-like* paralogs are proposed to have originated following a whole genome duplication (WGD) event early in the evolution of vertebrates. Additional WGD events lead to the appearance of additional *tenascin (TNC-* and *TNR-like)* paralogs in agnathans. The appearance of *TNX* in a common ancestor of cartilaginous and bony fishes is attributed to duplication of *TNR* through the second WGD in vertebrates. *TNX* has been lost in chimaeras (holocephalans) but is found in all other fishes and tetrapods. A local duplication of *TNR* is proposed to have led to the appearance of *TNW* (also known as *TNN*) in an ancestor of lobe-fined and ray-finned fishes, which roughly coincides with the appearance of bone. Thus, four tenascin paralogs are found in sarcopterygians (lobe-finned fishes and tetrapods), with the exceptions of the West African lungfish, and the tetraploid clawed frog *Xenopus laevis*. Additional WGD events in the ray-finned fishes results in the appearance of numerous paralogs of *TNC*, *TNR*, *TNW* and *TNX*. See text for further details

This analysis has examined tenascin evolution according to protein domain architecture, sequence conservation, phylogenetic relationships as identified from the domain, and genetic relationships according to conservation of synteny. Together, the data enable a new perspective on the evolutionary debut of tenascin and a new model for the evolution of the tenascin gene family (Fig. 10).

### Origin of tenascins

One or more tenascin genes were found in each of the chordate genomes examined here, but tenascin genes were not identified in the closest relatives of the chordates: the hemichordates and echinoderms, nor in a xenacoelomorph. Nevertheless, the basic building blocks of tenascins are widespread in metazoans. Tenascin-type EGF-like domains are found in teneurins (the “ten” in teneurins is named from this domain) [100], and teneurins are found in all metazoans except sponges, cnidarians and placozoa [101]. These domains are also found in reelin, expressed in the nervous systems of chordates, molluscs and arthropods [102]. FN3 domains are present in both intracellular and extracellular proteins found in all metazoans, as well as in viruses, archaea and bacteria [103]. FReDs, too, are found in multiple proteins in all metazoan phyla [104]. Here, we report that the hemichordate *S. kowalevskii* encodes a predicted protein, XP_006814129, with a tenascin-type EGF-like domain and FN3 domains, one of which has a KGD motif that aligns with the integrin-binding RGD motif of chicken TNC, and also encodes a protein with a tenascin-like FReD (XP_002731388). It is tempting to speculate that genetic recombination between these loci could have led to a monomeric, tenascin-like ancestor. With insertion of an assembly domain or heptad repeats, the tenascin of a chordate ancestor may have resembled the tenascins of modern tunicates and cephalochordates. These findings place the debut of tenascin in a common ancestor of the earliest-diverging chordates, i.e., the cephalochordates and tunicates.

The proposal that *S. kowalevskii* XP_006814129 (Fig. 2) might represent a modern equivalent of a “tenascin precursor” is strengthened by consideration of the genomic location of its encoding gene. The respective encoding gene is found adjacent to two *Notch-1-*like genes (Fig. 9A). This syntenic relationship is also seen in tunicates and cephalochordates: the single tenascin gene from the vase tunicate *C. intestinalis* is found near *Notch* on chromosome 9 (Fig. 9A), and two *Notch-1-like* genes and the single tenascin-encoding gene are found on chromosome 8 of the Florida lancelet *B. floridae* (Fig. 9A). Our analysis of genomic loci and conservation of synteny in chordates (discussed in more detail below) strongly support that *TNC*, *TNR* and *TNX* loci evolved from an ancestral locus syntenic to *Notch*, as evidenced by the conservation of *Notch* loci on the same chromosomes as *tenascins* in all the phyla examined. *Notch* loci were previously reported to be syntenic with the four *tenascin* genes of tetrapods [12].

Chromosome 8 of *B. floridae* not only contains the tenascin gene and two *Notch* genes, but it also has genes encoding homologs of Cop1, pappalysin, two astrotactin paralogs, and ATF6 (Fig. 9A). In the genomes of vertebrates, *Cop1* is consistently found near *TNR*, genes encoding pappalysin and astrotactin paralogs are found near both *TNR* and *TNC*, and genes encoding the paralogs ATF6a and ATF6b are syntenic with *TNR* and *TNX*, respectively. These observations are consistent with a model of tenascin evolution in vertebrates from duplications of a single gene locus resembling that found in *B. floridae*. Although *C. intestinalis tenascin* is also syntenic with *Notch* (Fig. 9A; the encoded Notch protein has highest homology to Notch-1), homologs of *PAPPA1*, *Cop1* and *ASTN1* are not encoded, and *ATF6D* is found on a different chromosome. This is consistent with the known rapid evolution of the *C. intestinalis* genome that has involved gene losses and rearrangements [105].

According to domain organization and protein sequence identity criteria, tenascins with homologous features to TNC and TNR appear to have been the first to evolve as paralogs, since these are the only types of tenascins recognizable in the predicted proteomes of agnathans, the FReD domains of these tenascins segregate near the TNR clade when analyzed phylogenetically and, similarly, the TNC and TNR FReD domain clades are also closely related (Fig. 8). It must be noted that the phylogenetic trees here are based on a single domain, the FReD, and so do not take into account the complexity of tenascins as multi-domain proteins. However, the domain architecture of *P. marinus* TN22, in particular, is TNR-like. In most bony fishes and tetrapods, TNR has four EGF-like domains and nine FN3 domains, whereas sea lamprey TN22 has four EGF-like domains and 11 FN3 domains. Unlike most TNRs, both TN1 and TN22 contain putative integrin binding motifs, suggesting that they may play different or additional roles than TNRs in jawed vertebrates.

Independent evidence for a homologous relationship of agnathan TNs with TNC and TNR was provided by the analysis of conservation of synteny, that showed *TNR* and *TNC* of gnathostomes to share *pappalysin* and *astrotactin* genes as close chromosomal neighbors. For example, *TN1* from the hagfish and *TN22* from the sea lamprey are next to *Cop1*, which is also adjacent to *TNR* from most elasmobranchs, bony fishes and tetrapods (i.e., the jawed vertebrates) (Fig. 9C, F). *Pappalysin-*like genes are found near hagfish *TN1* as well as near *TNR* from jawed vertebrates, and *pappalysin-*like genes are also found adjacent to *TNC* in jawed vertebrates. Interestingly, a *pappalysin-*like gene is also found adjacent to the gene encoding the huge, predicted tenascin (TN50) from the sea lamprey. However, care should be taken when interpreting these observations, as the tenascin genes of agnathans cannot be viewed as strict orthologs of the four tenascins of vertebrates.

The proposed origin of agnathan TNC-like and TNR-like homologs through a large-scale genomic duplication event in an early vertebrate is in agreement with recent sequencing of a hagfish (*Eptatretus atami*) genome that supports an auto-tetraploidization in the chordate lineage predating the cyclostome-gnathostome divergence [106]. Three predicted tenascins were identified in the inshore hagfish *E. burgeri* and four in the sea lamprey *Petromyzon marinus*, likely originating from a further genome duplication event in the agnathan lineage [106].

Some of the agnathan *tenascin* loci have syntenic relationships also apparent for the *tenascin* loci in gnathostomes. For example, *TN1* from the hagfish and *TN22* from the sea lamprey are next to *Cop1*, which is also adjacent to *TNR* from most elasmobranchs, bony fishes and tetrapods (i.e., the jawed vertebrates) (Fig. 9C, F). *Pappalysin-*like genes are found near hagfish *TN1* as well as near *TNR* from jawed vertebrates, and *pappalysin-*like genes are also found adjacent to *TNC* in jawed vertebrates. Interestingly, a *pappalysin-*like gene is also found adjacent to the gene encoding the huge, predicted tenascin (TN50) from the sea lamprey. However, care should be taken when interpreting these observations, as additional phylogenetic analysis of agnathan tenascin sequences is necessary before identifying strict gnathostome orthologs.

The domain architecture of *P. marinus* TN22, in particular, is TNR-like. In most bony fishes and tetrapods, TNR has four EGF-like domains and nine FN3 domains, whereas sea lamprey TN22 has four EGF-like domains and 11 FN3 domains. However, unlike most TNRs, both TN1 and TN22 contain putative integrin binding motifs, suggesting that they may play different or additional roles than TNRs in jawed vertebrates. In the chicken and mouse, *TNR* is almost exclusively expressed in the nervous system. When gene expression data become available for agnathans, it will be interesting to see if hagfish TN1 and sea lamprey TN22 are also expressed in the nervous system.

### Origin of tenascin-X

The domain organization of TNX is the most variable of the tenascin proteins. For example, in the chicken, TNX has a single EGF-like domain, whereas human TNX has 18 EGF-like domains (Fig. 1A, B). The numbers of FN3 domains are also very variable, ranging from four in TNX10 of the Southern bluefin tuna to 33 in the TNX of the small-spotted cat shark *Scyliorhinus canicula* (XP_038671561). Not only are there different numbers of domains in TNX from different species, but novel proline-rich repeats are often found between the FN3 domains, or, in the case of cyprinids, between the signal peptide and the heptad repeats (Fig. 6A, B). The functions of these repeats are unknown. It is possible that these repeats may improve TNX’s binding to ECM. The proline-rich repeats found in TNX from the anole, for example, are most similar to repeats found in plant cell walls that bind to carbohydrates [107].

The FReD domains of TNX proteins also show clear sequence divergence and greater inter-species diversity than the equivalent domains of TNC and TNR (evident as longer branch lengths) (Fig. 8, Supplementary Fig. S4). In general, there is good evidence that duplicated gene pairs often evolve subsequently at different rates (asymmetric evolution), which is thought to reflect reduced or altered selection pressures on one of the duplicated genes, whilst the other retains ancestral functions [108, 109].

Despite the distinct sequence and domain features of TNX, its encoding gene also has a conserved syntenic relationship with *Notch* genes in tetrapods [12]. A deep synteny of *TNX* with *TNC/TNR* is indicated by the commonalties of syntenic relationships between *TNR* of agnathans and most *TNX* described here. *TNX* is commonly found near or adjacent to *ATF6b*, and *TNR* shares synteny with *ATF6a*. There are also commonalties between T*NX* loci and *TN* loci of agnathans. *Cyp21*, the steroid-21-hydroxylase gene is found adjacent to *TNX* in almost all genomes examined and is also found on sea lamprey chromosome 22 (XM_032959174; chr 22 856000–844000), where 34 predicted genes separate it from the *TNR*-like *TN22* (chr 22 27850000–26770000). These data indicate an origin of *TNX* through duplication of a “*TNR* homolog” locus through the secondary WGD duplication event in the early gnathostome lineage, followed by genetic rearrangements leading to a mostly distinct profile of conserved gene neighbors for *TNX* versus *TNR* in tetrapods. As noted above, care should be exercised when interpreting syntenic relationships in the sea lamprey without additional phylogenetic analysis.

Previously, we showed that the cartilaginous chimaera *Callorhinchus milii* (class Chondrichthyes, subclass Holocephali), encodes only TNC and TNR, while bony fishes and tetrapods encode TNC, TNR, TNW and TNX [7]. With benefit of the sequencing and assembly of additional genomes from cartilaginous fishes, it is now clear that sharks and rays (class Chondrichthyes, subclass Elasmobranchii) encode three tenascins: TNC, TNR and TNX. This further supports our proposal that TNX evolved from the second round of genome duplication in a gnathostome ancestor, and was retained in Elasmobranchs and secondarily lost in the chimera lineage. Supporting the gene loss hypothesis is the observation that *C. milii* encodes hundreds to thousands fewer genes and gene families than the elasmobranchs [110]. *TNX* is usually found adjacent to *Cyp21* and near *C4*; in *R. typus* (whale shark) *TNX* and *Cyp21b* overlap on opposite strands of DNA, just as *TNXB* overlaps *Cyp21a2* in both mouse and human. However, neither *Cyp21* nor *C4* homologs were identified in *C. milii*, which is consistent with the gene loss hypothesis. A corollary of this model of the origin of *TNX* is that the WGD event would also have involved an equivalent duplication of the “*TNC* homolog” locus, which apparently resulted in conservation of one of the genes (i.e., *TNC*) and loss of the other paralogous locus.

### Origin of tenascin-W

In this study, *TNW* was not identified in cartilaginous fishes but is present in the genomes of all bony fishes and tetrapods examined except for the West African lungfish *Protopterus annectens*. In tetrapods and ray-finned fishes, *TNW* is found adjacent to *TNR* (Fig. 9C), which in turn is adjacent to *Cop1*. In *P. annectens*, *TNR* is adjacent to *CC2D1B* and *Cop1*, but instead of being next to *TNW* it is flanked by *TUT4*. The encoding of TNW in the opposite orientation adjacent to *TNR* in tetrapods and ray-finned fishes has been taken to indicate that *TNW* originated through an inverted tandem duplication of *TNR* in an ancestor of lobe-finned and ray-finned bony fishes [7, 43]. However, with the benefit of chromosome assignments for the tenascin genes of *L. chalumnae*, it appears that the adjacent inverted location of *TNR* and *TNW* is likely to be secondary. In the coelacanth (Supplementary Fig. S5) *TNR* and *TNW* are located on the same chromosome but widely separated. The *TNR* locus shows conservation of synteny of gene neighbors with the *TNR/TNW* loci of teleost and tetrapods species, and *ATF6* and a *Notch-like* are also on the same chromosome, indicating a conservation of syntenic genes with *Branchiostoma tenascin*. It is possible therefore that *TNW* arose through a local duplication of *TNR*, with subsequent loss of intervening genes in non-teleost species of the Class Actinopterygii, teleosts and tetrapods. One cannot rule out the possibility that the local duplication took place earlier in the gnathostome lineage and that *TNW* was subsequently lost in cartilaginous fishes [111]. A further possibility is that *TNW* could represent the ‘lost tenascin’ from the 2R WGD in the vertebrate lineage, that became transposed onto the same chromosome as *TNR*. Though strongly suggested by their location on the same chromosome and supported by domain architecture criteria (Fig. 1), our hypothesis on the origins of *TNW* through local duplication of *TNR* is not supported by phylogenetic analysis of the FReD alone, which fails to show TNW and TNR as sister clades. The FReD domain of TNW, like that of TNX, appears to have diverged rapidly and shows greater divergence and inter-species diversity than the equivalent domains of TNC and TNR (Fig. 8). As noted above, duplicated genes often evolve subsequently at different rates.

In tetrapods, the most prominent site of *TNW* expression is in bone [35]. The absence of *TNW* in *P. annectens* may be a case of the exception proving the rule: while Devonian lungfish had heavy, bony skeletons [112], the wispy limbs of *P. annectens* are supported internally by cartilaginous tubes, and the fins are supported by repeating cylinders of cartilage [113, 114]. Thus, the appearance and disappearance of *TNW* in tetrapods is tied to the presence and loss of bone, respectively.

### Tenascin assembly domain

Overall, tenascin assembly regions are poorly conserved with the exceptions of cysteine residues previously identified to be important in trimer stabilization and cross-linking. These are present in all TNC orthologs examined, the tenascins of tunicates and in TNR orthologs (the latter form trimers but not hexamers) but are curiously absent from the assembly regions of most TNWs, even though murine TNW is known to form hexabrachions [17]. The assembly regions of TNXs have variable numbers of cysteines and are also the most variable in length. Additionally, our sequence analysis identified two related well-conserved motifs, vFnHvYnINvP and vFtHrIniP. The significance of these is unknown, but the residues are predicted by JPred 4 to lie within beta-sheets and so might contribute to the structure of the assembly region. In contrast, the heptad-repeat regions are highly conserved in most tenascins; this would be in line with a conserved function for the assembly of trimeric coiled-coils [14, 98]. In TNW, which, as discussed, is considered to have originated through a duplication of TNR, the cysteines conserved in TNR have not been retained. Thus, oligomerization of TNW must predominantly depend on the heptad-repeats, although it remains unclear how hexabrachions are assembled. The heptad-repeat regions of TNX orthologs tend to be shorter and may lack the capacity to form a stable coiled-coil structure. In combination with the limited conservation of cysteine residues in TNX assembly regions, this may explain why TNX is often detected as monomers.

### Integrin-binding by tenascins

Putative integrin-binding motifs are present in many of the tenascins described here, leading us to suggest that tenascin-integrin interactions are conserved and fundamental to tenascin function. Indeed, in the tenascins of cephalochordates, putative integrin-binding motifs are often found in numerous adjacent FN3 domains (e.g., Fig. 3A). A similar situation is sometimes encountered in vertebrate tenascins (e.g., in the TNW of the American paddlefish, Southern bluefin tuna and Chinook salmon). In ray-finned fishes, integrin-binding motifs are most commonly seen in TNC and TNW homologs, and much less commonly in TNR and TNX. This is consistent with experimental results of tenascin-integrin interactions from tetrapod models, in which TNC and TNW bind to integrins via RGD-like or IDG-like motifs, but TNR and TNX typically do not [24, 115]. We previously observed in tetrapod tenascins that species often have an RGD/KGD/RGE integrin-biding motif in either TNC or TNW, but typically not in both [7]. This study shows that this is also the case in the zebrafish, sterlet sturgeon, Southern bluefin tuna and Chinook salmon: in each of these teleost fishes it is TNC that contains the RGD or RGD-like motif. In cartilaginous fishes, which lack TNW, the IDG/IEG motif is consistently observed in TNC, but not the RGD motif. As the RGD/KGD/RGE motif is commonly present in tenascins from cephalochordate and tunicate species, its absence in tenascins from most cartilaginous fishes suggests that TNC in these species may have fewer (or different) roles than in other chordates. The same might be said for the bichir and American paddlefish, as their TNCs also lack RGD/KGD/RGE motifs.

### Evolutionary model

Taken together, the analyses of phylogenetic relationships and conservation of synteny lead us to propose a new model for the evolution of tenascins (Fig. 10). We propose an origin of tenascins in an ancestor of chordates and that the earliest whole genome duplication in the vertebrate lineage led to the origin of two paralogous genes encoding proteins with characteristics most similar to the TNC and TNR of modern gnathostomes. A secondary genome duplication event in the agnathan lineage led to multiple TNC-like and TNR-like homologs. Because *TNX* is present in both cartilaginous and bony fishes, and because *ATF6a* and *ATF6b* share conserved synteny with *TNR* and *TNX*, we speculate that *TNX* evolved later from a *TN22/TNR*-like gene during the separate second WGD that took place in an ancestral placoderm-like gnathostome. *TNX* is not found in chimaeras but considering the small size of the *C. milii* genome and the absence in *C. milii* of genes that typically neighbor *TNX* in sharks and rays (*Cyp21* and *C4*), it seems likely that *TNX* was lost in the chimaera lineage. The fourth tenascin paralog, *TNW*, is unique to bony fishes and tetrapods and lies adjacent to *TNR* in genomes of non-teleost species of the Class Actinopterygii, teleosts and tetrapods. However, in the coelacanth genome, *TNR* and *TNW* are located more remotely on the same chromosome. The most parsimonious model is that *TNW* first appeared through a local duplication event, and a later chromosomal rearrangement involving loss of intervening genes between *TNR* and *TNW* occurred in a common ancestor of teleosts and tetrapods.

Within teleosts, there are no obvious patterns to the loss and retention of tenascin genes following WGD events and rediploidization (Supplementary Table S6, Gnathostome tenascins: Summary). Independent WGDs in the subclass Chondrostei have resulted in single copies of *TNC* (in the American paddlefish) and *TNX* (in the sterlet sturgeon), while the other tenascin paralogs remain as duplicates. Perhaps a more consistent pattern is the loss of duplicated *TNW* genes. This is observed in teleosts, with the zebrafish and Southern bluefin tuna having single copies of *TNW*, and in amphibians, with *X. laevis*, which underwent a relatively recent WGD event, also having a single *TNW* gene. Even so, one must note duplicated *TNW*s retained in the sturgeon and paddlefish. It will be interesting to study patterns of expression of these paralogs to see if regulatory elements have been conserved or altered during the WGD and rediploidization. In *X. laevis*, where total protein expression for most genes is available, the expression profiles of *TNCS/L* and *TNXS/L* during early development are comparable, but *TNRS* is more highly expressed at developmental stage 41 than *TNRL* [79].

## Conclusions

Tenascin is an ECM glycoprotein that first appeared in the Phylum Chordata and evolved into a family of paralogs following whole genome duplication events and local duplication, with subsequent selective retention of novel genes, and later emerging genes undergoing sequence and apparent functional diversification. The timing of the appearance of specific tenascin types illustrates how an ECM family might contribute to key evolutionary moments: TNC and TNR evolved together with a neural crest-derived endocranium and expanded central nervous system, respectively, and TNW apparently co-evolved with bone.

## Electronic supplementary material

Below is the link to the electronic supplementary material.

Supplementary Material 1

## Data Availability

All data generated or analyzed during this study are included in this published article and its additional information files.

## Abbreviations

ECM: Extracellular matrix
TNC: Tenascin-C
TNR: Tenascin-R
TNW: Tenascin-W
TNX/TNXB: Tenascin-X/tenascin-XB
EGF: Epidermal growth factor
FN3: Fibronectin-type 3
FReD: Fibrinogen-related domain
WGD: Whole genome duplication
