# Supplementary material for: The evolution of tenascins
Source: BMC Ecol Evol. 2024 Sep 14;24:121. doi: 10.1186/s12862-024-02306-2 (PMC11401434; doi:10.1186/s12862-024-02306-2)
Supplement: Supplementary file 1 — Supplementary Material 1 [file 12862_2024_2306_MOESM1_ESM.docx]

**Supplementary File 1**

Adams and Tucker

The tenascin assembly region (from after the SP cleavage site to start of heptad repeats) of representative tenascins in FASTA format used to generate the MSA and Sequence Logo for Figure 7A.

>HsapiensTNC

GVLKKVIRHKRQSGVNATLPEENQPVVFNHVYNIKLPVGSQCSVDLESASGEKDLAPPSEPSESFQEHTVDGENQIVFTHRINIPRRACGCAAAPD

>HsapiensTNR

QLEVTTERVQRQSVEEEGGIANYNTSSKEQPVVFNHVYNINVPLDNLCSSGLEASAEQEVSAEDETLAEYMGQTSDHESQVTFTHRINFPKKACPC

>HsapiensTNXB

GPFSSRSNVTLPAPRPPPQPGGHTVGAGVGSPSSQLYEHTVEGGEKQVVFTHRINLPPSTGCGCPPGTEPPVLASEV

>HsapiensTNW

PPGCSNKEQQVTVSHTYKIDVPKSALVQVDADPQPLSDDGASLLALGEAREEQNIIFRHNIRLQTPQKDCELAGSV

>GgallusTNC

GLIKRIIRQKRETGLNVTLPEDNQPVVFNHVYNIKLPVGSLCSVDLDAASGDADLKAEIEPVKNYEEHTVNEGNQIVFTHRINIPRRACGCAAAPD

>GgallusTNR

RLEVTTEPAERPAVDEEGGLANCSPPVKEQPMVFHHIYNINVPVDSCCSSMLRSSAEEVSSEDDRLAEYTEQTSDSESQVTFTHRINLPKQACKCSTSLP

>GgallusTNW

QLPASENCTSNGQRISFSHSYKIDLPTSSQIKVEADPLQHEGNSGVQLDPGEAEENEEQDIIFRHNIHVHAPKGSCETLAHV

>GgallusTNX

RTGLRTMTPGLPLLLLALALRRAQGNAGASPGPPPVPPELSCGAE

>XtropicalisTNC

GLVRKIIRQKRESGLNISLPEDNQPVVFNHVYNINVPMGSLCSVDLDPTSTKDNELSPEVGKNYQEHNLNGENQIVFTHRINIPRRACGCASAPD

>XtropicalisTNR

QSELTGQTPRLDIQVDKGGNMTDHGIENKQNPMVFNHVYNFNVPLSFPCTLEPETPEDGSWVTQQTENTADAGSQITFTHSIRVPQRLCQCYTTPHAV

>XtropicalisTNW

IKTTQIDNCSSEKGLTFTHVYSSPGEENEKLQTVHSDEQNLVFRHYIHLQNPAADCEKNKL

>XtropicalisTNX

PPSTSTQCPDAGGV

>LchalumnaeTNC

GLIKKLIRHRRASLQPQEENISVTSGEQPVVFNHVYNINLPMGAMCSVDLDAPGGIGIKPQGTMSGQQTEHTMDGDNQIVFTHRINIPRQACGCAAAPD

>LchalumnaeTNR

GLAKADEAKKEAMREQSLLLDHTIPSREQPVVFNHIYNINVPLESLCSMQLESSANQLKDESTTQYLEQTADAENKVTFTHKINIPKHACPCPTSST

>LchalumnaeTNW

KPPEAHNITSTLEHSATFSHIYNIALPKRSFCKVELDSLPLKDEGLGLQAATETAGGDEHIVFRHNIRVQSPQCDCESSAT

>LchalumnaeTNX

EVVTRTFRHRREKGGPLTVFDHVYSIRMPHCQGCLAEVSAPTLEVFEARDEPLPSSSPRQLRYEHTVEGGEEVVFTHRINIPPKACDCIGGLDV

>RtypusTNC

GLIKKIIRQKRDILQPKEENITLPLGTQPINFNHVYNINVPLGSLCSVDLESPDGIARPKADSSDQHIEQTVDGENQIVFTHRINIPRQACGCAAGPD

>RtypusTNR

QLLVSALRQKRDKLSVRGHHMNHTVMIKQQPIVFNHVYNINVPMDSLCSVRLDSSSEQTESMQDEDVSQDSEQNLDSQVAFTHNIHIPKQMCDCPHFNL

>RtypusTNX

GIFVKTIRQRRALLPNATSGRPTTFNHVYNIRLPYCKNCSANIAKPLAHIFEAQDEARDDQSFEHTVDPTNQVVFTHRINIPPQVCGCDE

>PsenegalusTNC

GLIKKVLRHRRESLTAENVTLPDADQPVVFNHVYNINVPQGSLCSVDLDSPGSTDLNPKAASPVQSTEHTLDSENQIVFTHRINIPRQACGCEGLPS

>PsenegalusTNR

GFLVKTTRRRRDTKELEGQNANESLSLKGKAMVFNHVYNINVPLESLCSVSLDSSAGDGVKVGEKEAATQYTEHTMDAENQVIFTHKINIPKQACACPSSMT

>PsenegalusTNW

REGEHGLTFSHVYKIDLPKSSSCNVELDSLPAKDQGSEMVASPEGQLEDNNLVFRHNIRLQTPKCDCEGSDA

>PsenegalusTNX

LETPSLSNAGAKKAPRVMSNKEDPLLDNKKQPSVLSQFTRKSDLGAQPFKEEANTGKLLPQTKTHQNPNQEIPFQNPPQVITLRLSGACTSQVLEGVDGDVNTDFEHVVEPGTPLVITHRITVAPSCSCTEIDI

>Cintestinalis

LRREIRNVTQVGNIGEEEVVFNHIYTINVPDQQQCDCPSLLEGITHTNDPTNPAVNDRFRLFPPPGYVTPSADDVDSKGHPLYAAGFTFRHNINIAESSCPCQTSDL

>Pmammillata

GINQIESRHHVPEPNATLHPTKPLVFNHKYSINIPGNKLCKCPPFMQGGVDGDNRNENSEEFSFETSYDTALQNGSDGFVHVNRGYVTPSQYDVDEHGRPYADLFHFDHRIEIPHKKCLCEGRDL

>Odioica

AKRKLSADSFTSPVVLLETVDQGQIEYKHFYSINYQEDAESARRNCRAGSFSKRKKPQRGARTRRAIKPQKNDVSVTTQGLHFYHKIDLKELTCTCNEEI

>Bfloridae

VPVQGDTHNVANVSKPIISSASVNTPAGGMVFNHVYNINVPPVLCAGADGKVTQTSGTVEVGEGGDGAPFLANTFVPGGGSGGCCNNGGSGSGGDLLNGETGSL

>Bbelcheri

VPVQGDSQSVANASKPIISTASVNTPAGGMVFNHVYNINVPPVLCARGGADGQVTKTSGTVEVGEGGDGAPFLANTFVPGGGSGGCCNNNGGGAGGDLLNGETGSL

>Blanceolatum

VPVSGDSHNVANVSKPIISSASVNTPAGGMVFNHVYNINVPPVLCAGAGGQVTETSGTVEVGEGGEGAPFLANTFVPGGAGGGCCNNNGGSGSSGDLLNGETGSL

>EburgeriTN1

STEEKEYSAVQPSADNMTRTSKEKPLVFNHVYNINLPMGSVCNGGVDEMSESLHDSNGADTFSEHTQDQENQVVFTHRINIPRKACACPSCTDHATE

>EburgeriTN2

RVLRMLQARRSEAHSTGPQTLEAFTDGVSEEPVLFNHIYNINLPAMGSLCDVSVKSGGHASSQGGEFISPSDGESTKRTLDPGNHIVFTHRINIPNLACGCMGSGSGHSPPPPDV

>PmarinusTN50

ASLTVETHRHEPGGLGAAPHDLPYRNLTMGGGSDDQQQQPIVFHHVYNINVPPGSLCAVDLDAGPPDGAGSDSRPVVGSGSGDGDIGGGGESDLGTVTQKTHETGSQVVFTHRISIPKRACGCSTEGLPDL

>PmarinusTN22

LPTAAGGTLEVLTERLRRDVDSSVETSLELGNDDDDNNNNNGGGGGTSSSSSGGGGSQPVHAGGGRHPHVYNHVYTINMPPSALCGAAPQDGDAAADGDSAGAGSAPIDAGGAAGSAMFMEQTEDSDSQIVFTHRISIPRKACGCRGDHAVDHDGDSPL

>PmarinusTN59

RVLERSTHGPHRRLHRRAAAAAPAGRIGKALPPPRPPAGPLAASGGERQVSVQLPADSVAGSGPSPVVFNHVYNINVPLGSMCSVDLQAARGAAGAAAAASITPGHSHVGDGEKSVVFTHRINIPPQACGCGGAGGGGALPN

>PmarinusTN19

ALLPKDARAMPGRPAGDGQQQQQQQQRSHQLEADDAGDGGGSSKRLVFHHVYNINVGDSECAGHGDAAAAAAAAGGGGGGGAARTVEGGGGAVFTHRVVVPSVPCGNHQQQQQQ

>TmaccoyiiTNR12

APSGDSKLVRTTRVKRQTPGGDQPPSASPANQTLWEQPVVFNHVYNINVPLESLCSVDLDASAPPAPGDGSRAELGSTPSVEGPMDPTGPAEYTEQTLDAESQVTFTHRINIPKAACGCPATIT

>TmaccoyiiTNC19

KKILRHRRQTLASPKEHNITLPSADHPVVFNHVYNINVPASALCSVDLDAPENKQLLPNDAAVSPGYRATEHTLDGENQIVFTHRINIPRRACGCSDDMPG

>TmacoyiiTNC09

GLVKKVIRHRREALMPKKTQENLTLPHPDQPVVFNHVYNINVPSTSLCSVDLDSPGDTELKHKSSPVDMQNTEHMEHTVDGDNQIVFTHRINIPKQACGCDNQLPD

>TmacoyiiTNW12

DNHLGPSAPEQGVTFSHVYKIDIPGSTSCKLERLPIQDQAGLQTETTTNGENDITFRHNLMLQMPKCDCEESED

>TmacoyiiTNX15

QQLQVRGQRTPRDTKEDAIKVVISEGCTTHGESSDVSQGGKEIDLVPGSPLVLTHKIKLVPSGSGFGSRPGSCGCEAD

**Supplementary File 2**

Adams and Tucker

The FReDs of representative tenascins in FASTA format used to generate Figure 8.

>GallusTNC

KDCSQALLNGEVTSGLYTIYLNGDRTQPLQVFCDMAEDGGGWIVFLRRQNGKEDFYRNWKNYVAGFGDPKDEFWIGLENLHKISSQGQYELRVDLRDRGETAYAVYDKFSVGDAKTRYRLRVDGYSGTAGDSMTYHNGRSFSTFDKDNDSAITNCALSYKGAFWYKNCHRVNLMGRYGDNNHSQGVNWFHWKGHEYSIQFAEMKLRPS

>GallusTNR

QDCAQHLMNGDTLSGVYTISINGDLSQRVQVFCDMSTDGGGWIVFQRRQNGLTDFFRKWADYRVGFGNLEDEFWLGLDNIHKITSQGRYELRIDMRDGQEAAYAYYDKFSVGDSRSLYKLRIGDYNGTSGDSLTYHQGRPFSTKDRDNDVAVTNCAMSYKGAWWYKNCHRTNLNGKYGESRHSQGINWYHWKGHEFSIPFVEMKMRPY

>GallusTNW

ADCAQMQQNGNTSSGTYTIYLNGDGSRPMQVYCDMTTDGGGWIVFQRRSTGELDFYKRWKNYVEGFGDPTGEFWLGLDQLHNLTSSSPSHYELRVDLRTASESAYAVYDFFQVASSRERYRLSVGNYRGNAGDAMTYHNGWMFTTWDRDNDVALSNCALTHHGAWWYKNCHLANLNGKYGESKHSEGVNWEPWKGHEFSIPFTEMKIRPQ

>GallusTNX

RDCAEEQLNGPGPSREVLIFLGGDRQRPLHVFCDMESNGGGWLVFQRRMDGGTDFWRGWEEYIHGFGNVSGEFWLGNAALHTLTASGPTELRVDLRTPSDSAFARYRDFAVSGPEDNFRLHLGAYSGTAGDALSYHAGSPFSTRDHDPRGRPRPCAVAYTGAWWYRNCHYANLNGRYGVPYDHQGINWYPWKGFEYSIPFTEMKLRPQ

>HomoTNC

KDCSQAMLNGDTTSGLYTIYLNGDKAEALEVFCDMTSDGGGWIVFLRRKNGRENFYQNWKAYAAGFGDRREEFWLGLDNLNKITAQGQYELRVDLRDHGETAFAVYDKFSVGDAKTRYKLKVEGYSGTAGDSMAYHNGRSFSTFDKDTDSAITNCALSYKGAFWYRNCHRVNLMGRYGDNNHSQGVNWFHWKGHEHSIQFAEMKLRPS

>HomoTNR

QDCAQHLMNGDTLSGVYPIFLNGELSQKLQVYCDMTTDGGGWIVFQRRQNGQTDFFRKWADYRVGFGNVEDEFWLGLDNIHRITSQGRYELRVDMRDGQEAAFASYDRFSVEDSRNLYKLRIGSYNGTAGDSLSYHQGRPFSTEDRDNDVAVTNCAMSYKGAWWYKNCHRTNLNGKYGESRHSQGINWYHWKGHEFSIPFVEMKMRPY

>HomoTNW

SDCSQVQQNSNAASGLYTIYLHGDASRPLQVYCDMETDGGGWIVFQRRNTGQLDFFKRWRSYVEGFGDPMKEFWLGLDKLHNLTTGTPARYEVRVDLQTANESAYAIYDFFQVASSKERYKLTVGKYRGTAGDALTYHNGWKFTTFDRDNDIALSNCALTHHGGWWYKNCHLANPNGRYGETKHSEGVNWEPWKGHEFSIPYVELKIRPH

>HomoTNXB

RDCGEEMQNGAGASRTSTIFLNGNRERPLNVFCDMETDGGGWLVFQRRMDGQTDFWRDWEDYAHGFGNISGEFWLGNEALHSLTQAGDYSMRVDLRAGDEAVFAQYDSFHVDSAAEYYRLHLEGYHGTAGDSMSYHSGSVFSARDRDPNSLLISCAVSYRGAWWYRNCHYANLNGLYGSTVDHQGVSWYHWKGFEFSVPFTEMKLRPR

>MusTNC

RDCSQAMLNGDTTSGLYTIYINGDKTQALEVYCDMTSDGGGWIVFLRRKNGREDFYRNWKAYAAGFGDRREEFWLGLDNLSKITAQGQYELRVDLQDHGESAYAVYDRFSVGDAKSRYKLKVEGYSGTAGDSMNYHNGRSFSTYDKDTDSAITNCALSYKGAFWYKNCHRVNLMGRYGDNNHSQGVNWFHWKGHEYSIQFAEMKLRPS

>MusTNR

QDCAQHLMNGDTLSGVYTIFLNGELSHKLQVYCDMTTDGGGWIVFQRRQNGQTDFFRKWADYRVGFGNLEDEFWLGLDNIHRITAQGRYELRVDMRDGQEAVFAYYDKFAVEDSRSLYKIRIGSYNGTAGDSLSYHQGRPFSTEDRDNDVAVTNCAMSYKGAWWYKNCHRTNLNGKYGESRHSQGINWYHWKGHEFSIPFVEMKMRPY

>MusTNW

SDCSQVQQNTNAASGLYTIYLNGDASRPMQVYCDMDTDGGGWIVFQRRNTGQLDFFKRWRSYVEGFGDPMKEFWLGLDKLHNLTTGTTTRYEVRADLQTFNESAYAVYDFFQVASSKERYKLSVGKYRGTAGDALTYHNGWKFTTFDRDNDIALSNCALTHHGGWWYKNCHLANPNGKYGETKHSEGVNWEPWKGHEFSIPYVELKIRPF

>MusTNXB

RDCGEELKNGPSASKTTTIFLNGNRERPLDVFCDMETDGGGWLVFQRRMDGQTDFWRDWEEYAHGFGNISGEFWLGNEALHSLTQAGDYSLRVDLRAGKEAVFAQYDFFRVDSAKENYRLHLEGYHGTAGDSMSYHSGSVFSARDRDPNNLLISCAVSYRGAWWYRNCHYANLNGLYGSTVDHQGVSWYHWKGFEFSVPFTEMKLRPR

>AnolisTNC

RDCSQALLNGETTSGLYTIYLNGDQSQPVEVYCDMTSDGGGWIVFLRRQNGAEDFYRNWRTYEAGFGDPKGEFWMGLDKLHKITSAAQYELRVDLRDHGETAYALYEKFTVGDSRTRYRLKVDGYSGTAGDSMTYHNGRSFSTYDKDNDSAITNCALSYKGAFWYKNCHRVNLMGRYGDNSHSQGVNWFHWKGHEYSIQFAEMKLRPY

>AnolisTNR

QDCAQHLMNGDTLSGIYTISLNSDLNQRIQVYCDMNTDAGGWIVFQRRQNGLTDFFRKWAEYRTGFGNLEDEFWLGLDNMHKITSQGRYELRIDMRDGQESAYAYYDKFSIGDARSLYKLRIGDYNGTAGDSLTYHQGRPFSTKDRDNDVAVTNCAMSYKGAWWYKNCHRTNLNGKYGESRHSQGINWYHWKGHEFSIPFVEMKMRPY

>AnolisTNW

ADCSQVQHNGHSINGLYTIYLNGDVNKPLQVFCDMTTDGGGWIVFQRRKTGQVDFFKRWRNYVEGFGDSTGEFWLGLEKLHELTASVPYELRVDLQTHNDSAYAVYDLFQVGPSRDRYRLAVGRYRGTAGDAMSYHHGYKFTTRDRDSDIAISNCALSHYGGWWYKNCHLANLNGKYGDNDHSAGVNWEPWKGHLYSIPFTEMKIRPR

>AnolisTNX

RDCSEESLNGPGPSRVTTIYLGGNRERPLQVYCDMETDGGGWIVFQRRMNGETDFWRDWQDYVTGFGNLTREFWLGNTALHQLTSSGDYELRVDLRAGNESVYANYQSFRVDSPADYYRLHLGSYSGTAGDAFSYHSGSVFSTRDRDPNRLIISCAVSYRGAWWYRNCHYANLNGLYANNRDHQGINWFNWKGFEFSIPFTEMKLRPR

>Xenopus_trTNW

SDCIQIQLSGNRQSGVYTIYPGGDTAKGVRVYCDQETDGGGWIVFQRRNSGKLDFYQRWRTYVEGFGDPSDEFWLGLEWIHKLTSSPGNNYEIRVDLRAGDESVYAFYRNFRVGSSKDRYKLSISDYSGTAGDGLTYHNGWKFSTWDKDNDIALTNCALSHRGAFWYKNCHLANLNGQYGETGHSQGVNWEPWKGHEFSVPFVEMKMRPN

>Xenopus_trTNR

QDCAQHMLNGDNQSGVYYIYINGDMSQSVPVYCDMATDAGGWIVFQRRQNGLTDFFRKWADYRVGFGNLEDEFWLGLDTLHQVTSQGRYELRIDMRDGQEAVYAYYNKFNIGDARSLYKLRIGDFNGTSGDSLTYHQGRPFSTKDRDNDVAVTNCASSYKGAWWYKNCHRTNLNGKYGESRHSQGINWYHWKGHEFSIPFVEMKMRPY

>Xenopus_trTNC

KDCSQALLNGEADSGLYTIYVNGDQSQPMEVYCDMSVDGGGWIVFLRRTDGSEEFYRNWKTYSAGFGNINNEFFMGLENLHKLTSLGQYELRVDLRDNDETAYAVYDKFSVGDAKSRFRLKVEGYSGTAGDSMTYHNGRSFSTFDKDNDSAITNCALSYKGAFWYKNCHRVNLMGRYGDTNHSQGVNWFHWKGHEYSIQFAEMKIRPV

>Xenopus_trTNX

RDCWEKHMNGDLQSGVFTIYLGGSKDDPLLVYCDMETDGGGWIVFQRRIDGRTDFWRNWRQYKEGFGNLTSEFWLGNIALHRLSSLAPYELRVDLRAGAEAAYAVYEDFRVEGEDKHFRLRIGAYRGNAGDSLSYHNNMIFSTRDRDAEKRILPCSISYRGAWWYKNCHYANLNGMYGNNKDHQGVNWHTWKGFEFSIPFTEMKMRPQ

>Xenopus_laTNW4L

SDCSQVQQSGNRQSGVYTIYPAGDPTKGVRAYCDQETDGGGWTVFQRRSSGKLDFYQRWRTYVEGFGDPSDEFWLGLEWIHKMTSSPATNYEIRVDLRSGDESVYALYRTFRVGTSRDRYKLSIGDYSGTAGDALTYHNGWKFSTWDKDNDIALTNCALSHRGAFWYKNCHLANLNGQYGGTGHSQGVNWEPWKGHEFSIPFAEMKMRPK

>Xenopus_laTNR4L

QDCAQHMLNGDNQSGVYYIYINGDMSQSVPVYCDMTTDGGGWIVFQRRQNGLTDFFRLWADYRAGFGNLEDEFWLGLDTLHQVTSQGRYELRIDMRDGQEAVYAYYNKFNIGDARSLYKLRIGDFNGTSGDSLTYHQGRPFSTKDRDNDVAVTNCASSYKGAWWYKNCHRTNLNGKYGESRHSQGINWYHWKGHEFSIPFVEMKMRPY

>Xenopus_laTNC8L

KDCSQALLNGETASGIYTIYANGDQSQPMEVYCDMSVDGGGWIVFLRRTDGSEDFYRNWKTYAAGFGNSNNEFFMGLENLHKLTSQGQYELRVDLRDNDETAYAIYDKFAVGDAKSRYRLRVDGYTGTAGDSMTYHNGRSFSTYDKDNDSAITNCALSYKGAFWYKNCHRVNLMGRYGDTSHSQGINWFHWKGHEYSIQFAEMKIRPV

>Xenopus_laTNC8S

KDCSQALLNGETASGIYTIYANGDQSQPMEVYCDMTVDGGGWIVFLRRTDGSEEFYRNWRTYSAGFGNSNNEFFMGLENLHKLTSQGQYELRVDLRDHDETVYAVYDKFAVGDAKSRYRLRVDGYSGTAGDSMTYHNGRSFSTYDKDNDSAITNCALSYKGAFWYKNCHRVNLMGRYGDTSHSQGVNWFHWKGHEYSIQFAEMKIRPV

>Xenopus_laTNX8L

QNCWEKLMNGEVQSGLFTIYMGGSKDDPLLVYCDMETDGGGWIVFQRRMDGKTNFWRDWNEYKMGFGNFTSEFWLGNTALHRLSSLAPYELRVDLRAGAESAHAVYEDFRIEPEDKHFRLRIGEYRGTAGDSLSYHNNMIFSTRDRDAQKRILPCAISYRGAWWYKNCHYANLNGIYGNNKDHQGMNWYTWKGFEFSIPFTEMKMRPQ

>Xenopus_laTNX8S

RDCWEKQMNGEVQSGLFTIYLGGSKDDPLLVYCDMESDGGGWIVFQRRMDGKTNFWREWNAYKEGFGNLTSEFWLGNTALHRLSSLAPYELRVDLRAGADSAYAVYEDFRVEAEDRHFRLRIGEYRGTAGDSLSYHNNMIFSTRDRNTQKRILPCAISYRGAWWYKNCHFANLNGIYGNNKDHQGVNWHTWKGFEFSIPFTEMKMRPQ

>Xenopus_laTNR4S

QDCAQHMLNGDNQSGVYYIFINGDMSQSVPVYCDMNTDGGGWIVFQRRQNGLTDFFRKWADYRVGFGNLEDEFWLGLDTLHQMTSQGRYELRIDMRDGQEAVYAYYNKFNVGDARSLYKLRIGDFNGTSGDSLTYHQGRPFSTKDKDNDVAVTNCASSYKGAWWYKNCHRTNLNGKYGESRHSQGINWFHWKGHEFSIPFVEMKMRPY

>LatimeriaTNR

QDCSQYLLNGDNLSGVYTIYLRGDLSQGVPVYCDMTTDGGGWIVFQRRQNGLTDFFRKWLDYRVGFGNLEDEFWLGLDNIHKITAQGRYELRIDMRNGQESAYATYDKFFVGDARSLYKIRLGEFNGTSGDSLTYHQGRPFSTKDRDNDVAVTNCALSYRGAWWYKNCHRANLNGKYGESRHSQGINWYHWKGHEFSIPFVEMKMRPY

>LatimeriaTNC

MDCSQTLLNGEITSGLYTIFLNGDESTPVQVYCDMTTDGGGWMVFLRRQNGNLQFYRNWKNYTLGFGNPIDEFWLGLDNLNKISSQGHYELRVDLRDGEESAYALYDRFSVGEARGRFKLQLGTYSGTAGDSLSYHQGRPFSTYDKDNDVAVTNCALSYKGAFWYKNCHRVNLMGTYGDNRHSSGVNWFHWKGHEHSIPFAEMKIRPV

>LatimeriaTNW

IDCGQVLMNGNATSGVYTIYLNNDLSRPLTVYCDMTTDGGGWIVFMRRSSGVLDFYRNWKTYEKGFGDLTDEFWLGLQGLHNLTSTQTQYELRVDLQAGSESVYATYDNFKVAPGKARYKLTVGNYRGTAGDAMSYHNGRRFTAFDQDHDISLTNCAISHHGAWWYKNCHLANLNGLYGDNTHSKGVNWEPWKGHEFSIPFVEMKLRSH

>LatimeriaTNX

QDCSEELMNGRTESGVMMIFPGGDREQPVQIFCDMETDGGGWIVFQRRMNGKTNFWRKWWDYAVGFGNLTEEFWLGKTLSRHEDTEVVIIFSFYTSCAGDSLHYHDGSIFSTKDRDPSKHIAPCALSYKGAWWYKNCHFANLNGLYGSTKDHQGINWYDWKGFEFSIPFTEMKMRPF

>ProtopterusTNR

HDCAQHLQNGDQITGGYTIYINGDPSQMVPVFCDMTTDGGGWIVFQRRQNGLTDFAKKWEEYRLGFGKLEDEFWLGLDNIYRITSQERYVLRIDMKDRQDEVYAFYDRFAIGDARTLYKLRIGEYNGTAGDSLSYHQGRPFSTMDKDNDVAVTNCALAYKGAWWYKNCHRANLNGKYGESKHSQGINWYHWKGHEFSIPYVEMKMRPY

>ProtopterusTNC

KDCSQALLNGDTTSGLYTIYLNGEESNPVQVYCDMESDGGGWIVFLRRKNGKENFYRNWKTYTAGFGDPNEEFFIGLDNLNKITSQSQYELRVDLRDGDETAYALYDAFNVAESRTRFKLTVGTYSGNAGDSLSYHQGRPFSTYDRDNDIAVTNCALSYKGAFWYRNCHRVNLMGQYGVDRHSEGVNWFHWKGHEHSISFAEMKLRPA

>PolypterusTNX

LDCSQEMMNGLVESGQATIYPGGKHSPPVQVYCDMMTAGGGWTVFQRRKNGKTNFYRSWNEYKNGFGNISDEFWLGNENIHKLASQSPQMLRVDLHTMHEHAFAVYRTFNVDKEQREFRLTVSGYSGTAGDSLLYHNGQPFSTKDRDLKKLVTRCAVSYKGGWWYKNCHEANLNGVYGNNNNQQGINWLHWKGQNFSIPFTEMKLRPL

>PolypterusTNC

RDCSQALLNGDTTSGIYTIYLNGDESQPVQVYCDMNTDGGGWMLFLRRQSGKLDFYRNWKNYSLGFGDLNDEFWLGLDNLHKITTSGQYELRVDLRDGEETAYAHYDKFSVSDGRSRYKVSVGGYSGTAGDSMTYHQGRPFSTYDKDNDVAVTNCALSYKGAFWYKNCHRVNLMGRYGDNSHSQGINWFHWKGHEYSIQFADMKIRPS

>PolypterusTNR

QNCAQHLLNGETLSGIYTVYINKDPNQGVQVYCDMTTDGGGWIVFQRRQNGLTDFARKWNDYKVGFGNLENEFWLGLDNIHRITSQGRYELRIDMRDGQESVYANYDRFFIGDARNLYKLRIGEYNGTAGDSLSYHQGRPFSTKDKDNDIAVTNCALSYKGAWWYKNCHRANLNGKYGESRHSQGINWYHWKGHEFSIPFIEMKMRPF

>PolypterusTNW

MDCTQIMKNGNRTSGIYTIYVNNDRSKPLVVYCDMTTDGGGWIVFQRRSSGKVDFLRRWKNYTQGFGDMNDEFWLGLEKIYELTNTPTQYELRVDLRAGSESVYAVYDSFKLAPSRQKYKISVGNYRGNAGDAMTYHQGRGFTTIDQDNDIALSNCAFSHRGAWWYKNCHLANLNGKYGDSTHSQGVNWEPWKGHEFSIPFVEMKIRAH

>RhincodonTNX

KDCGEEHSNGRTESSIVTLYLNGDKDKPIKAYCDMETDGGGWLVFQRRMNGKTKFIRNWKAYETGFGDLAGEHWLGLKKLHQLTAQQRYELRVDLRNGQESAYATYDNFVVEGPAQRYKLRVGKYSGNAGNSLSYHHGSNFTTVDADNDEALTNCAVSYRGGWWYKNCHRVNLNGEYGNNRDHQGVNWFSWKGFEFSIPFTEMKIRPY

>CarcharodonTNX

KDCGEERSNGRAESSVVTLYLNGNKDKPIKAYCDMSTNGGGWMVFQRRKNGKTNFMRNWKAYATGFGDLAGEHWLGLEYLHQMTSQQRYELRVDLRNDDESAYATYDNFVVESPAQKYKLKVGKYAGNAGNSLSYHHGSSFTTLDSDSDEALTNCAVSYRGGWWYKNCHRVNLNGEYGNNRDHQGVNWYGWKGFEFSIPFTEMKMRPY

>AmblyrajaTNX

KDCSEELSNGRADSGEVTLYLNGNQEKPIKVFCDMTTDGGGWIVFQRRMDGKVNFMRTWKEYVAGFGDITTEHWLGLDKLHQLTSQARFELRVDLRAGDQTAYAVYDSFALEGHAAQYRLGLGKYSGTAGNSLSYHRGSNFTTIDKDNDNSLTNCAISHRGGWWYQNCHRVNLNGEYGNNKDHQGVNWFAWKGFEFSIPFAEMKMRPW

>StegostomaTNX

KDCGEELSNGRTESSIVTLYLNRDKDKPIKAYCDMETDGGGWLVFQRRMNGKTKFIKNWKAYETGFGDLAGEHWLGLKKLHQLTAQQHYELRVDLRSGQESAYATYDNFVVEGPAQRYKLRLGKYSGNAGNSLSYHHGSNFTTVDADNDEALTNCAVSYRGGWWYKNCHRVNLNGEYGNNRDHQGVNWFSWKGFEFSIPFTEMKIRPH

>CallorhinchusTNR

WDCSQHLLNGDTVSGIYTVYLASQPQQPLQVYCDMTTDGGGWTVFQRRQNGLTDFARKWLDYRLGFGNLEDEFWLGLDNLHRITIQTRYELRVDLRDGAATAHAVYDRFYISDARDRYKLRLGDYSGTAGDSLSYHQGRPFSTRDRDNDAAVTNCALSYKGAWWYKNCHRANLNGKYAENRHSQGINWYHWKGHEKSIPFVEMKIRSG

>RhincodonTNR

WDCTQHLLNGDTISGIYTIYLNGEPQQSMQVYCDMTTDGGGWLVLQRRQNGLTDFARKWADYRVGFGNLEDEFWLGLDNIHKISAQTRYELRIDLRDGRESVYATYDRFYLSDARNLYKLRIGDYNGTAGDSLSYHQGRPFSTKDRDNDVAITNCALSYKGAWWYKNCHRANLNGKYGENRHSQGINWFSWRGHELSIPFVEMKVRPS

>AmblyrajaTNR

WDCTQHLLNGDTISGIYTIYTNGRPHLAVQVFCDMVTDNGGWTVFQRRRNGLTDFSRTWAEYRMGFGNLEDEFWLGLDNIHKISAQARYDLRIDLRDGRESVYAVYDRFYLSDARNLYKLRIGDYRGTAGDSLSYHQGRPFSTKDRDNDVAITNCALSYKGAWWYKNCHRANLNGKYGENRHSQGINWYHWKGHEISIPFIEMKIRPH

>CarcharodonTNR

WDCTQHLLNGDTISGIYTIYINGEPQQSVQVFCDMTTDGGGWTVFQRRQNGLTDFARTWADYRVGFGNLEDEFWLGLDNLHKLSAQTRYELRVDLRDGRESVYAAYDRFYLSDARNLYKLRLGDYNGTAGDSLSYHQGRPFSTKDRDNDVAITNCALSYKGAWWYKNCHRANLNGKYGESRHSQGINWYHWKGHEISIPFVEMKVRPY

>StegostomaTNR

WDCTQHLLNGDTISGIYTIYLNGELQQSMQVYCDMTTDGGGWLVLQRRQNGLTDFARKWADYRVGFGNLEDEFWLGLDNIQKISAQTRYELRIDLRDGLESVYATYDRFYLSDARNLYKLRIGDYNGTAGDSLSYHQGRPFSTKDRDNDVAITNCALSYKGAWWYKNCHRANLNGKYGENRHSQGINWFSWRGHELSIPFVEMKVRPS

>CallorhincusTNC

KDCSQTLLNGEASSGLYTIFLNGNMSDPLKVYCDMTTDGGGWVVFQRRQNGNVNFYRNWRNYSAGFGDPSDEFWLGLQSLHKITSHGRYELRVDLRDEGNSAYAVYDKFLVTDPRSRYKLHIGEYSGTAGDSLTYHQGRPFSTKDRDNDIAVTNCALSYKGAWWYKNCHRVNLNGVYGNRRHSQGINWYHWKGHEHSISFVEMKLRPH

>AmblyrajaTNC

KDCSQTLLNGETSSGVQTIFLNGNWSQPLQVYCDMNTDGGGWMVFQRRESGQVDFFRNWKNYTAGFGNPSGELWLGLENLHKITSEGRYQLRVDLQDEGDTAYAVYDKFFISDAKSRYKLHIGAYNGTAGDSLTYHQGRPFSTRDRDNDVAVTNCALSYKGAWWYKNCHRVNLNGRYGIKKHSQGINWFHWKGHEHSIAFVEMKLRPH

>RhincodonTNC

KDCSQTLLNGEASSGLSTIFLRGNRSQPLEVYCDMTTDGGGWMVFQRRQNGRVDFFRNWKNYTIGFGDPSDEFWLGLENLHKITSQDHYQLRVDLRDEGDVAYAVYDRFVISDAKSRYKLHFGRYNGTAGDSLSYHQGRPFSTKDRDNDVAVTNCALSYKGAWWYKNCHKVNLNGRYGVQSHSQGVNWYHWKGHEHSIEFVEMKLRPH

>CarcharodonTNC

KDCFQTLLNGEANSGLYTIFLNGNRSQPLQVYCDMTTDGGGWMVFQRRENGKVDFFRNWKNYTIGFGNPSDEFWLGLENLHRITSQGRYQLRVDFRDEGDSAYAVYDKFLVTDSKSRYKLHVGGYSGNAGDSLTYHNGRPFSTKDRDNDVAVTNCALSYKGAWWYKNCHRVNLNGRYGTQSHSQGVNWYHWKGHEHSIEFAEMKLRPH

>StegostomaTNC

KDCSQTLLNGEASSGLYTIFLHGNRSQPLEVYCDMTTDGGGWMVFQRRQNGRVDFFRNWKNYTNGFGDPSDEFWLGLENLHKITSQGLYQLRVDLRDKGDSAYAVYDRFLISDAKSRYKLHFGQYNGTAGDSLFYHQGRPFSTKDRDNDVAVTNCALSYKGAWWYKNCHKVNLNGRYGVQSHSQGVNWFHWKGHEYSIEFVEMKLRPH

>PleurodelesTNR

QDCSQHLMNGDTMSGVYSITINGDISQRVQVYCDMTTDGGGWIVFQRRQNGLTDFFRKWADYRSGFGNLEDEFWLGLENLHKITSQGRYELRIDMRDGQEAVYAYYDKFSIGDASSMYKLRLGDFNGTSGDSLSYHQGRPFSTKDRDNDVAVTNCAMSYKGAWWFKNCHRTNLNGKYGESRHSQGINWYHWKGHEFSIPFVEMKTRPF

>PleurodelesTNC

KDCSQALLNGETASGLYTIYLNGDKTKPQEVYCDMSEDGGGWIVFLRRVDGKEDFYRNWKAYAAGFGDPTKEFFLGLENLHQITSQGQYELRVDLRDGDETAYAVYDKFLVGDSKTRYRLKVDGYSGNAGDSMTYHNGRSFSTFDKDNDSAITNCALSYKGAFWYKNCHRVNLMGRYGDNSHSQGVNWFHWKGHEYSIQFAEMKVRPV

>PleurodelesTNW

ADCSQARQNGNTTSGMYTIYLGGDVSKPMEVYCDMSTDGGGWIVFQRRNSGKVDFYKKWRNYVDGFGDPRDEFWIGLDKLHELTNKSSVHYELRVDLRTADDLAYAQYDVFQVASSKDKYKLTVNGYKGTAGDALTYHNGWKFTTYDKDNDIALSNCAMTHKGAFWYKNCHLANPNGNYGDNVHSQGVNWEPWKGHEFSIPFIEMKIRPH

>PleurodelesTNX

RDCAEERLNGAIRSQPVTIYLKGDRSHPLRVFCDMDTDGGGWMVVQRRMNGKTNFWRNWYDYAAGFGNLTQEFWLGNENLHKLTSQGAYELRVDLRTKNESAYAVYESFRVDTQDNYYRLRLGKYSGTAGDSLSYHNNMIFSTRDRDRNKHIMPCSISYRGAWWYKNCHYANLNGLYGNNKDHQGINWYTWKGFEFSIPFTEMKMRPK

>PolyodonTNR14

QNCAQHLLNGETLSGVYTIYVNRDPSQGMQVYCDMTTDGGGWIVFQRRQNGLTDFSRRWSDYRVGFGNIEDEFWLGLDNIHRVSSQGRYELRIDMQDGQESVYASYDKFFIADARNLYKLRIGEYNGTAGDSLTYHQGRPFSTKDRDNDIAVTNCALSYKGAWWYKNCHRANLNGKYGESRHSQGINWYHWKGHEFSIPFIEMKMRPY

>PolyodonTNC49

RDCSQALLNGETSSGLYTIYLKGEESQPVQVYCDMNTDGGGWIVIVRRQNGKLQFYRNWKNYTAGFGDMNDEFWLGLDTLHKITSSGQYELRVDLRDRGESAYAQYDKFTIGDPRSRFKINVGGYSGTAGDSMSYHQGRPFSTYDKDNDIAVTNCALSYKGAFWYKNCHRVNLMGRYGDDNHSQGVNWFHWKGHEHSIEFAEMKIRPS

>PolyodonTNW14

MDCTQVMKNGNTSNGIYTIYLNSDRSQPMKVYCDMTTDGGGWIVIQRRNSGKIDFFKRWKNYTQGFGDMNEEFWLGLDKMYDLTNSQQYELRVDLRAGSESVYAVYDNFKLAPARQKYKLTIGNYRGTAGDAMTYHQGRAFTTVDRDNDIALSNCAFTHRGAWWYKNCHLANLNGQYGQTTHSVGVNWEPWKGHEFSIPFVEMKMRPR

>PolyodonTNX24

VDCSQELLNGMQKSGKTTVYLGGNRETPLQVYCDMETDGGGWIVFQRRMNGKTNFYRHWKEYKAGFGNVSEEFWLGNENLHSLTTHNPQSLRVDLRAGNETAHAVYKTFRVDSEKKHYSLHIAGYSGNAGDSLRYHDDRPFSTKDHDPKPFITRCAISYKGGWWYRNCHQVNLNGLYANSKDHQGVNWFDWKGLDFSIPFTEMKMRPA

>PolyodonTNW18

MDCTQVMKNGNTSNGIYTIYLNSDRSQPMKVYCDMTTDGGGWIVIQRRNSGKIDFFKRWKNYTQGFGDMNEEFWLGLDKMYDLTNSQQYELRVDLRAGSESVYAVYDNFKLAPARQKYKLTIGNYRGTAGDAMTYHQGRAFTTVDRDNDIALSNCAFTHRGAWWYKNCHLANLNGQYGQTTHSVGVNWEPWKGHEFSIPFVEMKMRPR

>PolyodonTNR18

QNCAQHLLNGETLSGVYTIYVNRDPSQSMQVYCDMTTDGGGWILNWDCFSSPQGLDNIHRVSSQGRYELRIDMRDGQDSVYASYDKFFVGDARNLYKLRIGEYNGTAGDSLTYHQGRPFSTKDRDNDIAVTNCALSYKGAWWYKNCHRANLNGKYGESRHSQGINWYHWKGHEFSIPFIEMKMRPY

>PolyodonTNX37

ADCSQELLNGMQESGQTTVYLGGSRETPLKVYCDMETDGGGWTVFQRRMNGKTNFYRLWKEYKAGFGNVSEEFWLGNENLHSLTTHNPQSLRVDLRAGNETAHAVYKTFRVDSEKKHYSLHIAGYSGNAGDSLRYHDGRPFSTKDHDPKPFVTRCAISYKGGWWYKNCHQVNLNGLYASNKDHQGVSWFDWKGLDFSIPFSEMKMRPT

>AcipenserTNR10

QNCAQHLLNGETLSGVYTIYVNRDPSQGMQVYCDMTTDGGGWIVFQRRQNGLTDFSRRWSDYRVGFGNIEDEFWLGLDNIHRVSSQGRYELRIDMRDGQESVYASYDKFFIGDARNLYKLRIGEYNGTAGDSLNYHQGRPFSTKDRDNDIAVTNCALSYKGAWWYKNCHRANLNGKYGESRHSQGINWYHWKGHEFSIPFIEMKMRPY

>AcipenserTNC30

RDCSQALLNGETSSGLYTIYLKGEESQPVQVYCDMATDGGGWIVIVRRQNGKLQFYRNWKNYTAGFGDMNDEFWLGLDSLHKITSSGQYELRVDLRDRGESAYAQYDKFTISDPRSRYKINVGGYSGTAGDSMSYHQGRPFSTYDKDNDIAVTNCALSYKGAFWYKNCHRVNLMGRYGDDNHSQGVNWFHWKGHEHSIEFAEMKIRPS

>AcipenserTNW10

MDCTQAMKNGKTSNGIYTIYLNSDRSQPMKVYCDMTTDGGGWIVIQRRNSGKTDFLKRWKNYTQGFGDMNEEFWLGLDKMYDLTNSQQYELRVDLRAGSESVYAIYDNFKLAPAKQKYQLTVGNYRGTAGDAMTYHQGRNFTTIDRDNDIALSNCAFTHRGAWWYKNCHLANLNGQYGQTTHSVGVNWEPWKGHEFSIPFVEMKIRPR

>AcipenserTNW11

MDCTQAMKNGKTSNGIYTIYLNSDRSQPMKVYCDMTTDGGGWIVIQRRNSGKTDFLKRWKNYTQGFGDMNEEFWLGLDKMYDLTNSQQYELRVDLRAGSESVYAIYDNFKLAPAKQKYQLTVGNYRGTAGDAMTYHQGRNFTTIDRDNDIALSNCAFTHRGAWWYKNCHLANLNGQYGQTTHSVGVNWEPWKGHEFSIPFVEMKIRPR

>AcipenserTNR11

QNCAQHLLNGETLSGVYTIYVNRDPSQGMQVYCDMTTDGGGWIVFQRRQNGLTDFSRRWSDYRVGFGNMEDEFWLGLDNIHRVSSQGRYELRIDMRDGQESVYASYDKFFIGDARNLYKLRIGEYNGTAGDSLSYHQGRPFSTKDRDNDIAVTNCALSYKGAWWYKNCHRANLNGKYGESRHSQGINWYHWKGHEFSIPFIEMKMRPY

>AcipenserTNXus

VDCSQELLNGMRESGETTVYLGGSRETPLQVYCDMETDGGGWTVFQRRMNGKTNFYRRWKEYKAGFGNVSEEFWLGNENLHSLTTHNPQSLRVDLRAGNDTAHAVYKTFRVDSEKKHYTLHIAGYSGNAGDSLRYHDDRPFSTKDHDPKPFITRCAISYKGGWWYKNCHRVNLNGLYANNKDHQGVNWFDWKGLDYSIPFSEMKMRPA

>AcipenserTNC16

RDCSQALLNGETSSGLYTIYLKGEESQPVQVYCDMATDGGGWIVIVRRQNGKLQFYRNWKNYTAGFGDMNDEFWLGLDSLHKITSSGQYELRVDLRDRGESAYAQYDKFTISDPRSRYKINVGGYSGTAGDSMSYHQGRPFSTYDKDNDIAVTNCALSYKGAFWYKNCHRVNLMGRYGDDSHSQGVNWFHWKGHEHSIEFAEMKIRPS

>OncorhynchusTNR28

QNCAQHLLNGESLNGVYTIYINRDANQGVQVYCDMTTDEGGWIVFQRRQNGLTDFSRKWSDYRVGFGNLEDEFWLGLDNIQKLSAQGRYELRIDMKDGQESVYASYDKFAIGDVRNLYKLRIGEYNGTAGDSLSYHQGRPFSTKDRDNDIAVTNCALSYKGAWWYKNCHRANLNGKYGESRHSQGINWYHWKGHEFSVPFVEMKMRPF

>OncorhynchusTNC04

RDCSQALLNGDTASGVHTIYLGGDEGQPIQVYCDMDTDGGGWIVFVRRQSGKLEFFRNWRNYTAGFGDMNDEFWLGLSNLHKITVAGQYELRVDLRDKGDVAYAQYDKFSVSDPRSRYKVHVGGYSGTAGDSMTYHHGRPFSTYDHDNDIAVTNCALSYKGAFWYKNCHRVNLMGRYGDDSHSKGVNWFHWKGHEHSIEFAEMKIRPS

>OncorhynchusTNC33

KDCSQALLNGDTTSGIYTIYLGGEESQPIQVYCDMTTDGGGWMVFLRRQSGKLEFFRNWKNYTGGFGDINAEFWFGLANLHKLTTAGQYELRVDLRDKGESAYAQYDKFTIAEPRSRYKIHIGGYSGTAGDSMTYHQGRPFSTYDNDNDIAVTNCALSYKGAFWYKNCHRVNLMGRYGDDSHSKGINWFHWKGHEHSIQFAEMKIRPV

>OncorhynchusTNW10

MDCTQIKKNGNVASGIYTIYVNSDRTKPMEVYCDMDTDGGGWVVFQRRNNGQMDFMKRWRQYMAGFGNMTDEFWLGLDNIYELTNTPTQYELRVDLGVGSEKAYAVYDNFKIAPAKQKFKLTIGEYSGTAGDAMSYHQGRPFSTVDNDNDIALGNCALTHRGAWWYKNCHLANLNGKFGDNRHSMGVNWEPWKGHLMSLDFTEMKIRPV

>OncorhynchusTNC20

KDCSQALLNGDTISGTYNIYLGGDESQPIQVYCDMTTDGGGWMVFLRRQNGNLEFFRNWKNYTGGFGDMNDEFWFGLANLHKMTASGQYELRVDLRDNGKSAYAQYDKFTIAEPRSRYKIYLGRYSGTAGDSMTYHHGRPFSTYDNDNDIAVTNCALSYKGAFWYKNCHRVNLMGKYGDNSHSKGINWFHWKGHEHSIQFVEMKIRPV

>OncorhynchusTNX31

SDCSQELLNGIHESGEVEIFPRGRQGKPVLVYCDQVTDGGGWTVFQRRMDGKTDFFRGWKNYSKGFGELSGEFWLGNENLHNLTSMAPMTLRVDLRAGDESVFAKYSAFTVDTVRRNYALKVSGYSGTAGDSMSYHNARMFSTRDRDPTPFITRCAMSYRGGWWYKNCHEANLNGLYDTSVNHQGVIWTAWKGKDLSIPFTEMKLRPT

>OncorhynchusTNR10

QNCAQHLLNGESVSGVYTIYINRDANQGVQVYCDMTTDEGGWIVFQRRQNGLTDFSRKWSDYRVGFGNLEDEFWLGLDNIQKLAAQGRYELRIDMKDGQESVYANYDKFAIGDARNLYKLRIGEYNGTAGDALIYHQGRPFSTKDRDNDIAVTNCALSYKGAWWYKNCHRANLNGKYGESRHSQGINWHNWKGHEFSIPFVEMKMRPF

>OncorhynchusTNW28

MDCTQIKTNGNMASGIYTIYINSDRTKPMDVYCDMDTDGGGWVVLQRRNNGQMDFMKRWRPYMAGFGNMTDEFWLGLDNIYELTNTPTQYELRVDLGVGSEKAYAVYDNFKIAPAKQKFKLTIGKYRGTAGDAMNYHQGRPFSTVDNDNDIALGNCALTHRGAWWYKNCHLANLNGKFGDNRHSMGVNWEPWKGHLMSLDFTEMKIRPV

>OncorhynchusTNC12

RDCSQALLNGDTASGVYTIYLGGDESQPIQVYCDMATDGGGWIVFLRRQSGKLEFFRNWRNYTAGFGDMNDEFWLGLSNLHKITVVGQYELRVDLRDKGDVAHAQYDKFSISDPRSRYKVHVGGYSGTAGDSMTYHHGRPFSTYDHDNDIAVTNCALSYKGAFWYKNCHRVNLMGRYGDDSHSKGVNWFHWKGHEHSVEFAEMKIRPS

>OncorhynchusTNX13

SDCSQELLNGMHESGEVEIFPNGRQGKPVLVYCDQVTDGGGWTVFQRRMDGKTDFFQGWENYSKGFGELSGEFWLGNENLHNLTSMAPMTLRVDLRAGDESVFAKYSAFTVDTVRKNYALKVSGYSGTAGDSMSYHNGRMFSTRDRDPAPFITRCAMSYRGGWWYKNCHQANLNGLYDTNVNHQGLIWTAWKGKDYSVPFTEMKLRPT

>OncorhynchusTNXb

TECSQELLNGALQSGEVDLYPEGKEGKAVRVYCDMETEGGGWTVFQRRMNGKTDFFRTWSDYTNGFGNLSEEFWLGNELLHNLTSMGPVSLRVDLRSGNDTAYAHYSSFTIDSVDKHYAIEVSGYTGTAGDSMRYHNGRPFSTRDKEPLPLGIHCARAYMGGWWYKNCYKTNLNGLYGTNSNNQGVVWIDWKGKDSSIPFTEMKFRPA

>DanioTNR

QNCAQHLLNGETLGGIYTIYVNRDLSQGVQVYCDMTTDGGGWIVFQRRQNGLTDFSRKWTDYKIGFGSLEDEFWLGLDNIHKIAAQGRYELRIDMKDGQESVYANYDRFSIGDSKSLYKLRIGEYSGTAGDSLSYHQSRPFSTKDKDNDIAVTNCALSYKGAWWYKNCHRANLNGKYGESRHSQGINWYHWKGHEFSIPFVEMKMRPF

>DanioTNXA

TDCSQERLNGALESGPVEVFPQGRSGRPLRVYCDMETDGGGWTVFQRRKDGKTNFFRRWREYSSGFGTLDGEFWMGNELLHNFTQSVPMELRVDLRAGSESAFARYSSFTIDTAKKHYTLRVAGYSGSAGDSLSYHSGRPFSARDRDPRPFITRCAMSYRGGWWYKNCHEANLNGLYNTSSNHQGVIWTAWKGQDFSIPFTEMKFRPA

>DanioTNXB

TDCSEVQINGMKESGEAEIYPEGKNGEPVRVYCDMETDGGAWTVFQRRMDGSTDFFRSWRDYSKGFGLLSGEFWLGNDVLHTLTSLKAMSLRIDLRSGNDTAFAQYINFNISSEANHYAIDLSGYSGTAGDSMKYHKGRPFSTKDKDPDTLSIHCAKAYMGGWWYKNCYKANLNGLYASYSDNKGVVWIDWKGKDASLPFTEMKLRPS

>DanioTNCA

KDCSQALLNGDTTSGLYTIYLRGDESQPLQVYCDMTTDGGGWIVFVRRQSGKVEFFRNWKNYTAGFGDLNDEFWLGLSNLHKITSFGQYELRVDLRDKGESAYAQYDKFSISEPRARYKVHVGGYSGTAGDSMTYHHGRPFSTYDNDNDIAVTNCALSYKGAFWYKNCHRVNIMGRYGDNSHSKGVNWFHWKGHEHSVEFAEMKIRPA

>DanioTNCB

RDCSEALLNGETSSGPYTIYINGDEKQPLRVYCDMTTDGGGWMLFLRRQSGKLNFYRNWRNYSAGFGDTSDEFWLGLSNLHKITAAKQYEIRVDLRDGSETVFAVYDRFYIGDPRSRYKIQIGAYSGTAGDSLTYHQNRPFSTYDSDNDIAITNCALSYKGAFWYKNCHRVNLMGKYGDSSHSKGINWFHWKGHEHSIPFAEMKIRPA

>DanioTNW

DGLYSDHEEWEHGEWRVHIYVNNNRSRTMQVYCDMKTDGGGWIVFQRRNTGKVDFMKKWRDYMKGFGELTEEFWLGLDKIHELTNTPTQYEARFDLGSGSDRKYAVYDNFKVAPSKQKFKLTIGSYKGNAGDAMTYHQGAPFSTVDSDNDIALGNCALTHQGAWWYKNCHLANLNGRFGDNRHSMGVNWEPWKGHLQSLDFAEIKIRPV

>ThunnusTNX10

TECSQELLNGALQSGEVDIYPQGREGQAVRVYCDMETDGGGWTVFQRRINGKTDFYRTWSEYNAGFGNLSEEFWLGNELLHNLTSIGPVSLRVDMRSGNDTAYAHYANFSIDSEEKYYTLTVSGYTGTAGDSMRYHNGRPFSARDKDPHSLGIHCAKAYMGGWWYKNCYKTNLNGLYGSNSNNQGIVWIDWKGKDSSIPFTEMKFRPS

>ThunnusTNX15

SDCSQELMNGIRISGVVEIFPQGKLGTPMKVYCDMETDGGGWTVFQRRKDGSVDFFRGWKDYVKGFGDLSGEFWMGLDSIHNLTATTRMSLRVDLRDGHESVFAKYSTFEVAKRNYRLTVGGYSGTAGDSLSYHNNRIFSTKDRDSAPFITRCAMSYRGGWWYKNCHEANLNGLYGIDVKHQGVIWTTWKGKDFSISFTEMKMRPA

>ThunnusTNW12

MDCIQIMKNGNKKSGIYTVYINNDRSKPIEVYCDMDTDGGGWLMLQRRTTGKLDFMKRWRQYIAGFGNMTDEFWIGLDKIYELTNTPTQYELRFDLGLGSERVYAVYDNFKIAPVKQKFKLTIGKYSGTAGDAMTYHQGRPWTTVDSDNDIALGNCALTHRGAWWYKNCHLANLNGKWGDNRHSMGVNWEPWKGHLTSLDFTEMKIRPL

>ThunnusTNC09

KDCSQALLNGDTSSGLYTIYLGGDESQPLQVYCDMSTDGGGWIIFLRRQSGRLEFFRNWKNYTAGFGDMNDEFWLGLSNLHKITAGGQYELRVDLRDKGETAYAQYDKFSVSEPRTRYKVHVGGYSGTAGDSMTYHHGRPFSTYDHDNDIAVTNCALSYKGAFWYKNCHRVNLMGRYGDNSHSKGVNWFHWKGHEHSIEFAEMKIRPS

>ThunnusTNC19

KDCAQILLNGETTSGLYTIYVGGEESQPIQVYCDMTTDSGGWMVLLRRQNGKLDFFRNWKNYTAGFGNMNDEFWLGLSNLHKITASGHYEVRVDMRDGGESAFAQYDKFTIAEPRTRYKISIGAYSGTAGDSMTYHQGRPFSTYDNDNDIAVTNCALSYKGAFWYKNCHRVNLMGKYGDDSHSKGINWFHWKGHEHSIEFAEMKIRPA

>ThunnusTNR12

QNCAQHLLNGETLSGVYTIYINRDPSQGVQVYCDMTTDEGGWIVFQRRQNGLTDFSRKWSDYRVGFGNLEDEFWLGLDNIQRISAQGRYELRIDMKDGQESVYANYDKFSIGDARNLYKLRIGEYNGTAGDSLSYHQGRPFSTKDRDNDIAVTNCALSYKGAWWYKNCHRANLNGKYGESRHSQGINWYHWKGHEFSIPYVEMKMRPF

>EptatretusTN1

SDCTQHALNGEESSGVFTIYISGNASQPLLVFCDMETDGGGWIVFQRRQNGRTDFFRDWKSYVTGFGDLNDEFWLGLDNLHRLTAQGHKDLRVDLRDGPTAVFAVYNDFTVGNGRTKYRLKLGKYHGTAGDSMTYHSGRPFSAPDRDNDIAVTNCATSYKGAWWYKNCHRANLNGKFGEDNHSQGINWYHWKGHEQSIPFVEMKMREH

>EptatretusTN3

KDCGQVMMNENTESGFYDVYLFNNQSQPLNVYCDMTTDGGGWLVFQRRQNGNTDFFRNWKEYKKGFGLQNLNQLTSQGRYKLRVDLRAGIETAYAQYSHFIVGNEKTFYRLQLGSYSGTAGDSLSYHQNSAFSTSDRDHDMATTNCASSYKAAWWYKNCHRVNLNGKYNENTYSQGINWYHWKGHEFSIPFVEMKMRAK

>PetromyzonTN59

RDCAEALLDGQRASGVTTVYLAGDPARPLQVYCDMETDGGGWIVLQRRQNGKTNFMRGWKEYEKGFGELTDEFWFGLRDVHALTSQGRYELRVDLRSGSEAVFATYDSFSVGEPGGLYKIRVGQYAGTAGDSMTYHQGRPFSTHDRDNDIAITNCAVSYKGAWWYKNCHRANLNGRYGDNSHSQGVNWYHWKGHEHSVDFAEMKVRPH

>PetromyzonTN22

QDCAEQSLNGETESRVYTVYVRNNESQPLLVFCDMSTDGGGWIVFQRRQNGHTEFFRDWKSYARGFGDLRDEFWLGLDNLHRLTQQGRYELRVDLHDGPEEAFALYDRFAVADAAALYRLRIGAYNGTAGDSLSYHQGRPFSAPDRDNDVAVTNCAISYKGAWWYKNCHRVNLNGKYGEASHSQGINWYHWKGHEHSIPFVEMKMRPH

>PetromyzonTN50

RDCSEALQGDQASGVFTIFLNGNASQPLPVYCDMTTDGGGWIVVQRRQNGLTDFFRNWKAYEKGFGDINDEFWLGLQNIFALTSQGRYELRVDLRDGEWGAHAVYDHFSVANASGLYRLKVGEYSGTAGDSMTYHQGRPFSTVDRDNDLAITNCAVSYHGAWWYKNCHRANLNGKYGDTSHSQGVNWYHWKGHEYSVPFVEMKIRPF

>PetromyzonTN19

RDCSQVLLNGAAAREHVVIYLRGDPARPLHVTCDMHTDGGGWTVFQRRQSGLTNFERGWQEYEEGFGEPTREFWIGLRSLHALLAQARYELRVDLGDGEASAFAQYDRFAVGDAESLYRVRIGEYSGTAGDSLSYHHGRAFSTPDRDHDGAAPHCAESYRGGWWYRNCHKANLNGRYGDTSHSRGVNWVAWRGHEGSLSFVEMKLRPH

>BranchiostomaTN

TDCGQVVANGETESGPFTVYPSDGGEPIEVWCDMETDGGGWIVFQRRQDGSVDFWRRWREYRAGFGENTGEFWLGNENIHRLSHQGDYQLRIDLADGTDAVYGEWDNFKLGSESDLYKLNIGEYSGTSGDSLTYHNNRPFSTRDKDNDVALSHCAFAYHGAWWYKNCHRSNLNGQYGDNTHSQGVNWYHWKGHEKSVPFVEMKMRSM

The following were added to the analysis found in Supplementary Figure 2:

>PhallusiaTN

KDCAEILLNGDRTNGVYTVFFDHGSETRVQCDLRTDGGGWMVVQRRINGKEDFDRDWEDYLNGFGEEDGEEFWIGLRNMHLLTRHPQELRIDLRHKSQRVHAIYSDVTISDEQSGYVISGGHYHGDAGDSMSYHFGMKFTTKDVDNDLADNRNCAAEYGGAWWFRNCHRSSLNGQYNNTRHSQGINWFTWGGFTRSIEFVEMKMRPK

>CionaTN

KDCVQVLRNGETLNKVYEIYPYNGITLKVFCDLHTDGGGWLTFQRRQDGSLSFHRSWDEYMSGFGNLTKEFWIGLQTLHELTMTNDQTLRIDMRYKDEVSYAVYRNFSLSDAATGFMLHASGYTGTAGDSLSYHDGMKFTTYDRDNDDATNRNCAKEYKGAWWFKNCHRSSLNGLYGNSRHSQGVNWYSWGGFTRSIEFVEMKLRPQ

**Supplementary File 3**

Adams and Tucker

The FReDs of representative tenascins in FASTA format used to generate Supplementary Figure 4 with Newick Tree Files.

>Xenopus_laTNX8L

QNCWEKLMNGEVQSGLFTIYMGGSKDDPLLVYCDMETDGGGWIVFQRRMDGKTNFWRDWNEYKMGFGNFTSEFWLGNTALHRLSSLAPYELRVDLRAGAESAHAVYEDFRIEPEDKHFRLRIGEYRGTAGDSLSYHNNMIFSTRDRDAQKRILPCAISYRGAWWYKNCHYANLNGIYGNNKDHQGMNWYTWKGFEFSIPFTEMKMRPQ

>Xenopus_laTNX8S

RDCWEKQMNGEVQSGLFTIYLGGSKDDPLLVYCDMESDGGGWIVFQRRMDGKTNFWREWNAYKEGFGNLTSEFWLGNTALHRLSSLAPYELRVDLRAGADSAYAVYEDFRVEAEDRHFRLRIGEYRGTAGDSLSYHNNMIFSTRDRNTQKRILPCAISYRGAWWYKNCHFANLNGIYGNNKDHQGVNWHTWKGFEFSIPFTEMKMRPQ

>AnolisTNX

RDCSEESLNGPGPSRVTTIYLGGNRERPLQVYCDMETDGGGWIVFQRRMNGETDFWRDWQDYVTGFGNLTREFWLGNTALHQLTSSGDYELRVDLRAGNESVYANYQSFRVDSPADYYRLHLGSYSGTAGDAFSYHSGSVFSTRDRDPNRLIISCAVSYRGAWWYRNCHYANLNGLYANNRDHQGINWFNWKGFEFSIPFTEMKLRPR

>Xenopus_trTNX

RDCWEKHMNGDLQSGVFTIYLGGSKDDPLLVYCDMETDGGGWIVFQRRIDGRTDFWRNWRQYKEGFGNLTSEFWLGNIALHRLSSLAPYELRVDLRAGAEAAYAVYEDFRVEGEDKHFRLRIGAYRGNAGDSLSYHNNMIFSTRDRDAEKRILPCSISYRGAWWYKNCHYANLNGMYGNNKDHQGVNWHTWKGFEFSIPFTEMKMRPQ

>GallusTNX

RDCAEEQLNGPGPSREVLIFLGGDRQRPLHVFCDMESNGGGWLVFQRRMDGGTDFWRGWEEYIHGFGNVSGEFWLGNAALHTLTASGPTELRVDLRTPSDSAFARYRDFAVSGPEDNFRLHLGAYSGTAGDALSYHAGSPFSTRDHDPRGRPRPCAVAYTGAWWYRNCHYANLNGRYGVPYDHQGINWYPWKGFEYSIPFTEMKLRPQ

>HomoTNXB

RDCGEEMQNGAGASRTSTIFLNGNRERPLNVFCDMETDGGGWLVFQRRMDGQTDFWRDWEDYAHGFGNISGEFWLGNEALHSLTQAGDYSMRVDLRAGDEAVFAQYDSFHVDSAAEYYRLHLEGYHGTAGDSMSYHSGSVFSARDRDPNSLLISCAVSYRGAWWYRNCHYANLNGLYGSTVDHQGVSWYHWKGFEFSVPFTEMKLRPR

>MusTNXB

RDCGEELKNGPSASKTTTIFLNGNRERPLDVFCDMETDGGGWLVFQRRMDGQTDFWRDWEEYAHGFGNISGEFWLGNEALHSLTQAGDYSLRVDLRAGKEAVFAQYDFFRVDSAKENYRLHLEGYHGTAGDSMSYHSGSVFSARDRDPNNLLISCAVSYRGAWWYRNCHYANLNGLYGSTVDHQGVSWYHWKGFEFSVPFTEMKLRPR

>PolypterusTNX

LDCSQEMMNGLVESGQATIYPGGKHSPPVQVYCDMMTAGGGWTVFQRRKNGKTNFYRSWNEYKNGFGNISDEFWLGNENIHKLASQSPQMLRVDLHTMHEHAFAVYRTFNVDKEQREFRLTVSGYSGTAGDSLLYHNGQPFSTKDRDLKKLVTRCAVSYKGGWWYKNCHEANLNGVYGNNNNQQGINWLHWKGQNFSIPFTEMKLRPL

>LatimeriaTNX

QDCSEELMNGRTESGVMMIFPGGDREQPVQIFCDMETDGGGWIVFQRRMNGKTNFWRKWWDYAVGFGNLTEEFWLGKTLSRHEDTEVVIIFSFYTSCAGDSLHYHDGSIFSTKDRDPSKHIAPCALSYKGAWWYKNCHFANLNGLYGSTKDHQGINWYDWKGFEFSIPFTEMKMRPF

>PleurodelesTNX

RDCAEERLNGAIRSQPVTIYLKGDRSHPLRVFCDMDTDGGGWMVVQRRMNGKTNFWRNWYDYAAGFGNLTQEFWLGNENLHKLTSQGAYELRVDLRTKNESAYAVYESFRVDTQDNYYRLRLGKYSGTAGDSLSYHNNMIFSTRDRDRNKHIMPCSISYRGAWWYKNCHYANLNGLYGNNKDHQGINWYTWKGFEFSIPFTEMKMRPK

>PolyodonTNX24

VDCSQELLNGMQKSGKTTVYLGGNRETPLQVYCDMETDGGGWIVFQRRMNGKTNFYRHWKEYKAGFGNVSEEFWLGNENLHSLTTHNPQSLRVDLRAGNETAHAVYKTFRVDSEKKHYSLHIAGYSGNAGDSLRYHDDRPFSTKDHDPKPFITRCAISYKGGWWYRNCHQVNLNGLYANSKDHQGVNWFDWKGLDFSIPFTEMKMRPA

>PolyodonTNX37

ADCSQELLNGMQESGQTTVYLGGSRETPLKVYCDMETDGGGWTVFQRRMNGKTNFYRLWKEYKAGFGNVSEEFWLGNENLHSLTTHNPQSLRVDLRAGNETAHAVYKTFRVDSEKKHYSLHIAGYSGNAGDSLRYHDGRPFSTKDHDPKPFVTRCAISYKGGWWYKNCHQVNLNGLYASNKDHQGVSWFDWKGLDFSIPFSEMKMRPT

>AcipenserTNXus

VDCSQELLNGMRESGETTVYLGGSRETPLQVYCDMETDGGGWTVFQRRMNGKTNFYRRWKEYKAGFGNVSEEFWLGNENLHSLTTHNPQSLRVDLRAGNDTAHAVYKTFRVDSEKKHYTLHIAGYSGNAGDSLRYHDDRPFSTKDHDPKPFITRCAISYKGGWWYKNCHRVNLNGLYANNKDHQGVNWFDWKGLDYSIPFSEMKMRPA

>OncorhynchusTNX31

SDCSQELLNGIHESGEVEIFPRGRQGKPVLVYCDQVTDGGGWTVFQRRMDGKTDFFRGWKNYSKGFGELSGEFWLGNENLHNLTSMAPMTLRVDLRAGDESVFAKYSAFTVDTVRRNYALKVSGYSGTAGDSMSYHNARMFSTRDRDPTPFITRCAMSYRGGWWYKNCHEANLNGLYDTSVNHQGVIWTAWKGKDLSIPFTEMKLRPT

>OncorhynchusTNX13

SDCSQELLNGMHESGEVEIFPNGRQGKPVLVYCDQVTDGGGWTVFQRRMDGKTDFFQGWENYSKGFGELSGEFWLGNENLHNLTSMAPMTLRVDLRAGDESVFAKYSAFTVDTVRKNYALKVSGYSGTAGDSMSYHNGRMFSTRDRDPAPFITRCAMSYRGGWWYKNCHQANLNGLYDTNVNHQGLIWTAWKGKDYSVPFTEMKLRPT

>OncorhynchusTNXb

TECSQELLNGALQSGEVDLYPEGKEGKAVRVYCDMETEGGGWTVFQRRMNGKTDFFRTWSDYTNGFGNLSEEFWLGNELLHNLTSMGPVSLRVDLRSGNDTAYAHYSSFTIDSVDKHYAIEVSGYTGTAGDSMRYHNGRPFSTRDKEPLPLGIHCARAYMGGWWYKNCYKTNLNGLYGTNSNNQGVVWIDWKGKDSSIPFTEMKFRPA

>ThunnusTNX10

TECSQELLNGALQSGEVDIYPQGREGQAVRVYCDMETDGGGWTVFQRRINGKTDFYRTWSEYNAGFGNLSEEFWLGNELLHNLTSIGPVSLRVDMRSGNDTAYAHYANFSIDSEEKYYTLTVSGYTGTAGDSMRYHNGRPFSARDKDPHSLGIHCAKAYMGGWWYKNCYKTNLNGLYGSNSNNQGIVWIDWKGKDSSIPFTEMKFRPS

>ThunnusTNX15

SDCSQELMNGIRISGVVEIFPQGKLGTPMKVYCDMETDGGGWTVFQRRKDGSVDFFRGWKDYVKGFGDLSGEFWMGLDSIHNLTATTRMSLRVDLRDGHESVFAKYSTFEVAKRNYRLTVGGYSGTAGDSLSYHNNRIFSTKDRDSAPFITRCAMSYRGGWWYKNCHEANLNGLYGIDVKHQGVIWTTWKGKDFSISFTEMKMRPA

>DanioTNXA

TDCSQERLNGALESGPVEVFPQGRSGRPLRVYCDMETDGGGWTVFQRRKDGKTNFFRRWREYSSGFGTLDGEFWMGNELLHNFTQSVPMELRVDLRAGSESAFARYSSFTIDTAKKHYTLRVAGYSGSAGDSLSYHSGRPFSARDRDPRPFITRCAMSYRGGWWYKNCHEANLNGLYNTSSNHQGVIWTAWKGQDFSIPFTEMKFRPA

>DanioTNXB

TDCSEVQINGMKESGEAEIYPEGKNGEPVRVYCDMETDGGAWTVFQRRMDGSTDFFRSWRDYSKGFGLLSGEFWLGNDVLHTLTSLKAMSLRIDLRSGNDTAFAQYINFNISSEANHYAIDLSGYSGTAGDSMKYHKGRPFSTKDKDPDTLSIHCAKAYMGGWWYKNCYKANLNGLYASYSDNKGVVWIDWKGKDASLPFTEMKLRPS

>RhincodonTNX

KDCGEEHSNGRTESSIVTLYLNGDKDKPIKAYCDMETDGGGWLVFQRRMNGKTKFIRNWKAYETGFGDLAGEHWLGLKKLHQLTAQQRYELRVDLRNGQESAYATYDNFVVEGPAQRYKLRVGKYSGNAGNSLSYHHGSNFTTVDADNDEALTNCAVSYRGGWWYKNCHRVNLNGEYGNNRDHQGVNWFSWKGFEFSIPFTEMKIRPY

>CarcharodonTNX

KDCGEERSNGRAESSVVTLYLNGNKDKPIKAYCDMSTNGGGWMVFQRRKNGKTNFMRNWKAYATGFGDLAGEHWLGLEYLHQMTSQQRYELRVDLRNDDESAYATYDNFVVESPAQKYKLKVGKYAGNAGNSLSYHHGSSFTTLDSDSDEALTNCAVSYRGGWWYKNCHRVNLNGEYGNNRDHQGVNWYGWKGFEFSIPFTEMKMRPY

>AmblyrajaTNX

KDCSEELSNGRADSGEVTLYLNGNQEKPIKVFCDMTTDGGGWIVFQRRMDGKVNFMRTWKEYVAGFGDITTEHWLGLDKLHQLTSQARFELRVDLRAGDQTAYAVYDSFALEGHAAQYRLGLGKYSGTAGNSLSYHRGSNFTTIDKDNDNSLTNCAISHRGGWWYQNCHRVNLNGEYGNNKDHQGVNWFAWKGFEFSIPFAEMKMRPW

>StegostomaTNX

KDCGEELSNGRTESSIVTLYLNRDKDKPIKAYCDMETDGGGWLVFQRRMNGKTKFIKNWKAYETGFGDLAGEHWLGLKKLHQLTAQQHYELRVDLRSGQESAYATYDNFVVEGPAQRYKLRLGKYSGNAGNSLSYHHGSNFTTVDADNDEALTNCAVSYRGGWWYKNCHRVNLNGEYGNNRDHQGVNWFSWKGFEFSIPFTEMKIRPH

((((((GallusTNX:0.492462,(HomoTNXB:0.0359488,MusTNXB:0.0603359)99.985:0.217512)99.3238:0.125239,AnolisTNX:0.0505401)99.985:0.25278,(AmblyrajaTNX:0.206607,(CarcharodonTNX:0.101308,(RhincodonTNX:1.04e-06,StegostomaTNX:0.0412389)99.985:0.0823279)99.985:0.176913)99.985:0.406097)21.566:0.0242057,(PleurodelesTNX:0.194459,(Xenopus_laTNX8L:0.0610023,(Xenopus_trTNX:0.114402,Xenopus_laTNX8S:0.0515769)52.0227:0.0102798)99.985:0.302796)99.985:0.0683401)35.4248:0.0307006,LatimeriaTNX:0.520166)99.985:0.14125155,(((PolyodonTNX37:0.0504806,AcipenserTNXus:0.0369964)61.2333:0.0122306,PolyodonTNX24:0.0317397)99.985:0.166438,(PolypterusTNX:0.409684,((DanioTNXB:0.336787,(ThunnusTNX10:0.125473,OncorhynchusTNXb:0.0640771)99.985:0.113734)99.985:0.223507,(DanioTNXA:0.257765,(ThunnusTNX15:0.34815,(OncorhynchusTNX31:0.0300659,OncorhynchusTNX13:0.0475744)99.985:0.0880366)99.4287:0.0933564)78.6428:0.0687731)99.985:0.229985)99.4288:0.0989494)99.985:0.17741045)99.985;

>AnolisTNW

ADCSQVQHNGHSINGLYTIYLNGDVNKPLQVFCDMTTDGGGWIVFQRRKTGQVDFFKRWRNYVEGFGDSTGEFWLGLEKLHELTASVPYELRVDLQTHNDSAYAVYDLFQVGPSRDRYRLAVGRYRGTAGDAMSYHHGYKFTTRDRDSDIAISNCALSHYGGWWYKNCHLANLNGKYGDNDHSAGVNWEPWKGHLYSIPFTEMKIRPR

>Xenopus_laTNW4L

SDCSQVQQSGNRQSGVYTIYPAGDPTKGVRAYCDQETDGGGWTVFQRRSSGKLDFYQRWRTYVEGFGDPSDEFWLGLEWIHKMTSSPATNYEIRVDLRSGDESVYALYRTFRVGTSRDRYKLSIGDYSGTAGDALTYHNGWKFSTWDKDNDIALTNCALSHRGAFWYKNCHLANLNGQYGGTGHSQGVNWEPWKGHEFSIPFAEMKMRPK

>PleurodelesTNW

ADCSQARQNGNTTSGMYTIYLGGDVSKPMEVYCDMSTDGGGWIVFQRRNSGKVDFYKKWRNYVDGFGDPRDEFWIGLDKLHELTNKSSVHYELRVDLRTADDLAYAQYDVFQVASSKDKYKLTVNGYKGTAGDALTYHNGWKFTTYDKDNDIALSNCAMTHKGAFWYKNCHLANPNGNYGDNVHSQGVNWEPWKGHEFSIPFIEMKIRPH

>ThunnusTNW12

MDCIQIMKNGNKKSGIYTVYINNDRSKPIEVYCDMDTDGGGWLMLQRRTTGKLDFMKRWRQYIAGFGNMTDEFWIGLDKIYELTNTPTQYELRFDLGLGSERVYAVYDNFKIAPVKQKFKLTIGKYSGTAGDAMTYHQGRPWTTVDSDNDIALGNCALTHRGAWWYKNCHLANLNGKWGDNRHSMGVNWEPWKGHLTSLDFTEMKIRPL

>DanioTNW

DGLYSDHEEWEHGEWRVHIYVNNNRSRTMQVYCDMKTDGGGWIVFQRRNTGKVDFMKKWRDYMKGFGELTEEFWLGLDKIHELTNTPTQYEARFDLGSGSDRKYAVYDNFKVAPSKQKFKLTIGSYKGNAGDAMTYHQGAPFSTVDSDNDIALGNCALTHQGAWWYKNCHLANLNGRFGDNRHSMGVNWEPWKGHLQSLDFAEIKIRPV

>OncorhynchusTNW28

MDCTQIKTNGNMASGIYTIYINSDRTKPMDVYCDMDTDGGGWVVLQRRNNGQMDFMKRWRPYMAGFGNMTDEFWLGLDNIYELTNTPTQYELRVDLGVGSEKAYAVYDNFKIAPAKQKFKLTIGKYRGTAGDAMNYHQGRPFSTVDNDNDIALGNCALTHRGAWWYKNCHLANLNGKFGDNRHSMGVNWEPWKGHLMSLDFTEMKIRPV

>OncorhynchusTNW10

MDCTQIKKNGNVASGIYTIYVNSDRTKPMEVYCDMDTDGGGWVVFQRRNNGQMDFMKRWRQYMAGFGNMTDEFWLGLDNIYELTNTPTQYELRVDLGVGSEKAYAVYDNFKIAPAKQKFKLTIGEYSGTAGDAMSYHQGRPFSTVDNDNDIALGNCALTHRGAWWYKNCHLANLNGKFGDNRHSMGVNWEPWKGHLMSLDFTEMKIRPV

>AcipenserTNW10

MDCTQAMKNGKTSNGIYTIYLNSDRSQPMKVYCDMTTDGGGWIVIQRRNSGKTDFLKRWKNYTQGFGDMNEEFWLGLDKMYDLTNSQQYELRVDLRAGSESVYAIYDNFKLAPAKQKYQLTVGNYRGTAGDAMTYHQGRNFTTIDRDNDIALSNCAFTHRGAWWYKNCHLANLNGQYGQTTHSVGVNWEPWKGHEFSIPFVEMKIRPR

>AcipenserTNW11

MDCTQAMKNGKTSNGIYTIYLNSDRSQPMKVYCDMTTDGGGWIVIQRRNSGKTDFLKRWKNYTQGFGDMNEEFWLGLDKMYDLTNSQQYELRVDLRAGSESVYAIYDNFKLAPAKQKYQLTVGNYRGTAGDAMTYHQGRNFTTIDRDNDIALSNCAFTHRGAWWYKNCHLANLNGQYGQTTHSVGVNWEPWKGHEFSIPFVEMKIRPR

>PolyodonTNW14

MDCTQVMKNGNTSNGIYTIYLNSDRSQPMKVYCDMTTDGGGWIVIQRRNSGKIDFFKRWKNYTQGFGDMNEEFWLGLDKMYDLTNSQQYELRVDLRAGSESVYAVYDNFKLAPARQKYKLTIGNYRGTAGDAMTYHQGRAFTTVDRDNDIALSNCAFTHRGAWWYKNCHLANLNGQYGQTTHSVGVNWEPWKGHEFSIPFVEMKMRPR

>PolyodonTNW18

MDCTQVMKNGNTSNGIYTIYLNSDRSQPMKVYCDMTTDGGGWIVIQRRNSGKIDFFKRWKNYTQGFGDMNEEFWLGLDKMYDLTNSQQYELRVDLRAGSESVYAVYDNFKLAPARQKYKLTIGNYRGTAGDAMTYHQGRAFTTVDRDNDIALSNCAFTHRGAWWYKNCHLANLNGQYGQTTHSVGVNWEPWKGHEFSIPFVEMKMRPR

>LatimeriaTNW

IDCGQVLMNGNATSGVYTIYLNNDLSRPLTVYCDMTTDGGGWIVFMRRSSGVLDFYRNWKTYEKGFGDLTDEFWLGLQGLHNLTSTQTQYELRVDLQAGSESVYATYDNFKVAPGKARYKLTVGNYRGTAGDAMSYHNGRRFTAFDQDHDISLTNCAISHHGAWWYKNCHLANLNGLYGDNTHSKGVNWEPWKGHEFSIPFVEMKLRSH

>PolypterusTNW

MDCTQIMKNGNRTSGIYTIYVNNDRSKPLVVYCDMTTDGGGWIVFQRRSSGKVDFLRRWKNYTQGFGDMNDEFWLGLEKIYELTNTPTQYELRVDLRAGSESVYAVYDSFKLAPSRQKYKISVGNYRGNAGDAMTYHQGRGFTTIDQDNDIALSNCAFSHRGAWWYKNCHLANLNGKYGDSTHSQGVNWEPWKGHEFSIPFVEMKIRAH

>GallusTNW

ADCAQMQQNGNTSSGTYTIYLNGDGSRPMQVYCDMTTDGGGWIVFQRRSTGELDFYKRWKNYVEGFGDPTGEFWLGLDQLHNLTSSSPSHYELRVDLRTASESAYAVYDFFQVASSRERYRLSVGNYRGNAGDAMTYHNGWMFTTWDRDNDVALSNCALTHHGAWWYKNCHLANLNGKYGESKHSEGVNWEPWKGHEFSIPFTEMKIRPQ

>Xenopus_trTNW

SDCIQIQLSGNRQSGVYTIYPGGDTAKGVRVYCDQETDGGGWIVFQRRNSGKLDFYQRWRTYVEGFGDPSDEFWLGLEWIHKLTSSPGNNYEIRVDLRAGDESVYAFYRNFRVGSSKDRYKLSISDYSGTAGDGLTYHNGWKFSTWDKDNDIALTNCALSHRGAFWYKNCHLANLNGQYGETGHSQGVNWEPWKGHEFSVPFVEMKMRPN

>HomoTNW

SDCSQVQQNSNAASGLYTIYLHGDASRPLQVYCDMETDGGGWIVFQRRNTGQLDFFKRWRSYVEGFGDPMKEFWLGLDKLHNLTTGTPARYEVRVDLQTANESAYAIYDFFQVASSKERYKLTVGKYRGTAGDALTYHNGWKFTTFDRDNDIALSNCALTHHGGWWYKNCHLANPNGRYGETKHSEGVNWEPWKGHEFSIPYVELKIRPH

>MusTNW

SDCSQVQQNTNAASGLYTIYLNGDASRPMQVYCDMDTDGGGWIVFQRRNTGQLDFFKRWRSYVEGFGDPMKEFWLGLDKLHNLTTGTTTRYEVRADLQTFNESAYAVYDFFQVASSKERYKLSVGKYRGTAGDALTYHNGWKFTTFDRDNDIALSNCALTHHGGWWYKNCHLANPNGKYGETKHSEGVNWEPWKGHEFSIPYVELKIRPF

(((((Xenopus_trTNW:0.0763111,Xenopus_laTNW4L:0.0932513)99.985:0.395609,PleurodelesTNW:0.240685)97.6814:0.065347,(GallusTNW:0.139678,(AnolisTNW:0.341634,(MusTNW:0.0453769,HomoTNW:0.0376955)99.985:0.153854)96.5655:0.0471645)99.5438:0.0765791)99.985:0.152567,LatimeriaTNW:0.33187)99.985:0.10224235,(((OncorhynchusTNW28:0.0383923,OncorhynchusTNW10:0.0217734)99.985:0.0917833,(ThunnusTNW12:0.137759,DanioTNW:0.470652)59.1913:0.014557)99.985:0.28909,(PolypterusTNW:0.136173,((AcipenserTNW11:1e-08,AcipenserTNW10:1e-08)99.985:0.0470668,(PolyodonTNW18:1e-08,PolyodonTNW14:1e-08)97.9643:0.0275968)99.985:0.124966)25.967:0.0258222)99.985:0.03471765)99.985;

>AnolisTNC

RDCSQALLNGETTSGLYTIYLNGDQSQPVEVYCDMTSDGGGWIVFLRRQNGAEDFYRNWRTYEAGFGDPKGEFWMGLDKLHKITSAAQYELRVDLRDHGETAYALYEKFTVGDSRTRYRLKVDGYSGTAGDSMTYHNGRSFSTYDKDNDSAITNCALSYKGAFWYKNCHRVNLMGRYGDNSHSQGVNWFHWKGHEYSIQFAEMKLRPY

>Xenopus_laTNC8L

KDCSQALLNGETASGIYTIYANGDQSQPMEVYCDMSVDGGGWIVFLRRTDGSEDFYRNWKTYAAGFGNSNNEFFMGLENLHKLTSQGQYELRVDLRDNDETAYAIYDKFAVGDAKSRYRLRVDGYTGTAGDSMTYHNGRSFSTYDKDNDSAITNCALSYKGAFWYKNCHRVNLMGRYGDTSHSQGINWFHWKGHEYSIQFAEMKIRPV

>Xenopus_laTNC8S

KDCSQALLNGETASGIYTIYANGDQSQPMEVYCDMTVDGGGWIVFLRRTDGSEEFYRNWRTYSAGFGNSNNEFFMGLENLHKLTSQGQYELRVDLRDHDETVYAVYDKFAVGDAKSRYRLRVDGYSGTAGDSMTYHNGRSFSTYDKDNDSAITNCALSYKGAFWYKNCHRVNLMGRYGDTSHSQGVNWFHWKGHEYSIQFAEMKIRPV

>Xenopus_trTNC

KDCSQALLNGEADSGLYTIYVNGDQSQPMEVYCDMSVDGGGWIVFLRRTDGSEEFYRNWKTYSAGFGNINNEFFMGLENLHKLTSLGQYELRVDLRDNDETAYAVYDKFSVGDAKSRFRLKVEGYSGTAGDSMTYHNGRSFSTFDKDNDSAITNCALSYKGAFWYKNCHRVNLMGRYGDTNHSQGVNWFHWKGHEYSIQFAEMKIRPV

>GallusTNC

KDCSQALLNGEVTSGLYTIYLNGDRTQPLQVFCDMAEDGGGWIVFLRRQNGKEDFYRNWKNYVAGFGDPKDEFWIGLENLHKISSQGQYELRVDLRDRGETAYAVYDKFSVGDAKTRYRLRVDGYSGTAGDSMTYHNGRSFSTFDKDNDSAITNCALSYKGAFWYKNCHRVNLMGRYGDNNHSQGVNWFHWKGHEYSIQFAEMKLRPS

>MusTNC

RDCSQAMLNGDTTSGLYTIYINGDKTQALEVYCDMTSDGGGWIVFLRRKNGREDFYRNWKAYAAGFGDRREEFWLGLDNLSKITAQGQYELRVDLQDHGESAYAVYDRFSVGDAKSRYKLKVEGYSGTAGDSMNYHNGRSFSTYDKDTDSAITNCALSYKGAFWYKNCHRVNLMGRYGDNNHSQGVNWFHWKGHEYSIQFAEMKLRPS

>HomoTNC

KDCSQAMLNGDTTSGLYTIYLNGDKAEALEVFCDMTSDGGGWIVFLRRKNGRENFYQNWKAYAAGFGDRREEFWLGLDNLNKITAQGQYELRVDLRDHGETAFAVYDKFSVGDAKTRYKLKVEGYSGTAGDSMAYHNGRSFSTFDKDTDSAITNCALSYKGAFWYRNCHRVNLMGRYGDNNHSQGVNWFHWKGHEHSIQFAEMKLRPS

>LatimeriaTNC

MDCSQTLLNGEITSGLYTIFLNGDESTPVQVYCDMTTDGGGWMVFLRRQNGNLQFYRNWKNYTLGFGNPIDEFWLGLDNLNKISSQGHYELRVDLRDGEESAYALYDRFSVGEARGRFKLQLGTYSGTAGDSLSYHQGRPFSTYDKDNDVAVTNCALSYKGAFWYKNCHRVNLMGTYGDNRHSSGVNWFHWKGHEHSIPFAEMKIRPV

>ProtopterusTNC

KDCSQALLNGDTTSGLYTIYLNGEESNPVQVYCDMESDGGGWIVFLRRKNGKENFYRNWKTYTAGFGDPNEEFFIGLDNLNKITSQSQYELRVDLRDGDETAYALYDAFNVAESRTRFKLTVGTYSGNAGDSLSYHQGRPFSTYDRDNDIAVTNCALSYKGAFWYRNCHRVNLMGQYGVDRHSEGVNWFHWKGHEHSISFAEMKLRPA

>PolypterusTNC

RDCSQALLNGDTTSGIYTIYLNGDESQPVQVYCDMNTDGGGWMLFLRRQSGKLDFYRNWKNYSLGFGDLNDEFWLGLDNLHKITTSGQYELRVDLRDGEETAYAHYDKFSVSDGRSRYKVSVGGYSGTAGDSMTYHQGRPFSTYDKDNDVAVTNCALSYKGAFWYKNCHRVNLMGRYGDNSHSQGINWFHWKGHEYSIQFADMKIRPS

>CallorhincusTNC

KDCSQTLLNGEASSGLYTIFLNGNMSDPLKVYCDMTTDGGGWVVFQRRQNGNVNFYRNWRNYSAGFGDPSDEFWLGLQSLHKITSHGRYELRVDLRDEGNSAYAVYDKFLVTDPRSRYKLHIGEYSGTAGDSLTYHQGRPFSTKDRDNDIAVTNCALSYKGAWWYKNCHRVNLNGVYGNRRHSQGINWYHWKGHEHSISFVEMKLRPH

>AmblyrajaTNC

KDCSQTLLNGETSSGVQTIFLNGNWSQPLQVYCDMNTDGGGWMVFQRRESGQVDFFRNWKNYTAGFGNPSGELWLGLENLHKITSEGRYQLRVDLQDEGDTAYAVYDKFFISDAKSRYKLHIGAYNGTAGDSLTYHQGRPFSTRDRDNDVAVTNCALSYKGAWWYKNCHRVNLNGRYGIKKHSQGINWFHWKGHEHSIAFVEMKLRPH

>RhincodonTNC

KDCSQTLLNGEASSGLSTIFLRGNRSQPLEVYCDMTTDGGGWMVFQRRQNGRVDFFRNWKNYTIGFGDPSDEFWLGLENLHKITSQDHYQLRVDLRDEGDVAYAVYDRFVISDAKSRYKLHFGRYNGTAGDSLSYHQGRPFSTKDRDNDVAVTNCALSYKGAWWYKNCHKVNLNGRYGVQSHSQGVNWYHWKGHEHSIEFVEMKLRPH

>CarcharodonTNC

KDCFQTLLNGEANSGLYTIFLNGNRSQPLQVYCDMTTDGGGWMVFQRRENGKVDFFRNWKNYTIGFGNPSDEFWLGLENLHRITSQGRYQLRVDFRDEGDSAYAVYDKFLVTDSKSRYKLHVGGYSGNAGDSLTYHNGRPFSTKDRDNDVAVTNCALSYKGAWWYKNCHRVNLNGRYGTQSHSQGVNWYHWKGHEHSIEFAEMKLRPH

>StegostomaTNC

KDCSQTLLNGEASSGLYTIFLHGNRSQPLEVYCDMTTDGGGWMVFQRRQNGRVDFFRNWKNYTNGFGDPSDEFWLGLENLHKITSQGLYQLRVDLRDKGDSAYAVYDRFLISDAKSRYKLHFGQYNGTAGDSLFYHQGRPFSTKDRDNDVAVTNCALSYKGAWWYKNCHKVNLNGRYGVQSHSQGVNWFHWKGHEYSIEFVEMKLRPH

>PleurodelesTNC

KDCSQALLNGETASGLYTIYLNGDKTKPQEVYCDMSEDGGGWIVFLRRVDGKEDFYRNWKAYAAGFGDPTKEFFLGLENLHQITSQGQYELRVDLRDGDETAYAVYDKFLVGDSKTRYRLKVDGYSGNAGDSMTYHNGRSFSTFDKDNDSAITNCALSYKGAFWYKNCHRVNLMGRYGDNSHSQGVNWFHWKGHEYSIQFAEMKVRPV

>OncorhynchusTNC04

RDCSQALLNGDTASGVHTIYLGGDEGQPIQVYCDMDTDGGGWIVFVRRQSGKLEFFRNWRNYTAGFGDMNDEFWLGLSNLHKITVAGQYELRVDLRDKGDVAYAQYDKFSVSDPRSRYKVHVGGYSGTAGDSMTYHHGRPFSTYDHDNDIAVTNCALSYKGAFWYKNCHRVNLMGRYGDDSHSKGVNWFHWKGHEHSIEFAEMKIRPS

>OncorhynchusTNC33

KDCSQALLNGDTTSGIYTIYLGGEESQPIQVYCDMTTDGGGWMVFLRRQSGKLEFFRNWKNYTGGFGDINAEFWFGLANLHKLTTAGQYELRVDLRDKGESAYAQYDKFTIAEPRSRYKIHIGGYSGTAGDSMTYHQGRPFSTYDNDNDIAVTNCALSYKGAFWYKNCHRVNLMGRYGDDSHSKGINWFHWKGHEHSIQFAEMKIRPV

>ThunnusTNC09

KDCSQALLNGDTSSGLYTIYLGGDESQPLQVYCDMSTDGGGWIIFLRRQSGRLEFFRNWKNYTAGFGDMNDEFWLGLSNLHKITAGGQYELRVDLRDKGETAYAQYDKFSVSEPRTRYKVHVGGYSGTAGDSMTYHHGRPFSTYDHDNDIAVTNCALSYKGAFWYKNCHRVNLMGRYGDNSHSKGVNWFHWKGHEHSIEFAEMKIRPS

>ThunnusTNC19

KDCAQILLNGETTSGLYTIYVGGEESQPIQVYCDMTTDSGGWMVLLRRQNGKLDFFRNWKNYTAGFGNMNDEFWLGLSNLHKITASGHYEVRVDMRDGGESAFAQYDKFTIAEPRTRYKISIGAYSGTAGDSMTYHQGRPFSTYDNDNDIAVTNCALSYKGAFWYKNCHRVNLMGKYGDDSHSKGINWFHWKGHEHSIEFAEMKIRPA

>DanioTNCA

KDCSQALLNGDTTSGLYTIYLRGDESQPLQVYCDMTTDGGGWIVFVRRQSGKVEFFRNWKNYTAGFGDLNDEFWLGLSNLHKITSFGQYELRVDLRDKGESAYAQYDKFSISEPRARYKVHVGGYSGTAGDSMTYHHGRPFSTYDNDNDIAVTNCALSYKGAFWYKNCHRVNIMGRYGDNSHSKGVNWFHWKGHEHSVEFAEMKIRPA

>DanioTNCB

RDCSEALLNGETSSGPYTIYINGDEKQPLRVYCDMTTDGGGWMLFLRRQSGKLNFYRNWRNYSAGFGDTSDEFWLGLSNLHKITAAKQYEIRVDLRDGSETVFAVYDRFYIGDPRSRYKIQIGAYSGTAGDSLTYHQNRPFSTYDSDNDIAITNCALSYKGAFWYKNCHRVNLMGKYGDSSHSKGINWFHWKGHEHSIPFAEMKIRPA

>OncorhynchusTNC12

RDCSQALLNGDTASGVYTIYLGGDESQPIQVYCDMATDGGGWIVFLRRQSGKLEFFRNWRNYTAGFGDMNDEFWLGLSNLHKITVVGQYELRVDLRDKGDVAHAQYDKFSISDPRSRYKVHVGGYSGTAGDSMTYHHGRPFSTYDHDNDIAVTNCALSYKGAFWYKNCHRVNLMGRYGDDSHSKGVNWFHWKGHEHSVEFAEMKIRPS

>OncorhynchusTNC20

KDCSQALLNGDTISGTYNIYLGGDESQPIQVYCDMTTDGGGWMVFLRRQNGNLEFFRNWKNYTGGFGDMNDEFWFGLANLHKMTASGQYELRVDLRDNGKSAYAQYDKFTIAEPRSRYKIYLGRYSGTAGDSMTYHHGRPFSTYDNDNDIAVTNCALSYKGAFWYKNCHRVNLMGKYGDNSHSKGINWFHWKGHEHSIQFVEMKIRPV

>AcipenserTNC16

RDCSQALLNGETSSGLYTIYLKGEESQPVQVYCDMATDGGGWIVIVRRQNGKLQFYRNWKNYTAGFGDMNDEFWLGLDSLHKITSSGQYELRVDLRDRGESAYAQYDKFTISDPRSRYKINVGGYSGTAGDSMSYHQGRPFSTYDKDNDIAVTNCALSYKGAFWYKNCHRVNLMGRYGDDSHSQGVNWFHWKGHEHSIEFAEMKIRPS

>AcipenserTNC30

RDCSQALLNGETSSGLYTIYLKGEESQPVQVYCDMATDGGGWIVIVRRQNGKLQFYRNWKNYTAGFGDMNDEFWLGLDSLHKITSSGQYELRVDLRDRGESAYAQYDKFTISDPRSRYKINVGGYSGTAGDSMSYHQGRPFSTYDKDNDIAVTNCALSYKGAFWYKNCHRVNLMGRYGDDNHSQGVNWFHWKGHEHSIEFAEMKIRPS

>PolyodonTNC49

RDCSQALLNGETSSGLYTIYLKGEESQPVQVYCDMNTDGGGWIVIVRRQNGKLQFYRNWKNYTAGFGDMNDEFWLGLDTLHKITSSGQYELRVDLRDRGESAYAQYDKFTIGDPRSRFKINVGGYSGTAGDSMSYHQGRPFSTYDKDNDIAVTNCALSYKGAFWYKNCHRVNLMGRYGDDNHSQGVNWFHWKGHEHSIEFAEMKIRPS

((((AmblyrajaTNC:0.208539,(RhincodonTNC:0.0528563,StegostomaTNC:0.054126)99.985:0.0841195)80.9644:0.0327128,CarcharodonTNC:0.0988714)97.058:0.0498703,CallorhincusTNC:0.194175)99.985:0.276549105,((ProtopterusTNC:0.279216,LatimeriaTNC:0.212268)99.985:0.108456,((AnolisTNC:0.120058,((MusTNC:0.065573,HomoTNC:0.0809153)99.985:0.136249,(GallusTNC:0.0956131,(PleurodelesTNC:0.107072,(Xenopus_trTNC:0.0674137,(Xenopus_laTNC8S:0.0345778,Xenopus_laTNC8L:0.0343923)94.9908:0.0375823)99.985:0.130192)99.985:0.0947321)96.5898:0.0255772)96.6921:0.0452746)99.985:0.189803,(PolypterusTNC:0.153385,((AcipenserTNC16:4.8e-07,(PolyodonTNC49:0.0320584,AcipenserTNC30:7.8e-07)97.0585:0.00766723)99.985:0.125398,(DanioTNCB:0.336501,(ThunnusTNC19:0.158084,((OncorhynchusTNC20:0.108282,OncorhynchusTNC33:0.0643868)99.985:0.063183,(DanioTNCA:0.0814083,(ThunnusTNC09:0.0558427,(OncorhynchusTNC12:0.0251654,OncorhynchusTNC04:0.0395407)99.985:0.090436)95.6906:0.0323208)99.9125:0.0565502)93.8127:0.0297878)91.6675:0.0376558)99.8829:0.0591738)87.5176:0.0361902)99.985:0.0568363)30.9081:0.00593531)99.985:0.003996895)99.985;

>GallusTNR

QDCAQHLMNGDTLSGVYTISINGDLSQRVQVFCDMSTDGGGWIVFQRRQNGLTDFFRKWADYRVGFGNLEDEFWLGLDNIHKITSQGRYELRIDMRDGQEAAYAYYDKFSVGDSRSLYKLRIGDYNGTSGDSLTYHQGRPFSTKDRDNDVAVTNCAMSYKGAWWYKNCHRTNLNGKYGESRHSQGINWYHWKGHEFSIPFVEMKMRPY

>Xenopus_trTNR

QDCAQHMLNGDNQSGVYYIYINGDMSQSVPVYCDMATDAGGWIVFQRRQNGLTDFFRKWADYRVGFGNLEDEFWLGLDTLHQVTSQGRYELRIDMRDGQEAVYAYYNKFNIGDARSLYKLRIGDFNGTSGDSLTYHQGRPFSTKDRDNDVAVTNCASSYKGAWWYKNCHRTNLNGKYGESRHSQGINWYHWKGHEFSIPFVEMKMRPY

>HomoTNR

QDCAQHLMNGDTLSGVYPIFLNGELSQKLQVYCDMTTDGGGWIVFQRRQNGQTDFFRKWADYRVGFGNVEDEFWLGLDNIHRITSQGRYELRVDMRDGQEAAFASYDRFSVEDSRNLYKLRIGSYNGTAGDSLSYHQGRPFSTEDRDNDVAVTNCAMSYKGAWWYKNCHRTNLNGKYGESRHSQGINWYHWKGHEFSIPFVEMKMRPY

>PleurodelesTNR

QDCSQHLMNGDTMSGVYSITINGDISQRVQVYCDMTTDGGGWIVFQRRQNGLTDFFRKWADYRSGFGNLEDEFWLGLENLHKITSQGRYELRIDMRDGQEAVYAYYDKFSIGDASSMYKLRLGDFNGTSGDSLSYHQGRPFSTKDRDNDVAVTNCAMSYKGAWWFKNCHRTNLNGKYGESRHSQGINWYHWKGHEFSIPFVEMKTRPF

>PolyodonTNR14

QNCAQHLLNGETLSGVYTIYVNRDPSQGMQVYCDMTTDGGGWIVFQRRQNGLTDFSRRWSDYRVGFGNIEDEFWLGLDNIHRVSSQGRYELRIDMQDGQESVYASYDKFFIADARNLYKLRIGEYNGTAGDSLTYHQGRPFSTKDRDNDIAVTNCALSYKGAWWYKNCHRANLNGKYGESRHSQGINWYHWKGHEFSIPFIEMKMRPY

>PolyodonTNR18

QNCAQHLLNGETLSGVYTIYVNRDPSQSMQVYCDMTTDGGGWILNWDCFSSPQGLDNIHRVSSQGRYELRIDMRDGQDSVYASYDKFFVGDARNLYKLRIGEYNGTAGDSLTYHQGRPFSTKDRDNDIAVTNCALSYKGAWWYKNCHRANLNGKYGESRHSQGINWYHWKGHEFSIPFIEMKMRPY

>AcipenserTNR10

QNCAQHLLNGETLSGVYTIYVNRDPSQGMQVYCDMTTDGGGWIVFQRRQNGLTDFSRRWSDYRVGFGNIEDEFWLGLDNIHRVSSQGRYELRIDMRDGQESVYASYDKFFIGDARNLYKLRIGEYNGTAGDSLNYHQGRPFSTKDRDNDIAVTNCALSYKGAWWYKNCHRANLNGKYGESRHSQGINWYHWKGHEFSIPFIEMKMRPY

>ThunnusTNR12

QNCAQHLLNGETLSGVYTIYINRDPSQGVQVYCDMTTDEGGWIVFQRRQNGLTDFSRKWSDYRVGFGNLEDEFWLGLDNIQRISAQGRYELRIDMKDGQESVYANYDKFSIGDARNLYKLRIGEYNGTAGDSLSYHQGRPFSTKDRDNDIAVTNCALSYKGAWWYKNCHRANLNGKYGESRHSQGINWYHWKGHEFSIPYVEMKMRPF

>DanioTNR

QNCAQHLLNGETLGGIYTIYVNRDLSQGVQVYCDMTTDGGGWIVFQRRQNGLTDFSRKWTDYKIGFGSLEDEFWLGLDNIHKIAAQGRYELRIDMKDGQESVYANYDRFSIGDSKSLYKLRIGEYSGTAGDSLSYHQSRPFSTKDKDNDIAVTNCALSYKGAWWYKNCHRANLNGKYGESRHSQGINWYHWKGHEFSIPFVEMKMRPF

>OncorhynchusTNR10

QNCAQHLLNGESVSGVYTIYINRDANQGVQVYCDMTTDEGGWIVFQRRQNGLTDFSRKWSDYRVGFGNLEDEFWLGLDNIQKLAAQGRYELRIDMKDGQESVYANYDKFAIGDARNLYKLRIGEYNGTAGDALIYHQGRPFSTKDRDNDIAVTNCALSYKGAWWYKNCHRANLNGKYGESRHSQGINWHNWKGHEFSIPFVEMKMRPF

>OncorhynchusTNR28

QNCAQHLLNGESLNGVYTIYINRDANQGVQVYCDMTTDEGGWIVFQRRQNGLTDFSRKWSDYRVGFGNLEDEFWLGLDNIQKLSAQGRYELRIDMKDGQESVYASYDKFAIGDVRNLYKLRIGEYNGTAGDSLSYHQGRPFSTKDRDNDIAVTNCALSYKGAWWYKNCHRANLNGKYGESRHSQGINWYHWKGHEFSVPFVEMKMRPF

>AcipenserTNR11

QNCAQHLLNGETLSGVYTIYVNRDPSQGMQVYCDMTTDGGGWIVFQRRQNGLTDFSRRWSDYRVGFGNMEDEFWLGLDNIHRVSSQGRYELRIDMRDGQESVYASYDKFFIGDARNLYKLRIGEYNGTAGDSLSYHQGRPFSTKDRDNDIAVTNCALSYKGAWWYKNCHRANLNGKYGESRHSQGINWYHWKGHEFSIPFIEMKMRPY

>RhincodonTNR

WDCTQHLLNGDTISGIYTIYLNGEPQQSMQVYCDMTTDGGGWLVLQRRQNGLTDFARKWADYRVGFGNLEDEFWLGLDNIHKISAQTRYELRIDLRDGRESVYATYDRFYLSDARNLYKLRIGDYNGTAGDSLSYHQGRPFSTKDRDNDVAITNCALSYKGAWWYKNCHRANLNGKYGENRHSQGINWFSWRGHELSIPFVEMKVRPS

>AmblyrajaTNR

WDCTQHLLNGDTISGIYTIYTNGRPHLAVQVFCDMVTDNGGWTVFQRRRNGLTDFSRTWAEYRMGFGNLEDEFWLGLDNIHKISAQARYDLRIDLRDGRESVYAVYDRFYLSDARNLYKLRIGDYRGTAGDSLSYHQGRPFSTKDRDNDVAITNCALSYKGAWWYKNCHRANLNGKYGENRHSQGINWYHWKGHEISIPFIEMKIRPH

>CarcharodonTNR

WDCTQHLLNGDTISGIYTIYINGEPQQSVQVFCDMTTDGGGWTVFQRRQNGLTDFARTWADYRVGFGNLEDEFWLGLDNLHKLSAQTRYELRVDLRDGRESVYAAYDRFYLSDARNLYKLRLGDYNGTAGDSLSYHQGRPFSTKDRDNDVAITNCALSYKGAWWYKNCHRANLNGKYGESRHSQGINWYHWKGHEISIPFVEMKVRPY

>StegostomaTNR

WDCTQHLLNGDTISGIYTIYLNGELQQSMQVYCDMTTDGGGWLVLQRRQNGLTDFARKWADYRVGFGNLEDEFWLGLDNIQKISAQTRYELRIDLRDGLESVYATYDRFYLSDARNLYKLRIGDYNGTAGDSLSYHQGRPFSTKDRDNDVAITNCALSYKGAWWYKNCHRANLNGKYGENRHSQGINWFSWRGHELSIPFVEMKVRPS

>CallorhinchusTNR

WDCSQHLLNGDTVSGIYTVYLASQPQQPLQVYCDMTTDGGGWTVFQRRQNGLTDFARKWLDYRLGFGNLEDEFWLGLDNLHRITIQTRYELRVDLRDGAATAHAVYDRFYISDARDRYKLRLGDYSGTAGDSLSYHQGRPFSTRDRDNDAAVTNCALSYKGAWWYKNCHRANLNGKYAENRHSQGINWYHWKGHEKSIPFVEMKIRSG

>LatimeriaTNR

QDCSQYLLNGDNLSGVYTIYLRGDLSQGVPVYCDMTTDGGGWIVFQRRQNGLTDFFRKWLDYRVGFGNLEDEFWLGLDNIHKITAQGRYELRIDMRNGQESAYATYDKFFVGDARSLYKIRLGEFNGTSGDSLTYHQGRPFSTKDRDNDVAVTNCALSYRGAWWYKNCHRANLNGKYGESRHSQGINWYHWKGHEFSIPFVEMKMRPY

>ProtopterusTNR

HDCAQHLQNGDQITGGYTIYINGDPSQMVPVFCDMTTDGGGWIVFQRRQNGLTDFAKKWEEYRLGFGKLEDEFWLGLDNIYRITSQERYVLRIDMKDRQDEVYAFYDRFAIGDARTLYKLRIGEYNGTAGDSLSYHQGRPFSTMDKDNDVAVTNCALAYKGAWWYKNCHRANLNGKYGESKHSQGINWYHWKGHEFSIPYVEMKMRPY

>PolypterusTNR

QNCAQHLLNGETLSGIYTVYINKDPNQGVQVYCDMTTDGGGWIVFQRRQNGLTDFARKWNDYKVGFGNLENEFWLGLDNIHRITSQGRYELRIDMRDGQESVYANYDRFFIGDARNLYKLRIGEYNGTAGDSLSYHQGRPFSTKDKDNDIAVTNCALSYKGAWWYKNCHRANLNGKYGESRHSQGINWYHWKGHEFSIPFIEMKMRPF

>MusTNR

QDCAQHLMNGDTLSGVYTIFLNGELSHKLQVYCDMTTDGGGWIVFQRRQNGQTDFFRKWADYRVGFGNLEDEFWLGLDNIHRITAQGRYELRVDMRDGQEAVFAYYDKFAVEDSRSLYKIRIGSYNGTAGDSLSYHQGRPFSTEDRDNDVAVTNCAMSYKGAWWYKNCHRTNLNGKYGESRHSQGINWYHWKGHEFSIPFVEMKMRPY

>Xenopus_laTNR4L

QDCAQHMLNGDNQSGVYYIYINGDMSQSVPVYCDMTTDGGGWIVFQRRQNGLTDFFRLWADYRAGFGNLEDEFWLGLDTLHQVTSQGRYELRIDMRDGQEAVYAYYNKFNIGDARSLYKLRIGDFNGTSGDSLTYHQGRPFSTKDRDNDVAVTNCASSYKGAWWYKNCHRTNLNGKYGESRHSQGINWYHWKGHEFSIPFVEMKMRPY

>Xenopus_laTNR4S

QDCAQHMLNGDNQSGVYYIFINGDMSQSVPVYCDMNTDGGGWIVFQRRQNGLTDFFRKWADYRVGFGNLEDEFWLGLDTLHQMTSQGRYELRIDMRDGQEAVYAYYNKFNVGDARSLYKLRIGDFNGTSGDSLTYHQGRPFSTKDKDNDVAVTNCASSYKGAWWYKNCHRTNLNGKYGESRHSQGINWFHWKGHEFSIPFVEMKMRPY

>AnolisTNR

QDCAQHLMNGDTLSGIYTISLNSDLNQRIQVYCDMNTDAGGWIVFQRRQNGLTDFFRKWAEYRTGFGNLEDEFWLGLDNMHKITSQGRYELRIDMRDGQESAYAYYDKFSIGDARSLYKLRIGDYNGTAGDSLTYHQGRPFSTKDRDNDVAVTNCAMSYKGAWWYKNCHRTNLNGKYGESRHSQGINWYHWKGHEFSIPFVEMKMRPY

(((CallorhinchusTNR:0.301774,(StegostomaTNR:0.0215021,RhincodonTNR:2.5e-07)99.985:0.0549479)73.8497:0.0176997,(CarcharodonTNR:0.0385457,AmblyrajaTNR:0.144623)95.7752:0.0202984)99.985:0.08580295,((ProtopterusTNR:0.275556,(LatimeriaTNR:0.124262,((Xenopus_laTNR4S:0.0436458,(Xenopus_trTNR:0.0142222,Xenopus_laTNR4L:0.0146105)0:5.8e-07)99.985:0.104225,(PleurodelesTNR:0.120066,((GallusTNR:0.0148457,(HomoTNR:0.0377888,MusTNR:0.0367044)99.985:0.111184)57.185:0.0137117,AnolisTNR:0.0911644)74.8485:0.0115543)98.4408:0.0328008)65.2361:0.0366895)99.2781:0.0467395)61.6413:0.016944,((DanioTNR:0.12358,(ThunnusTNR12:0.00933821,(OncorhynchusTNR10:0.0449165,OncorhynchusTNR28:0.0287861)99.985:0.0416236)99.5153:0.0169722)90.3211:0.0190276,(PolypterusTNR:0.0839413,(AcipenserTNR11:1.05e-06,(AcipenserTNR10:0.00452641,(PolyodonTNR14:0.0147135,PolyodonTNR18:0.117757)40.9481:0.00420861)85.8587:0.0120282)99.985:0.0508501)27.8112:0.0158717)99.985:0.0548143)99.985:0.09786405)99.985;


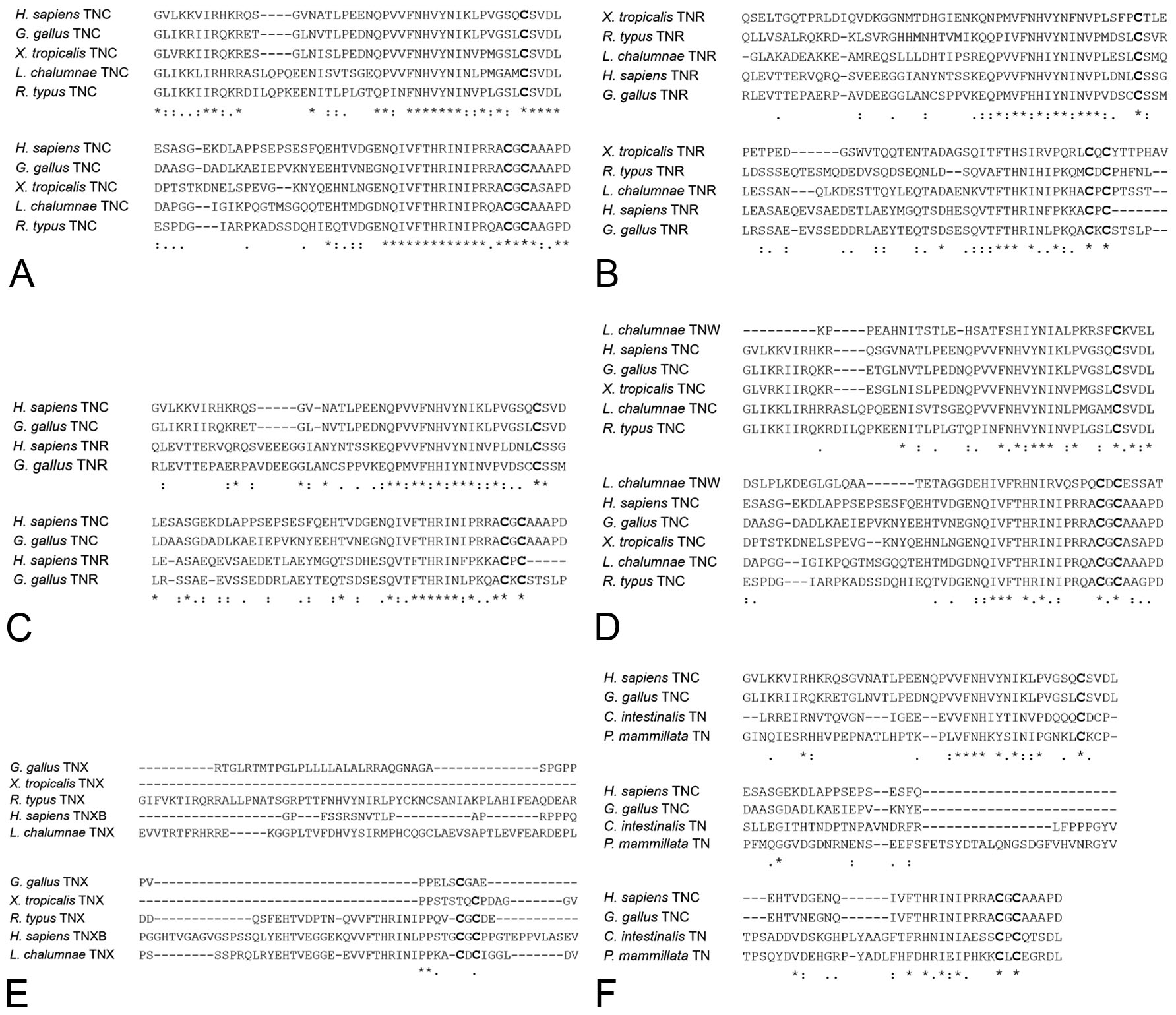


**Supplementary Figure 1 (Adams and Tucker)** Alignment of tenascin assembly domains from selected tenascins. See text for details.


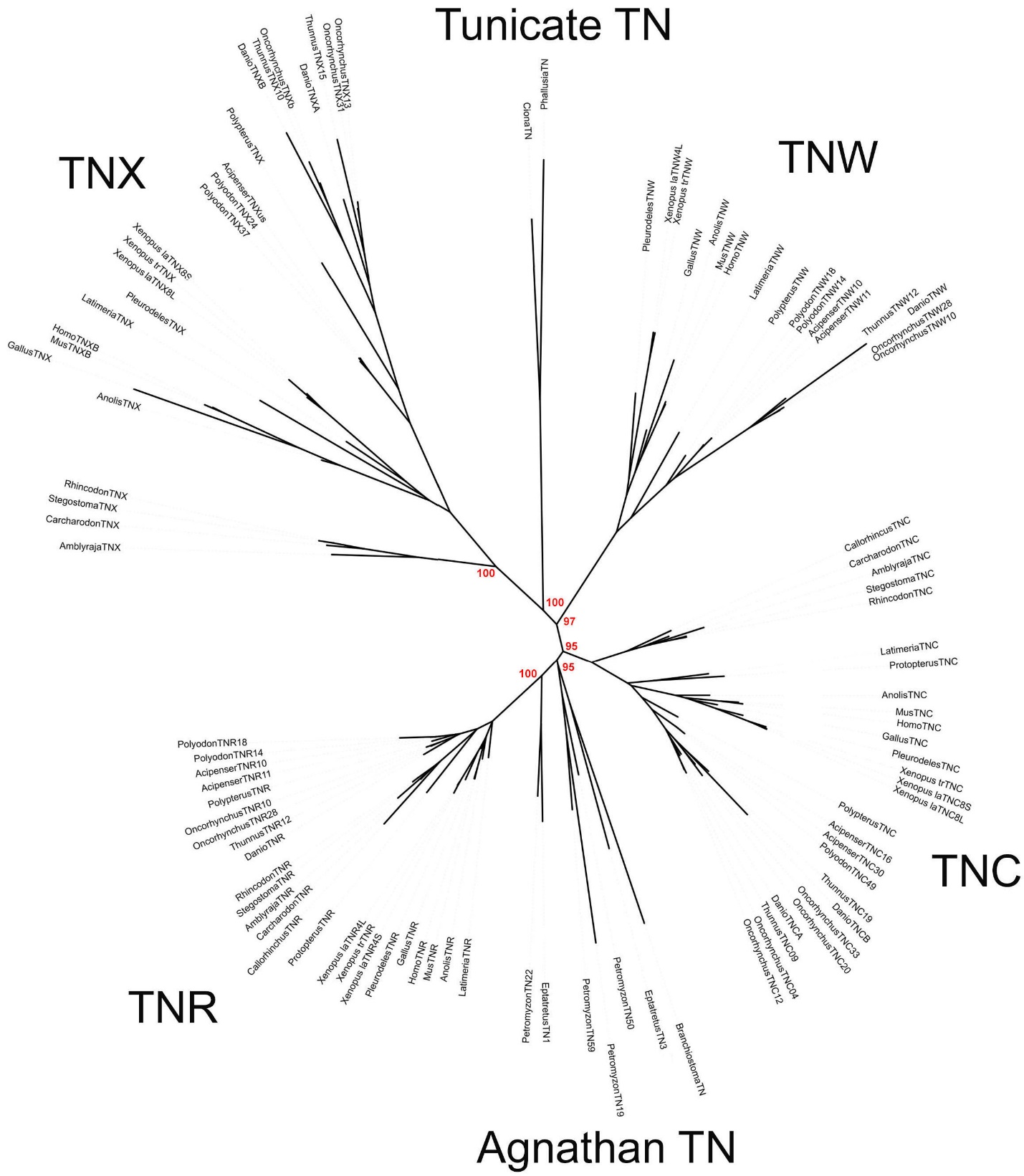


**Supplementary Figure 2 (Adams and Tucker)** Phylogenetic tree constructed following alignment of FReD sequences from selected organisms, as seen in Figure 8 but with tunicate sequences included. Branch support for representative nodes is indicated. See text for details.


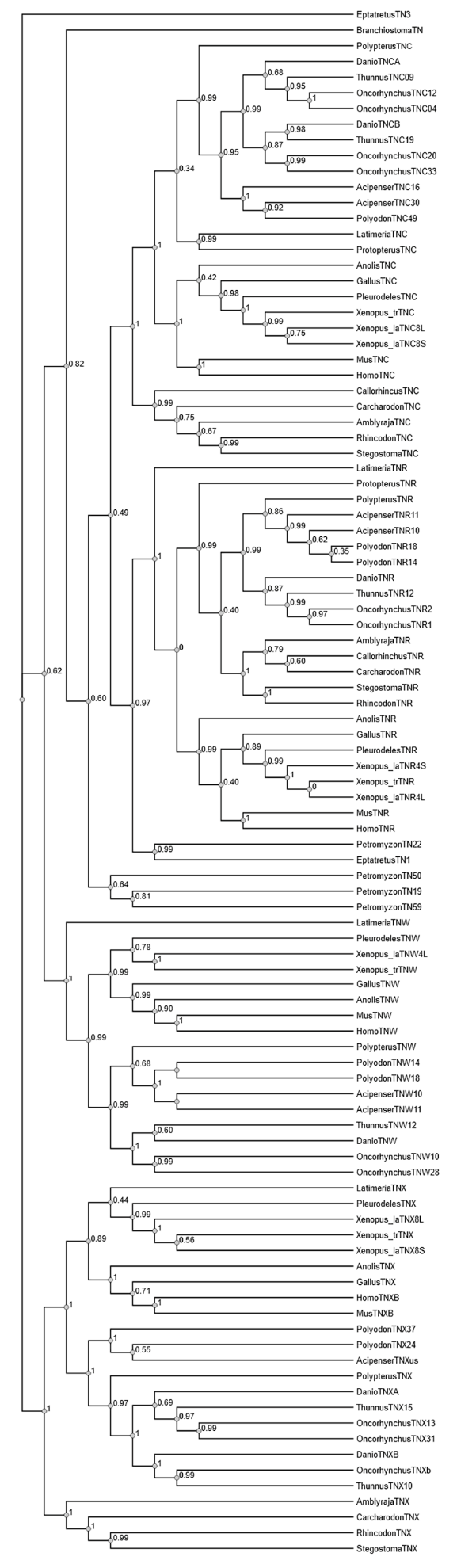


**Supplementary Figure 3 (Adams and Tucker)** Phylogenetic tree constructed using the same parameters as the unrooted tree in Figure 8, here displayed as a dendrogram. Branch support for representative nodes are more easily read on a tree displayed in this format than the unrooted tree. See text for details.


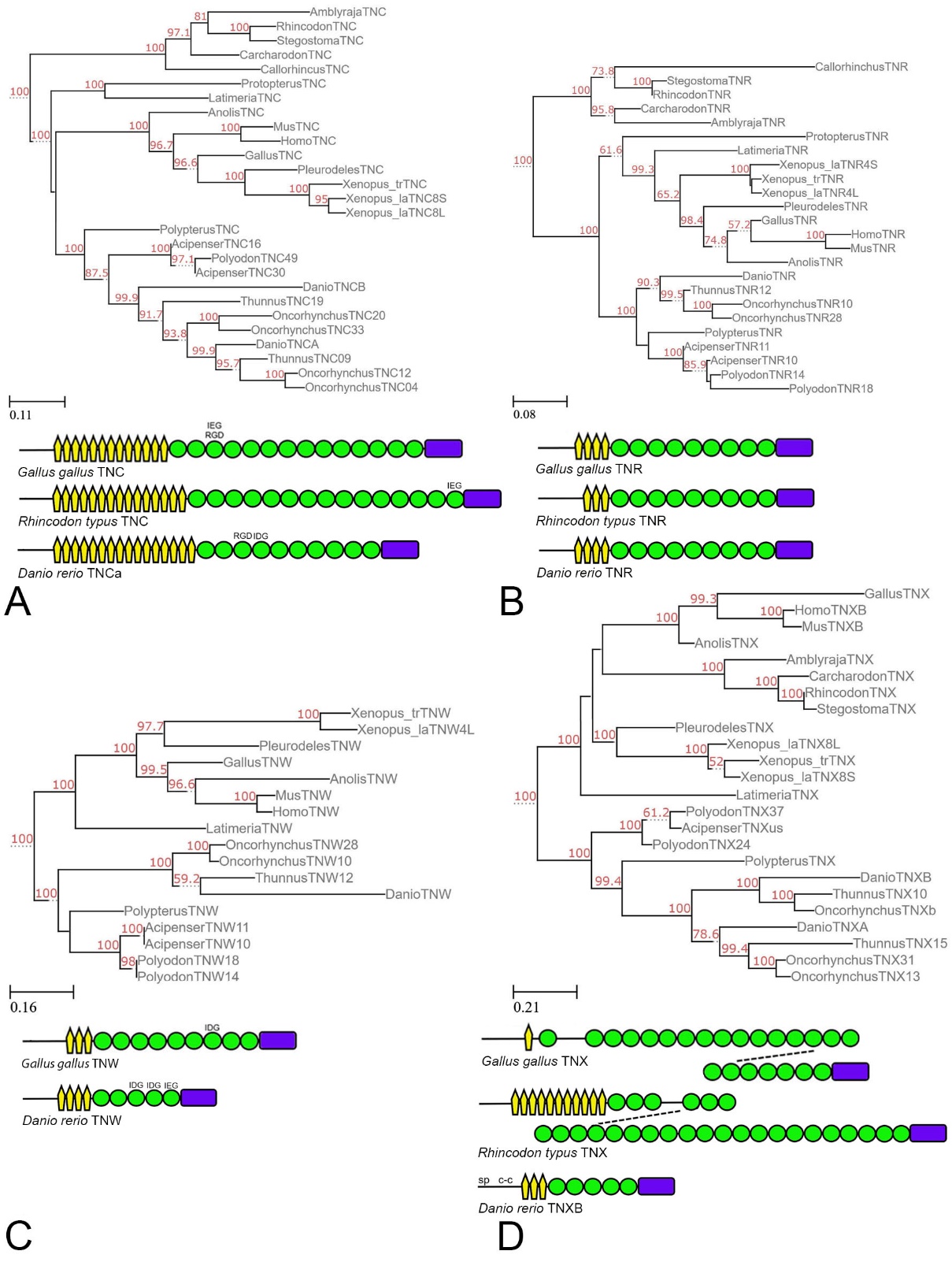


**Supplementary Figure 4 (Adams and Tucker)** Phylogenetic trees based on FReD amino acid sequences (208 residues) from TNC (A), TNR (B), TNW (C) and TNX (D) from representative gnathostomes, with stick diagrams illustrating tenascin domain organization from the chicken (*Gallus gallus*), whale shark (*Rhincodon typus*) and zebrafish (*Danio rerio*). *Callorhincus* sequence used for rooting TNC and TNR trees, and *Latimeria* sequence for TNW and TNX trees. See text for details.


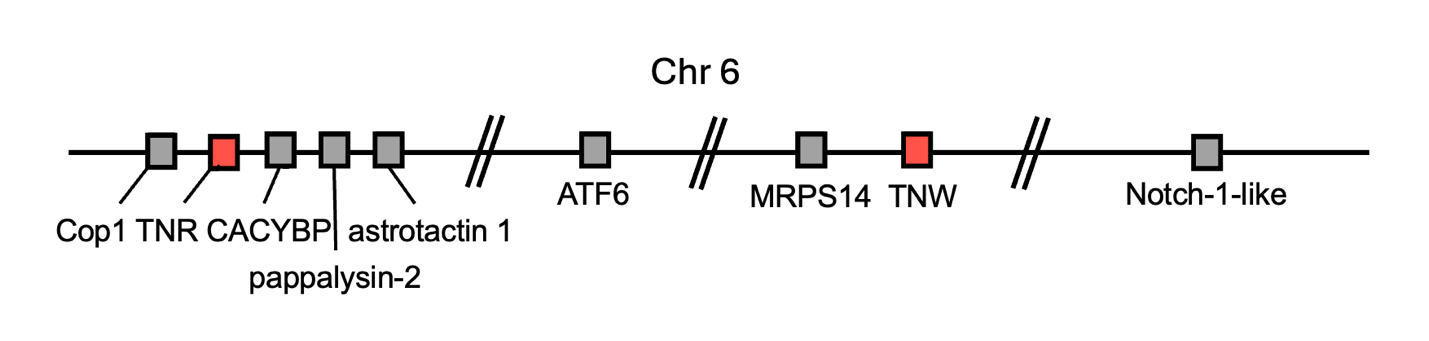


**Supplementary Figure 5 (Adams and Tucker)** Relationships between *Latimeria chalumnae* TNR and TNW genes on chromosome 6 and other relevant genes. See text for details.


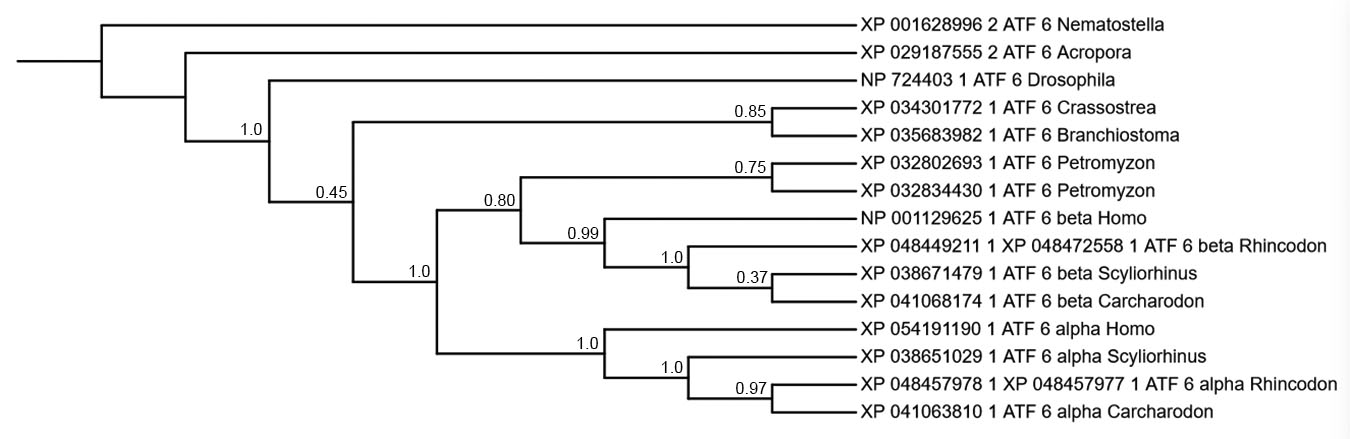


**Supplementary Figure 6 (Adams and Tucker)** Phylogenetic tree constructed following alignment of ATF6 amino acid sequences (690 residues) from selected organisms, with the *Nematostella* sequence used to root the tree. ATF6 from *Branchiostoma* clusters with the ATF6 sequences from non-chordate invertebrates. See text for details.

**Supplementary Table 1 (Adams and Tucker)** Tenascins from representatives of the Class Chondrichthyes

_________________________________________________________________________________________

Subclass Species TN Motif(s) Accession no. Adjacent gene(s)

­­­­­­­­­­­­­­­­­­­­_________________________________________________________________________________________

Holocephali *Callorhinchus milii* TNC IDG XP_042193216 *TESK2, Pappa1*

(Ghost shark) TNR XP_007901205 *RABGAP1, Cop1*

Elasmobranchii *Carcharodon carcharias* TNC IEG XP_041050030 *TESK2, Pappa1*

(Great white shark) TNR* XP_041063734 *ABT1-like, Cop1*

TNX KGD XP_041068478 *Cyp21, tRNA tryptophan*

*Rhincodon typus* TNC IEG XP_048469310 *TESK2, Pappa1*

(Whale shark) TNR XP_048458101 *ABT1-like, Cop1*

TNX XP_048472635, *Cyp21***, *Butyrophilin S1A*

XP_048472633

*Amblyraja radiata* TNC IEG XP_032905081 *TESK2, DD-CR2*

(Thorny skate) TNR XP_032885028 *PDE4B, Cop1*

TNX* XP_032872802 *Cyp21*

*Scyliorhinus canicula* TNC IEG XP_038637877 *TESK2, tRNA serine*

(Small-spotted catshark) TNR IDG XP_038651119 *ABT1-like, Cop1*

TNX XP_038671561, *Cyp21***, *Butyrophilin 10*

XP_038644573

*Stegostoma fasciatum* TNC IEG (X2), RGD XP_048414875 *TESK2, Pappa1*

(Zebra shark) TNR XP_048395939 *ABT1, Cop1*

TNX XP_048375763, *Cyp21, ZBP A33*

XP_048375789,

XP_048375791

_________________________________________________________________________________________

*Partial predicted sequence

**Overlapping with *TNX* on the complementary strand

**Supplementary Table 2 (Adams and Tucker)** Tenascins from representatives of the Class Sarcopterygii

_________________________________________________________________________________________

Class Species TN Motif(s) Accession no. Adjacent gene(s)

_________________________________________________________________________________________­­­­­­­­­­­­­­­­­­­­

Actinistia *Latimeria chalumnae* TNC IDG XP_064420300 *Papp1*, *Astn2*

(West Indian Ocean TNR XP_064415793 *Cop1*

Coelacanth) TNW IEG XP_064416217 *MRPS14*

TNX XP_064408017 *Cyp21, Atfgb*

Dipnoi *Protopterus annectens* TNC IDG, RGD XP_043914544 *PAPPA1, Tnsf8*

(West African lungfish) TNR XP_043941895 *CC2D1B, Cop1*

TNX XP_043939105 *TMP26-like, sec1**

_________________________________________________________________________________________­­­­­­­­­­­­­­­­­­­­

* Flanked by *ATF6-beta* and *C4b*

**Supplementary Table 3 (Adams and Tucker)** Tenascins from non-teleost species of the Class Actinopterygii

_________________________________________________________________________________________

Species TN (Chr) EGFs FN3s Motif(s) Accession no. Adjacent gene(s)

­­­­­­­­­­­­­­­­­­­­_________________________________________________________________________________________

*Polypterus senegalus* TNC (13) 10 14 XP_039630763 *DPP7, TRAF2A*

(Senegal bichir) TNR (14) 4 9 XP_039591531 *TNW, Cop1*

TNW (14) 3 15 IDG XP_039591331 *MRPS14, TNR*

TNX (11) 10 7 XP_039624658 *MMR1-like, C4b*

*Polyodon spathula* TNC (49) 17 14 XP_041094567 *Kelch9, GAPVD1* (American paddlefish) TNR (14) 4 9 XP_041126789 *TNW14, Cop1*

TNR (18) 4 9 XP_041133374 *TNW18, Cop1*

TNW (14) 3 18 IDG (X11) XP_041134143 *MRPS14, TNR14*

TNW (18) 3 19 IDG (X14) XP_041134141 *MRPS14, TNR18*

TNX (24) 13 6 IDG XP_041082464 *C4b, prot b7**

TNX (37) 6 6 IDG XP_041091081 *AGPAa, prot b7*

*Acipenser ruthenus* TNC (30) 15 10 RGD XP_034764758 *TESK2****, TRAF2A*

(Sterlet sturgeon) TNC (16) 16 10 RGD XP_034783779 *TESK1****, TRAF2A*

TNR (10) 4 9 XP_033865683 *TNW10, Cop1*

TNR (11) 4 9 XP_033884115 *TNW11, Cop1*

TNW (10) 3 11 IDG (X6) XP_033858340 *MRPS14, TNR10*

TNW (11) 3 5 IDG XP_033883716 *MRPS14, TNR11*

TNX (US†) 12 6 IDG XP_034771618 *C4-like*

_________________________________________________________________________________________

**Cyp21a* lies 3 genes away on the same strand as TNX

***Kelch9* is adjacent to *TESK2* and *TESK1*

†Unplaced scaffold.

**Supplementary Table 4 (Adams and Tucker)** Tenascins from representatives of the Infraclass Teleostei

_________________________________________________________________________________________

Species TN (Chr) EGFs FN3s Motif(s) Accession no. Adjacent genes

­­­­­­­­­­­­­­­­­­­­_________________________________________________________________________________________

*Danio rerio* TNCa (5) 13 16 RGD, IDG XP_021332057 *TRAF2A*

(Zebrafish) TNCb (5) 16 10 RGE NP_001299845 *b3galt8, TRAF2A*

TNR (2) 4 9 NP_919364 *TNW, Cop1*

TNW (2) 3 6 IDG (X3), IEG CAA04755 *MRPS14, TNR*

TNX* (19) 3 5 XP_021323869 *zgc:85936, UTP18sspc*

TNX** (16) 2 5 XP_021322558 *Cyp21a, C4b*

*Thunnus maccoyii* TNC (19) 13 16 XP_042251735 *Pik3R1, TRAF2A*

(Southern bluefin tuna) TNC (9) 12 10 RGE XP_042276103 *b3galt8, TRAF2A*

TNR (12) 4 9 XP_042285133 *TNW, ZFP8-like*

TNW (12) 3 11 IEG, IDG (X8) XP_042283437 *MRPS14, TNR*

TNX (15) 10 5 XP_042291908 *MHC I, tRNA proline*

TNX (10) 1 4 XP_042280668 *Cyp21, C4b*

*Oncorhynchus tshawytscha* TNC (LG04†) 12 9 RGE XP_042176645 *b3galt8, TRAF2A*

(Chinook salmon) TNC (LG12) 12 10 RGE XP_024298273 *TESK1, Rab-6*

TNC (LG20) 13 16 XP_024237862 *Pik3R1, TRAF2A*

TNC (LG33) 20 8 RGD XP_024252858 *Pik3R1, TRAF2A*

TNR (LG28) 4 9 XP_024247414 *TNW28, Cop1*

TNR (LG10) 4 9 XP_024291360 *TNW10, Cop1*

TNW (LG28) 4 11 IDG (X7) XP_042164204 *MRPS14, TNR28*

TNW (LG10) 3 8 IDG (X6) XP_042184708 *MRPS14, TNR10*

TNX (LG13) 11 6 XP_042151520 *ATF-6b, prot b7*

TNX (LG31) 7 5 XP_024251607 *ATF-6b, prot b10*

TNXb (US‡) 1 4 XP_042174733 *Cyp21, C4b*

_________________________________________________________________________________________

*TNXB in GeneID

**TNXBa in GeneID, named “serine-rich adhesin for platelets-like isoform X1” by NCBI

†Linkage group

‡Unplaced scaffold

**Supplementary Table 5** **(Adams and Tucker)** Tenascins from *Xenopus tropicalis* and *X. laevis*

_________________________________________________________________________________________

Species TN (Chr) Accession no. Adjacent genes (from GeneID)

­­­­­­­­­­­­­­­­­­­­_________________________________________________________________________________________

*Xenopus tropicalis* TNC (8) XP_004916768 *myosin-4, tktl2, TNC, cyclin-O pB-like, tnfsf8*

TNR (4) NP_001107287 *TNW, KIAA0040, TNR, cop1, pappa2, astn1*

TNW (4) XP_017949080 *cacybp, mrps14, TNW, KIAA0040, TNR*

TNX (8) XP_031747219 *c4a, psmb7, cenpa, cyp21, TNX, atf6b, prrt1*

*Xenopus laevis*  TNC (8L) XP_018085080 *tnfs15, tnfs8, TNC, tktl2, myosin-4*

TNC (8S) XP_018088380 *tnfs15, TNC, myh3, bicaudalD2, lamina ap2*

TNR (4L) XP_041446423 *spns2, TNW, TNR, cop1, pappa2, astn1*

TNR (4S) XP_041417431 *cacybp, mrps14, KIAA0040, TNR, cop1, pappa2*

TNW (4L) XP_018113965 *cacybp, spns2, TNW, TNR, cop1, pappa2*

TNX (8L) XP_041428807 *c4a, psmb7, cenpa, cyp21, TNX, atf6b, prrt1*

TNX (8S) XP_041431172 *egfl8, psmb7, TNX, atf6b, fkbp1, prrt1*

**Supplementary Table 6 (Adams and Tucker)** Gnathostome tenascins: Summary

_________________________________________________________________________________________

Taxonomy TNC TNR TNW TNX

­­­­­­­­­­­­­­­­­­­­_________________________________________________________________________________________

Class Chondrichthyes

Subclass Holocephali

*Callorhinchus milii* 1 1

Subclass Elasmobranchii

*Scyliorhinus canicula* 1 1 1

*Stegostoma fasciatum* 1 1 1

*Amblyraja radiata* 1 1 1

*Scyliorhinus canicular* 1 1 1

*Rhincodon typus* 1 1 1

*Carcharodon carcharias* 1 1 1

Superclass Osteichthyes

Class Actinistia

*Latimeria chalumnae* 1 1 1 1

Class Dipnoi

*Protopterus annectens* 1 1 1

Class Actinopterygii

*Polypterus senegalus* 1 1 1 1

Subclass Chondrostei

*Acipenser ruthenus* 2 2 2 1

*Polyodon spathula* 1 2 2 2

Infraclass Teleosteii

*Danio rerio* 2 1 1 2

*Thunnus maccoyii* 2 1 1 2

*Oncorhynchus tshawytscha* 4 2 2 3

Superclass Tetrapoda

Class Amphibia

*Pleurodeles waltl* 1 1 1 1

*Xenopus tropicalis* 1 1 1 1

*Xenopus laevis* 2 2 1 2

Class Reptilia

*Anolis carolinensis* 1 1 1 1

Class Aves

*Gallus gallus* 1 1 1 1

Class Mammalia

*Mus musculus* 1 1 1 1

*Homo sapiens* 1 1 1 1
